# Direct evidence for hula twist and single-bond rotation photoproducts

**DOI:** 10.1038/s41467-018-04928-9

**Published:** 2018-06-28

**Authors:** Aaron Gerwien, Monika Schildhauer, Stefan Thumser, Peter Mayer, Henry Dube

**Affiliations:** Department of Chemistry, Ludwig-Maximilians-Universität München and Munich Center for Integrated Protein Science CIPSM, Butenandtstrasse 5-13, 81377 München, Germany

## Abstract

Photoisomerization reactions are quintessential processes driving molecular machines and motors, govern smart materials, catalytic processes, and photopharmacology, and lie at the heart of vision, phototaxis, or vitamin production. Despite this plethora of applications fundamental photoisomerization mechanisms are not well understood at present. The famous hula-twist motion—a coupled single and double-bond rotation—was proposed to explain proficient photoswitching in restricted environments but fast thermal follow-up reactions hamper identification of primary photo products. Herein we describe an asymmetric chromophore possessing four geometrically distinct diastereomeric states that do not interconvert thermally and can be crystallized separately. Employing this molecular setup direct and unequivocal evidence for the hula-twist photoreaction and for photoinduced single-bond rotation is obtained. The influences of the surrounding medium and temperature are quantified and used to favor unusual photoreactions. Based on our findings molecular engineers will be able to implement photo control of complex molecular motions more consciously.

## Abstract (web summary)

Photoisomerization mechanisms govern important (bio)catalytic reactions and lie at the core of many functional materials. Here, the authors report a molecular setup that allows for the direct and separate observation of three fundamental photoreactions, namely the hula twist, single-bond rotation, as well as double-bond isomerization.

## Introduction

Light-induced motions of molecules play a fundamental role for sensing and responsiveness in nature and have become similarly important in the construction of artificial nanoscale machinery^1^, molecular motors^2–6^, smart materials^7–11^, or photopharmacophores^12,13^. In crucial cases however, the geometrical changes induced by light irradiation are not known and even the most fundamental photoreaction mechanisms are still under dispute^14–23^. This uncertainty not only hampers the conscious and rational design of synthetic systems with predictable responsive functions but at the same time prevents mechanistic and atomistic understanding of central photochemical processes in living systems.

**Fig. 1:**
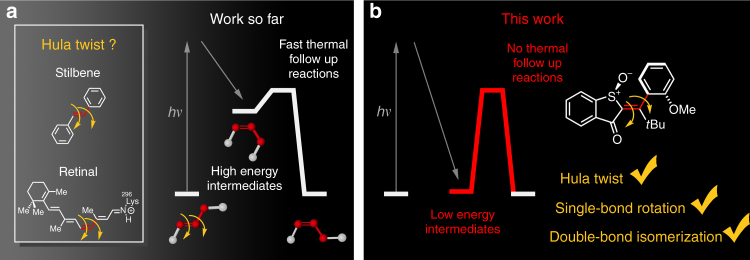
Complex light-induced motions in chromophores. **a** Proposed hula-twist (HT) motion for the photoisomerization of stilbene dyes or retinal in rhodopsin. Population of high-energy intermediates after the photoreaction leads to fast thermal decay and hampers a precise elucidation of their structural and electronic properties. **b** Chromophore **1** possesses only four possible, thermally highly stable, and geometrically distinct intermediate states and therefore enables unambiguous assignment of the photoproducts at ambient conditions. Three different geometrical changes could directly be observed as photoreactions: hula twist (HT), single-bond rotation (SBR), and double-bond isomerization (DBI)

One central problem concerns the photoisomerization of carbon–carbon double bonds with adjacent carbon–carbon single bonds (Fig. 1a)—a configuration that is found in the vast class of polyenes^24^, styrenes, and stilbene dye compounds^18,25–27^, and in the biological most relevant retinal^28,29^, *p*-coumaric acid^16,30,31^, previtamine D_3_^17,19,20^, or GFP chromophores^32,33^. For this bonding situation the photoreaction has essentially been described as either a sole double-bond isomerization (DBI)^17,34^, a combination of double bond and single-bond rotations i.e., the famous hula twist (HT) initially proposed by Liu^18,19,25,35–38^, or in extended conjugated systems as bipedal motions as proposed by Warshel^21,22^ and later experimentally shown by Saltiel^39–41^. Additionally, sole single-bond rotations (SBR) are also described as viable photoreactions^42–45^. The main problem to differentiate between these mechanisms is the short lived nature of primary products of the photoreaction, which are high-energy intermediates undergoing rapid thermal conversion to more stable structures in solution (Fig. 1a). Due to their fleeting character an unambiguous assignment of their molecular and electronic structure has not been possible so far, as time resolved methods^14–16,28,31,46,47^ or isolation of these intermediates under extremely cold^17,19,20^ or rigid-medium conditions^25,39,48,49^ provide still too little direct structural information. In rigid matrices (i.e., solvent glasses) and at very low temperatures intermediates have frequently been observed with absorption or fluorescence spectroscopy but the assignment of similar spectra to a particular isomeric structure is not straight forward. Consequently, Saltiel and co-workers have recently refuted earlier claims of HT evidences and identified the intermediate photoproducts as the result of simple DBIs^17,50,51^. The question whether a possible HT mechanism is an intrinsic property of the molecule itself^18^ or on the contrary is dictated mainly by outside restrictions^52,53^ (e.g., imposing volume conservation^16,54^ during the motion) is even less resolved.

**Fig. 2:**
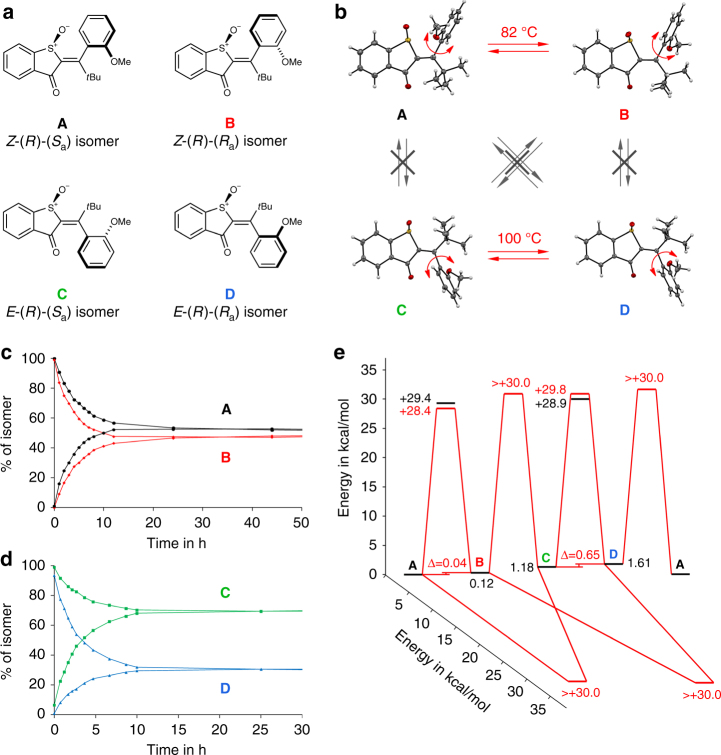
Ground state energy profile of HTI **1**. **a** Molecular geometries of the four stable isomeric states **A** to **D** of **1**. **b** Molecular structures of all four isomers of **1** obtained from crystal structural analysis and their slow thermal interconversions at high temperature (>80 °C). **c** Experimental first order kinetics for thermal interconversion of atropisomers **A** and **B** (82 °C) in (CDCl_2_)_2_ solution. **d** Experimental first order kinetics for thermal interconversion of atropisomers **C** and **D** (100 °C) in (CDCl_2_)_2_ solution. No thermal DBI occurs at elevated temperatures. The interconversion was quantified using ^1^H NMR spectroscopy. **e** Ground-state energy profile for **1**. Thermal conversion between the four different isomers at ambient temperature is prevented by very high kinetic barriers. Values in black are derived from quantum chemical calculations (B3LYP/6-311 G(d,p), PCM(DCM)), values in red were determined experimentally

To overcome this dilemma we have designed a geometrically strongly restricted asymmetric molecule **1** (Fig. 1b), which can assume only four stable diastereomeric states in the ground state (Fig. 2a). Different to the systems studied so far the barriers for thermal interconversion between the intermediate states are very high, which effectively decouples geometry changes during the photoreaction from thermal processes. With this molecular setup it is possible to track the formed photoproducts at ambient temperatures in solution directly and without interference of thermal follow-up reactions. After studying the photochemistry of **1** we provide direct experimental evidence for the presence of photoinduced HT, SBR, as well as the well-known sole DBI products. We are now able to isolate all these different photoproducts at ambient conditions and characterize them with high resolution techniques (NMR spectroscopy and crystal structural analysis). At the same time we are able to conveniently study the influences of polarity and rigidity of the surrounding medium, as well as temperature and give insights into how they affect the outcome of the photoreactions. Our findings give quantitative (photoquantum yields *ϕ* for each process) insights into the nature of photoinduced intramolecular motions, establish the exact influences of the surrounding medium, and clarify that light-induced HT, as well as SBR are viable and prominent photoreactions. We further give prospects of how these complex motions can be used for advanced photoresponsive tools and molecular machinery.

## Results

### Ground state energy profile of 1

Photoresponsive compound **1** is based on the hemithioindigo (HTI) chromophore^55–57^ and was synthesized according to a protocol previously developed in our group^58^. HTI **1** is rendered asymmetric via the introduction of a sulfoxide stereocenter and an adjacent chiral axis. This molecule can therefore exist in four geometrically and energetically different ground states, which we term **A**, **B**, **C**, and **D**. The stereo assignments of these states are given in Fig. 2a. We have obtained exact molecular structures from the crystalline state (Fig. 2b) for each isomer **A**, **B**, **C**, and **D** and could therefore unambiguously assign the corresponding high-resolution ^1^H NMR solution spectra to a particular geometry in each case (see Supplementary Figs. 1–4). **A** and **B** share the same *Z* configuration of the double bond but possess opposite axial chirality. Likewise **C** and **D** are atropisomers of each other in the *E* isomeric state. The atropisomers are thermally very stable, which is manifested in their extremely long solution half-lives of at least 1.7 years at 27 °C (see Fig. 2c, d for the kinetic measurements of their interconversion at >80 °C, see also Supplementary Figs. 5–8 and Supplementary Table 1). The thermal double bond rotation was not observed for any of the four isomers even after prolonged heating at 100 °C for 25 h, which gives a lower limit for the corresponding barrier to be at least 30 kcal/mol high (Fig. 2e, see also Supplementary Fig. 9 and Supplementary Table 1).

### Photochemistry of 1

**Fig. 3:**
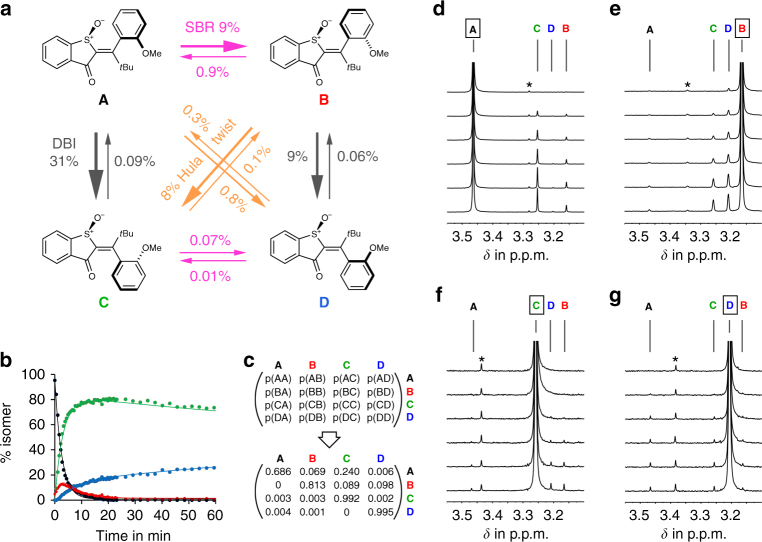
Photochemistry of **1** in benzene-*d*_*6*_ solution. **a** Individual photoconversions between the four isomers of **1** in benzene-*d*_*6*_ under 405 nm irradiation experimentally determined by quantum yield measurements. **b** Fitting of experimental data obtained at 27 °C in benzene-*d*_6_ (dots) to a global kinetic model (lines) for the photoconversion of individual isomers starting from pure **A**. **c** Markov matrix describing the phototransition probability between individual isomers (starting isomer to product isomer) within one minute of irradiation in benzene-*d*_6_. Numeric values were obtained from the best global fit to the experimental data. The ratios between off-diagonal elements mirror the ratios of the measured quantum yields for individual phototransitions. The diagonal elements describe the percentage of remaining starting isomer after one minute of irradiation. **d**–**g** Indicative sections of ^1^H NMR spectra (400 MHz, 27 °C, benzene-*d*_6_) recorded during irradiation of each pure isomer **A** (**d**), **B** (**e**), **C** (**f**), and **D** (**g**) at 23 °C. Different photoproducts are generated at different rates. * ^13^C carbon coupling satellites

The established high barriers for thermal interconversion render the individual isomers **A**, **B**, **C**, and **D** completely stable at ambient temperatures. It is therefore possible to study their photochemistry individually and without the complication of an intervening fast thermal decay after the photoreaction (for molar absorption coefficients see Supplementary Figs. 10–13). To this end we have irradiated solutions of either pure **A**, **B**, **C**, or **D** in benzene-*d*_*6*_ at 23 °C while counting the number of photons that led to photoproducts. This experiment established quantum yields (*ϕ*) for every photoconversion individually (Fig. 3a, see also Supplementary Figs. 14–18 and Supplementary Table 2). In a second independent experiment, the changes in isomer composition of the solution were measured for different time points during prolonged irradiation at 27 °C. To disentangle the different photo processes at later stages of the irradiation wholesome simulations of the different photokinetics of **A**, **B**, **C**, and **D** were conducted (Fig. 3b, c, see also Supplementary Figs. 19–43). As we kept the irradiation conditions constant in all experiments these latter kinetic measurements could be used to obtain relative probabilities for the individual photo-transformations (see Supplementary Fig. 44 and Supplementary Table 2). Fitting the experimental data to a Markov-matrix derived kinetic model provided excellent agreement between measured quantum yield ratios and the kinetic experiments. For a further assignment of the photoproducts’ identities the starting points of the photoconversions were examined more closely (Fig. 3d–g). At this time point the concentration of the different photoproducts is low enough to prevent them from light absorption in the presence of a far higher concentration of the starting material. As the photoconversions progress photoproducts are accumulating and enter their respective photoequilibria as well. All three approaches, determination of quantum yields for individual phototransformations in benzene, fitting kinetic data of the whole photoconversion process to a kinetic model, and independent identification of the photoproducts at early stages of the irradiation provided the same conclusions.

As shown in Fig. 3 each isomer **A**, **B**, **C**, or **D** converts directly into more than one photoproduct after irradiation. The efficiencies of these transformations differ dramatically but it is clearly evident that very different types of motions are generated by light irradiation of different isomers.

Irradiation of **A** at 405 nm in benzene-*d*_6_ at 27 °C leads to three photoproducts with **B** and **C** being the main products formed in *ϕ* = 9% and 31%, respectively (Fig. 3a). Isomer **D** is formed with only 0.8% quantum yield and thus represents a minor channel of the photoreaction. Irradiation of **B** leads to **C** (*ϕ* = 8%) and **D** (*ϕ* = 9%) in almost the same efficiencies while formation of **A** presents just a minor channel (*ϕ* = 0.9%). The photoreactions of **C** and **D** are much less effective in comparison but are also branching in each case. Irradiation of **C** leads to inefficient transformation to **A** (*ϕ* = 0.09%), **B** (*ϕ* = 0.1%), and **D** (*ϕ* = 0.07%). Photoconversion of **D** leads to slow population of **A** (*ϕ* = 0.3%), **B** (*ϕ* = 0.06%), and **C** (*ϕ* = 0.01%).

With these measurements we could unambiguously show that all three possibilities of light-induced bond rotations are actually realized in **1**. The simple DBI is most prominent in the light-induced transition of **A** to **C**. Highly efficient is also the hitherto elusive HT, which couples double bond and SBRs, as seen by the transformation of **B** to **C**. Interestingly the HT for **B** is almost as efficient as the DBI from **B** to **D**. For isomer **D** the HT leading to **A** is actually the most prominent pathway. A sole SBR could also directly be demonstrated as dominant photoreaction—exemplified by the prolific photoconversion of atropisomer **A** into **B**. With the term HT we emphasize the causal connection of double and SBRs occurring after photoexcitation. This does however not mean that both motions must occur in a concerted way as originally proposed by Liu and Hammond^52^. A sequential motion as proposed by Saltiel^52^ is also a possibility as discussed below in the Discussion section.

### Medium and temperature effects on the photoreactions of 1

**Fig. 4:**
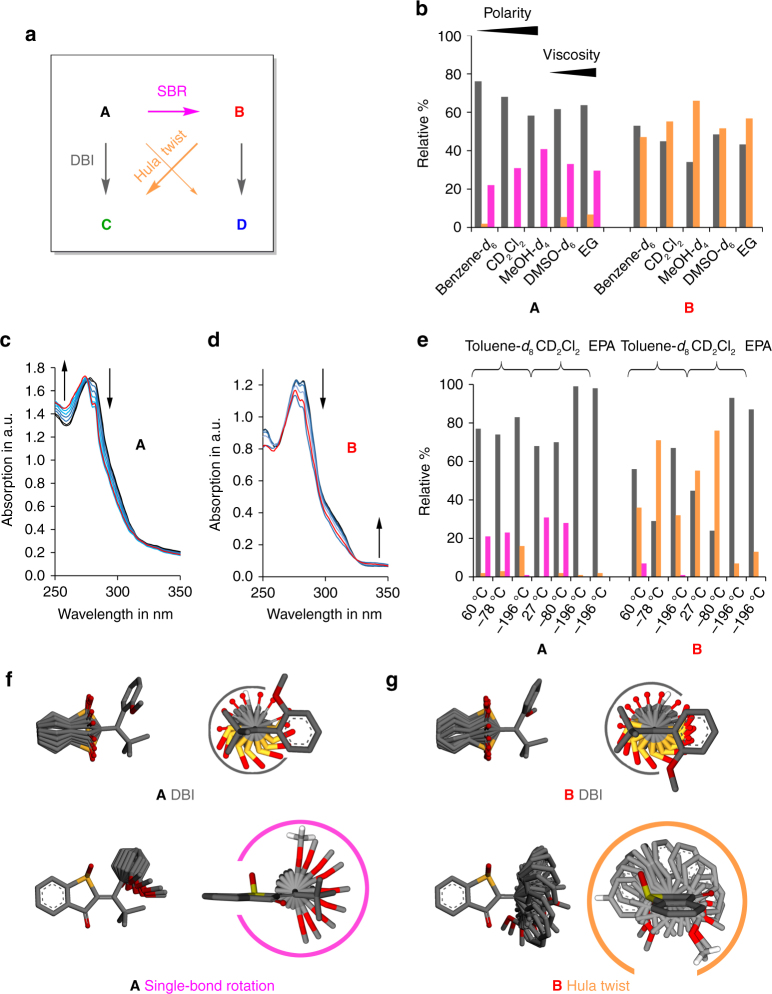
Photoreactions of **A** and **B** under different conditions. **a** The different photo processes are color coded: SBR in purple, DBI in gray, and HT in orange. **b** Dependence of the photoinduced motions on solvent polarity and viscosity. The relative efficiencies of different processes are derived from a Markov-matrix analysis of the corresponding ^1^H NMR data. **c** Ultraviolet/vis absorption changes (arrows) observed during irradiation of **A** at −183 °C within an EPA matrix. No significant spectral changes are observed after warming the sample to −80 °C and recooling to −183 °C (red spectrum). **d** Ultraviolet/vis absorption changes observed during irradiation of **B** at −183 °C within an EPA matrix. No significant spectral changes are observed after warming the sample to −80 °C and recooling to −183 °C (red spectrum). **e** Dependence of the photoinduced motions on temperature and rigidity of the medium. The relative efficiencies of different processes are derived from a Markov-matrix analysis of the corresponding ^1^H NMR data. **f** Approximated volume changes of **A** during DBI (top) and SBR (bottom). Two different views are shown. The required volumes for individual motions are emphasized by color coded circles, perspectives are at the same scale. **g** Approximated volume changes of **B** during DBI (top) and HT motion (bottom)

After quantifying the different photoconversion pathways between **A**, **B**, **C**, and **D** in benzene-*d*_6_ solution the influences of the surrounding medium were of primary interest. With the experiments described above it became already clear that even in a nonrestrictive solution environment there is an intrinsic propensity to undergo coupled motions as opposed to just simple DBI after photoexcitation of **1**. With the term coupled motions we again emphasize the causal connection of double and single-bond rotations occurring after photoexcitation without specific mechanistic implications. As different motions appear concomitantly after photoexcitation of **1** it is of utmost importance to understand the influence of the surrounding environment on a particular motion. Only then these motions can be used in a conscious way for advanced functions such as complex light driven molecular machinery or smart nanosystems. In the following we present possible ways in which such selectivity for particular motions via the surrounding medium can be achieved. We focus on the light-induced motions of **A** and **B** (Fig. 4a) since they display the most efficient photoreactions and experimental errors are therefore very small.

Photoirradiation of **A** in benzene-*d*_6_ solution results mainly in DBI to **C** and SBR to **B**. The relative ratio of these processes is roughly 80%:20% (Fig. 4a). Increasing the polarity of the solvent changes this ratio to a value of 60%:40% in MeOH-*d*_4_, thus enhancing the SBR considerably (Fig. 4b). If the polarity of the solvent is kept constant but the viscosity is increased drastically (MeOH-*d*_4_ to ethylene glycol (EG)) the DBI is becoming more pronounced again and a decrease of the SBR is observed. The inefficient HT motion from **A** to **D** is increased slightly at high viscosity conditions (see also Supplementary Figs. 19–44).

The light-induced motions of **B** are also very sensitive to the solvent nature. In benzene-*d*_6_ the SBR and HT motions are similarly efficient. An increase of solvent polarity induces the most pronounced changes and clearly favors the HT motion. In the most polar MeOH-*d*_4_ a 70%:30% ratio is observed for the relative propensities to undergo HT versus DBI. Viscosity changes produce smaller, yet discernible trends showing an increase in the propensity for simple DBI with increasing viscosity (Fig. 4b, see also Supplementary Figs. 19–44 and Supplementary Table 2).

Additionally we have tested the influence of temperature in liquid and in solid media on the different photoinduced motions of **A** and **B** (Fig. 4c–e, see also Supplementary Figs. 45–53 and Supplementary Table 3). Irradiation of **A** in toluene-*d*_8_ and CD_2_Cl_2_ showed no significant changes in the photoreactions at elevated (60 °C in toluene-*d*_8_), ambient (20 or 27 °C), and low (−78 or −80 °C) temperatures (Fig. 4e, see also Supplementary Figs. 49 and 50). The situation is quite different for **B** for which temperature effects are very pronounced. At 60 °C in toluene-*d*_8_ solution DBI is clearly the favored photoreaction (56% versus 36% HT) and also 7% of the SBR is observed. At 20 °C DBI is still favored with 54% but the HT motion is now more pronounced with 43% at the expense of the SBR. This selectivity changes dramatically at −78 °C, where the HT photoreaction is now strongly favored with 71% versus 29% DBI. In CD_2_Cl_2_ the changes are similarly dramatic. At 27 °C HT is slightly favored with 55% over DBI with 45%. At −80 °C the HT is strongly favored with 76% (see also Supplementary Figs. 45–46).

When freezing toluene-*d*_8_ or CD_2_Cl_2_ solutions of **A** and **B** to ice at −196 °C irradiation still leads to photoreactions. Irradiation of **A** in toluene-*d*_8_ ice results in strongly preferred formation of the DBI product **C** (83%) and almost complete suppression of the SBR product **B** (1%). Instead the HT pathway clearly gains in efficiency with 16%. In CD_2_Cl_2_ the trend is even more pronounced with almost exclusive selectivity for the DBI product **C** (99%). Irradiation of **B** in toluene-*d*_8_ ice also leads to preference of the DBI, however in this case the HT is still very pronounced with 32%. The situation is different in CD_2_Cl_2_ where almost exclusive DBI is observed. Therefore, in CD_2_Cl_2_ it is possible to strongly favor either HT (76% of all photoreactions) at low temperature in liquid solution or DBI (93%) at even lower temperature in solvent glass, which is a remarkable control over photoreactions (see also Supplementary Figs. 45–53). Photoisomerization experiments were also conducted during irradiation with different wavelengths. Only marginal changes in photoproduct composition could be observed by varying the wavelength from 305 to 405 nm (see also Supplementary Figs. 54–55 and Supplementary Table 4).

We also conducted low temperature irradiation experiments of **A** and **B** in diethyl ether/*iso*-pentane/ethanol (EPA) glass at −183 °C and followed the photoreactions with ultraviolet/visible absorption spectroscopy (see also Supplementary Figs. 13, 56–59). Isomers **C** and **D** were found not to undergo photoreactions under these conditions (i.e., within 2 h of irradiation). Irradiation of **A** in EPA glass proceeds with clear isosbestic points leading to the absorption spectrum of **C**. After 30 min of 405 nm illumination the sample was allowed to warm to −80 °C and then recooled to −183 °C. Only marginal spectral changes are observed after the warming and cooling procedure indicating no thermally labile intermediates being formed during irradiation in the EPA matrix (Fig. 4c, see also Supplementary Fig. 56). The isomer composition obtained from irradiation of **A** in the EPA matrix at low temperature was determined directly afterwards by NMR spectroscopy and showed only formation of **C**. An independent Markov analysis of the isomer composition changes occurring during photoirradiation of **A** in EPA glass at −196 °C showed almost exclusive formation of the DBI product **C**. Irradiation of **B** in the EPA glass at −183 °C leads to clear isosbestic points during the irradiation, and no significant changes upon warming and re-cooling (Fig. 4d, see also Supplementary Fig. 57). Again, the isomer composition obtained from irradiation of **B** in the EPA matrix at −183 °C was determined directly afterwards by NMR spectroscopy and showed formation of **C** and **D** in almost equal amounts. Photoirradiation of **B** in EPA glass at −196 °C was scrutinized by Markov analysis revealing almost exclusive formation of the DBI product and about 13% of the HT product at these conditions. There seems to be a significant temperature effect also in rigid matrix surroundings. Overall, at −196 °C the propensities for a particular photoreaction in EPA are similar to the ones found in CD_2_Cl_2_ ice, i.e., strongly favoring DBI. For comparison we tested also the corresponding photoreactions of **A** and **B** in liquid EPA solution at 22 °C, as well as −80 °C. The same trends are seen as for the other liquid solvents: no temperature effects on the photoreactions of **A** but significant effects for **B**. For the latter at ambient temperature the HT is slightly favored and becomes strongly favored (82%) at lower temperature in solution. For comparison of all relative quantum yields at different temperature in different solvents see Fig. 4e and Supplementary Fig. 60.

## Discussion

From our experiments it becomes clear that more polar solvents and the capacity for hydrogen bonding favor the unusual SBR and coupled HT motions against the well-known simple DBI. Decreasing the temperature in solution leads to no significant changes for the SBR compared to DBI in **A**. However, for **B** dramatic changes are observed when lowering the temperature. A significant increase of the HT product is detected in this case regardless of the solvent, but most dramatically in CD_2_Cl_2_ and EPA where 76% and 82% HT is observed at −80 and −78 °C, respectively. Irradiation in low temperature solvent ices or glasses strongly emphasizes the trends observed when increasing solvent viscosities. In these rigid media almost exclusive DBI is observed for **A** and likewise also a very strong preference for DBI of **B**. In the latter case HT is nevertheless still present to appreciable degree. The decrease of the SBR for **A** in more viscous solvents can be explained by the more space-demanding nature of this motion compared to the DBI in **1** (Fig. 4f). It was previously suggested that external volume restrictions and high viscosity evoke the HT motion, but we clearly did not observe such an influence in our experiments with **B**. On the contrary, we found the DBI is actually strongly favored at higher viscosity and rigidity of the surrounding medium. Our explanation for these seemingly contradictory findings is rooted in the special setup of HTI **1**. When tracing the geometry changes of DBI versus HT motion in a simple bond-rotation model (as we do not know the exact light-induced motions the simple bond-rotation model should however be regarded as just a crude visualization) the latter seems to be even more space-demanding than the competing DBI (Fig. 4g). Because in rigid glasses viscosity effects are strongly amplified it is possible to almost exclusively favor DBI for **A** and **B** e.g., in CD_2_Cl_2_ ice.

The clear influence of temperature, polarity, hydrogen bonding capacity, as well as viscosity/rigidity of the environment hints at main factors governing complex and unusual light-induced motions in molecules. With the herein presented molecular setup these different influences could be quantified directly and allowed us to either strongly favor the HT motion or the simple DBI against competing photoreactions. Likewise the propensity to undergo SBR could be increased by simple changes in the nature of the solvent at ambient temperatures.

From the data obtained in this work some indications with regard to the mechanism of HT photoproduct formation could be obtained. First, the energy barriers between all four isomeric states **A**–**D** are very high and thermal equilibration occurs both, slowly even at temperatures >80 °C and selectively between **A** and **B** and separated between **C** and **D**. The observed HT photoproducts therefore cannot be formed by first DBI in the excited state, relaxation to the ground state minima and subsequent thermal SBRs. A high-energy ground state intermediate that is populated after the photoreaction and from which a branching towards HT and DBI products occurs is also highly unlikely. The strongest evidence against the presence of such an intermediate is found in the temperature dependence of the photoreaction selectivity in toluene-*d*_8_ solution. At high and ambient temperatures a clear selectivity favoring DBI is observed. This selectivity is turned upside down at low temperatures where HT products are formed with high preference. Such temperature sensitivity cannot be the result of thermal branching from a common intermediate state, because in such a case the initial trend should be augmented at lower temperature where energy-barrier differences are more important. The only other option for a common ground-state intermediate process—thermodynamic product formation at high and ambient temperatures and kinetic product formation at low temperatures—can also be ruled out, since at ambient temperature there is no thermal equilibrium between the isomers **A**–**D** established.

The absence of any discernible fluorescence in solution also disfavors an adiabatic mechanism. To distinguish between the remaining possibilities, a mechanism involving hot-ground states or a diabatic photoreaction requires more detailed studies and time-resolved measurements, which we will pursue in the near future. To speculate, the observed strong temperature dependence of the photoreactions of **B** could be interpreted in terms of a hot-ground state mechanism^59^. On the other hand, a diabatic process where the product distribution is determined earlier by a conical intersection^60–62^ is also possible. In line with this interpretation is the observed formation of all three possible photoproducts (although in varying efficiencies) after irradiation of **A**, **B**, **C**, and **D**, which is indicative for a kinked conical intersection^60,61,63,64^. As solvent polarity increase favors the HT product in our case, a polar ionic conical intersection leading to sole DBI (usually observed in permanently charged systems^62,65^) is likely to be absent in our system. Here the found solvent dependence could be explained by the involvement of an ionic charge-transfer state perturbing the photochemically active state and its decay to the ground state^65,66^. Other options involving further reaction coordinates, and thus more complex conical intersections^67^, or the existence of an extended crossing space are also possible^68^. The insensitivity of the photoreactions of **A** towards temperature disfavors a hot ground state in this case and instead hint at a photoreaction directly via conical intersections.

In summary, hitherto ephemeral light-induced motions were directly and unambiguously proven to exist. The exact influences of the surrounding medium and temperature were quantified directly and enabled us to favor unusual photoreactions and complex motions against the long known simple DBI. Just by changing temperature and rigidity of the outside medium we were able to favor HT photoproducts with up to 82% or the simple DBI products by up to 99%. Using the herein presented molecular setup it is now possible to rationally design HTI photoswitches with unusual motions, complex molecular machines, or responsive and functional materials and nanosystems. Likewise molecular engineers will be able to test for the presence of hula-twist photoreactions in other classes of chromophores by applying the herein established design principles: reduction of conformational space, introduction of asymmetry into the molecule via stable stereogenic centers in conjunction with chiral axes, and inhibition of thermal single bond rotation by increased sterical hindrance. It did not escape our attention that the combination of all the different photoinduced motions for **A**–**1** to **D**–**1** under constant illumination are very likely to lead to a netto-directional motion around a virtual axis. Therefore, HTI **1** could actually constitute a completely different type of light-driven molecular motor with unique rotation mechanism. Our future efforts are inter alia aimed in this exciting new direction.

## Methods

### General experimental

The synthesis and spectroscopic characterization of **A**–**1**, **B**–**1**, **C**–**1**, and **D**–**1** is given in the Supplementary Methods. Reagents and solvents were obtained from abcr, Acros, Fluka, Merck, Sigma-Aldrich, or TCI in the qualities puriss., p.a., or purum and used as recieved. Technical solvents were distilled before use for column chromatography and extraction on a rotary evaporator (Heidolph Hei-VAP Value, vacuubrand CVC 3000). Reactions were monitored on Merck Silica 60 F254 TLC plates. Detection was done by irradiation with UV light (254 nm or 366 nm). Column chromatography was performed with silica gel 60 (Merck, particle size 0.063–0.200 mm) and distilled technical solvents. ^1^H NMR and ^13^C NMR spectra were measured on a Varian Mercury 200 VX, Varian 300, Inova 400, Varian 600 NMR, or Bruker Avance III HD 800 MHz spectrometer at 23 °C. Chemical shifts (*δ*) are given relative to tetramethylsilane as external standard. Residual solvent signals in the ^1^H and ^13^C NMR spectra were used as internal reference. Deuterated solvents were obtained from Cambridge Isotope Laboratories or Eurisotop and used without further purification. For ^1^H NMR: CDCl_3_ = 7.26 p.p.m., CD_2_Cl_2_ = 5.32 p.p.m., benzene-*d*_6_ = 7.16 p.p.m., toluene-*d*_8_ = 2.08 p.p.m., (CDCl_2_)_2_ = 6.00 p.p.m., cyclohexane-*d*_12_ = 1.38 p.p.m., (CD_3_)_2_SO = 2.50 p.p.m., THF-*d*_8_ = 1.72, 3.58 p.p.m., MeOH-*d*_4_ = 3.31 p.p.m. For ^13^C NMR: CDCl_3_ = 77.16 p.p.m., CD_2_Cl_2_ = 53.84 p.p.m., benzene-*d*_6_ = 128.06 p.p.m., toluene-*d*_8_ = 20.43, cyclohexane-*d*_12_ = 26.43 p.p.m., THF-*d*_8_ = 67.57, 23.37 p.p.m., MeOH-*d*_4_ = 49.00 p.p.m. The resonance multiplicity is indicated as s (singlet), d (doublet), t (triplet), q (quartet), and m (multiplet). The chemical shifts are given in parts per million (p.p.m.) on the delta scale (*δ*). The coupling constant values (J) are given in hertz (Hz). Electron Impact (EI) mass spectra were measured on a Finnigan MAT95Q or on a Finnigan MAT90 mass spectrometer. Electronspray ionization (ESI) mass spectra were measured on a Thermo Finnigan LTQ-FT. The most important signals are reported in *m*/*z* units with M as the molecular ion. Elemental analysis were performed in the micro analytical laboratory of the LMU department of chemistry on an Elementar Vario EL apparatus. Infrared spectra were recorded on a Perkin Elmer Spectrum BX-FT-IR instrument equipped with a Smith DuraSamplIR II ATR-device. Transmittance values are qualitatively described by wavenumber (cm^−1^) as very strong (vs), strong (s), medium (m), and weak (w). UV/Vis spectra were measured on a Varian Cary 5000 spectrophotometer. The spectra were recorded in a quartz cuvette (1 cm). Solvents for spectroscopy were obtained from VWR and Merck. Absorption wavelength (*λ*) are reported in nm and the molar absorption coefficients (ε) in L mol^−1^ cm^−1^. Low temperature UV/vis spectra in EPA glass (diethylether/isopentane/ethanol 5:5:2) at 90 K (−183 °C) were measured on a Varian Cary® 50 spectrophotometer with an Oxford DN 1704 optical cryostat controlled by an Oxford ITC 4 device. Low temperatures were reached by cooling slowly with liquid nitrogen. The spectra were recorded in a quartz cuvette (1 cm). Solvents for spectroscopy were obtained from VWR, Merck and Sigma Aldrich and were dried, degassed and filtrated prior use. For irradiation studies a Mightex FCS-0405-200 LED (405 nm) was used as light source. Absorption wavelength (*λ*) are reported in nm and the molar absorption coefficients (ε) in L·mol^−1^·cm^−1^. Melting points (M.p.) were measured on a Stuart SMP10 melting point apparatus in open capillaries and are not corrected.

### Photoisomerization experiments

Continuous irradiations of the solutions were conducted in NMR tubes in different solvents (CD_2_Cl_2_, (CDCl_2_)_2__,_ benzene-*d*_6,_ toluene-*d*_8_, MeOH-*d*_4,_ DMSO-*d*_6_, EPA, EG). Irradiations were conducted using LEDs from Roithner Lasertechnik GmbH (305 nm, 365 nm, 405 nm). For low temperature studies a Mightex FCS-0405-200 LED (405 nm) was used as light source and the light beam was guided by a fiber-optic cable (0.39 NA, one SMA, one blank end) and pointed directly into the NMR tube during NMR measurements. For quantum yield measurements see Supplementary Methods.

### Data availability

All data that support the findings of this study are available from the corresponding author upon reasonable request. The X-ray crystallographic coordinates for the structures **A**–**1** to **D**–**1** reported in this study have been deposited at the Cambridge Crystallographic Data Centre (CCDC), under CCDC numbers 1586011 (**A**–**1**), 1586012 (**B**–**1**), 1586013 (**C**–**1**), 1586014 (**D**–**1**). These data can be obtained free of charge from the Cambridge Crystallographic Data Centre via www.ccdc.cam.ac.uk/data_request/cif.

## Electronic supplementary material

Supplementary Information

## References

[1] Erbas-Cakmak S, Leigh DA, McTernan CT, Nussbaumer AL (2015). Artificial molecular machines. Chem. Rev..

[2] Koumura N, Zijlstra RWJ, van Delden RA, Feringa BL (1999). Light-driven monodirectional molecular rotor. Nature.

[3] Greb L, Lehn JM (2014). Light-driven molecular motors: imines as four-step or two-step unidirectional rotors. J. Am. Chem. Soc..

[4] Hernandez JV, Kay ER, Leigh DA (2004). A reversible synthetic rotary molecular motor. Science.

[5] Guentner M (2015). Sunlight-powered kHz rotation of a hemithioindigo-based molecular motor. Nat. Commun..

[6] Huber LA (2017). Direct observation of hemithioindigo-motor unidirectionality. Angew. Chem. Int. Ed..

[7] Foy JT (2017). Dual-light control of nanomachines that integrate motor and modulator subunits. Nat. Nanotechnol..

[8] Li H (2013). Relative unidirectional translation in an artificial molecular assembly fueled by light. J. Am. Chem. Soc..

[9] Kundu PK (2015). Light-controlled self-assembly of non-photoresponsive nanoparticles. Nat. Chem..

[10] Fuhrmann A (2016). Conditional repair by locally switching the thermal healing capability of dynamic covalent polymers with light. Nat. Commun..

[11] Iamsaard S (2014). Conversion of light into macroscopic helical motion. Nat. Chem..

[12] Frank JA (2016). Photoswitchable diacylglycerols enable optical control of protein kinase C. Nat. Chem. Biol..

[13] Velema WA, Szymanski W, Feringa BL (2014). Photopharmacology: beyond proof of principle. J. Am. Chem. Soc..

[14] Jung YO (2014). Reply to ‘contradictions in X-ray structures of intermediates in the photocycle of photoactive yellow protein’. Nat. Chem..

[15] Kaila VRI, Schotte F, Cho HS, Hummer G, Anfinrud PA (2014). Contradictions in X-ray structures of intermediates in the photocycle of photoactive yellow protein. Nat. Chem..

[16] Jung YO (2013). Volume-conserving trans-cis isomerization pathways in photoactive yellow protein visualized by picosecond X-ray crystallography. Nat. Chem..

[17] Redwood C, Bayda M, Saltiel J (2013). Photoisomerization of pre- and provitamin D3 in EPA at 77 K: one-bond-twist, not hula-twist. J. Phys. Chem. Lett..

[18] Fuß W, Kosmidis C, Schmid WE, Trushin SA (2004). The photochemical cis-trans isomerization of free stilbene molecules follows a hula-twist pathway. Angew. Chem. Int. Ed..

[19] Müller AM, Lochbrunner S, Schmid WE, Fuß W (1998). Low-temperature photochemistry of previtamin d: a hula-twist isomerization of a triene. Angew. Chem. Int. Ed..

[20] Maessen PA, Jacobs HJC, Cornelisse J, Havinga E (1983). Photochemistry of previtamin D3 at 92 K; formation of an unstable tachysterol3 rotamer. Angew. Chem. Int. Ed..

[21] Warshel A (1976). Bicycle-pedal model for the first step in the vision process. Nature.

[22] Schapiro I, Weingart O, Buss V (2009). Bicycle-pedal isomerization in a rhodopsin chromophore model. J. Am. Chem. Soc..

[23] Liu RSH, Hammond GS (2003). Photochemical reactivity of polyenes: from dienes to rhodopsin, from microseconds to femtoseconds. Photochem. Photobiol. Sci..

[24] Ramamurthy V, Liu RSH (1976). Photochemistry of polyenes. IX. Excitation, relaxation, and deactivation of dienes, trienes, and higher polyenes in the vitamin A series in the sensitized isomerization reaction. J. Am. Chem. Soc..

[25] Imamoto Y (2003). Photoisomerization by Hula Twist: 2,2’-dimethylstilbene and a ring-fused analogue. Angew. Chem., Int. Ed..

[26] Waldeck DH (1993). Photoisomerization dynamics of stilbenes in polar solvents. J. Mol. Liq..

[27] Görner, H. & Kuhn, H. J. In *Advances in Photochemistry* (eds Neckers, D. C. et al.) Ch 1 (John Wiley & Sons, Inc., 2007).

[28] Kukura P, McCamant DW, Yoon S, Wandschneider DB, Mathies RA (2005). Structural observation of the primary isomerization in vision with femtosecond-stimulated Raman. Science.

[29] Ernst OP (2014). Microbial and animal rhodopsins: structures, functions, and molecular mechanisms. Chem. Rev..

[30] Wei L, Wang H, Chen X, Fang W, Wang H (2014). A comprehensive study of isomerization and protonation reactions in the photocycle of the photoactive yellow protein. Phys. Chem. Chem. Phys..

[31] Pande K (2016). Femtosecond structural dynamics drives the trans/cis isomerization in photoactive yellow protein. Science.

[32] Zhang Q, Chen X, Cui G, Fang WH, Thiel W (2014). Concerted asynchronous hula-twist photoisomerization in the S65T/H148D mutant of green fluorescent protein. Angew. Chem. Int. Ed..

[33] Andresen M (2005). Structure and mechanism of the reversible photoswitch of a fluorescent protein. Proc. Natl Acad. Sci. USA.

[34] Saltiel J, Megarity ED, Kneipp KG (1966). The mechanism of direct *cis*-*trans* photoisomerization of the stilbenes. J. Am. Chem. Soc..

[35] Liu RS, Asato AE (1985). The primary process of vision and the structure of bathorhodopsin: a mechanism for photoisomerization of polyenes. Proc. Natl Acad. Sci. USA.

[36] Liu RS, Hammond GS (2001). Examples of hula-twist in photochemical cis- trans isomerization. Chem. Eur. J..

[37] Fuß W (2012). Hula-twist cis–trans isomerization: the role of internal forces and the origin of regioselectivity. J. Photochem. Photobiol., A.

[38] Röhrig UF, Guidoni L, Laio A, Frank I, Rothlisberger U (2004). A molecular spring for vision. J. Am. Chem. Soc..

[39] Saltiel J, Bremer MA, Laohhasurayotin S, Krishna TS (2008). Photoisomerization of cis,cis- and cis,trans-1,4-Di-o-tolyl-1,3-butadiene in glassy media at 77 K: one-bond-twist and bicycle-pedal mechanisms. Angew. Chem. Int. Ed..

[40] Saltiel J (2009). Photoisomerization of all-cis-1,6-diphenyl-1,3,5-hexatriene in the solid state and in solution: a simultaneous three-bond twist process. Angew. Chem. Int. Ed..

[41] Saltiel, J., Krishna, T. S., Turek, A. M. & Clark, R. J. Photoisomerization of cis,cis-1,4-diphenyl-1,3-butadiene in glassy media at 77 K: the bicycle-pedal mechanism. *Chem. Commun*. **0**, 1506–1508 (2006). 10.1039/b516319f 16575442

[42] Squillacote ME, Sheridan RS, Chapman OL, Anet FAL (1979). Planar s-cis-1,3-butadiene. J. Am. Chem. Soc..

[43] Werst DW, Londo WF, Smith JL, Barbara PF (1985). The excited-state torsional potentials of substituted 9-phenylanthracenes. Chem. Phys. Lett..

[44] Brouwer AM, Jacobs HJC (1995). Photochemistry of 2,5-dimethyl-1,3,5-hexatrienes in argon matrices. Formation of isomers and rotamers. Recl. Trav. Chim. Pays-Bas.

[45] Furukawa Y, Takeuchi H, Harada I, Tasumi M (1983). Matrix-isolation infrared and ultraviolet spectroscopic studies of less stable conformers of 1,3,5-hexatriene. J. Mol. Struct..

[46] Schoenlein R, Peteanu L, Mathies R, Shank C (1991). The first step in vision: femtosecond isomerization of rhodopsin. Science.

[47] van Wilderen LJ (2006). Ultrafast infrared spectroscopy reveals a key step for successful entry into the photocycle for photoactive yellow protein. Proc. Natl Acad. Sci. USA.

[48] Yang LY, Liu RSH (2007). Mechanism of photoisomerization of 1-naphthyl-2-phenylethylenes in organic glasses. Photochem. Photobiol..

[49] Liu RSH, Yang LY, Liu J (2007). Mechanisms of photoisomerization of polyenes in confined media: from organic glasses to protein binding cavities. Photochem. Photobiol..

[50] Bayda M, Redwood CE, Gupta S, Dmitrenko O, Saltiel J (2017). Lumisterol to tachysterol photoisomerization in EPA glass at 77 K. A. J. Phys. Chem. A.

[51] Redwood C (2015). Photoisomerization of cis-1,2-di(1-Methyl-2-naphthyl)ethene at 77 K in glassy media. Photochem. Photobiol..

[52] Liu RSH, Hammond GS (2000). The case of medium-dependent dual mechanisms for photoisomerization: one-bond-flip and hula-twist. Proc. Natl Acad. Sci. USA.

[53] Liu RSH, Hammond GS (2005). Reflection on medium effects on photochemical reactivity. Acc. Chem. Res..

[54] Balomenou I, Pistolis G (2009). Torsional photoisomerization proceeding adiabatically through a volume-conserving pathway in uninhibited fluid media. Chem. Eur. J..

[55] Wiedbrauk S, Dube H (2015). Hemithioindigo—an emerging photoswitch. Tetrahedron Lett..

[56] Kitzig S, Thilemann M, Cordes T, Rück-Braun K (2016). Light-switchable peptides with a hemithioindigo unit: peptide design, photochromism, and optical spectroscopy. Chemphyschem.

[57] Wiedbrauk S (2016). Twisted hemithioindigo photoswitches: solvent polarity determines the type of light-induced rotations. J. Am. Chem. Soc..

[58] Gerwien A, Reinhardt T, Mayer P, Dube H (2018). Synthesis of double-bond substituted hemithioindigo photoswitches. Org. Lett..

[59] Arruda BC, Sension RJ (2014). Ultrafast polyene dynamics: the ring opening of 1,3-cyclohexadiene derivatives. Phys. Chem. Chem. Phys..

[60] Robb, M. A., Garavelli, M., Olivucci, M. & Bernardi, F. In *Reviews of Computational Chemistry* (eds Lipkowitz, K. B. et al.) 87–146 (John Wiley & Sons, Inc., 2007).

[61] Olivucci, M. *Computational Photochemistry* 1st edn (Elsevier, 2005).

[62] Sampedro Ruiz D, Cembran A, Garavelli M, Olivucci M, Fuß W (2002). Structure of the conical intersections driving the cis–trans photoisomerization of conjugated molecules. Photochem. Photobiol..

[63] Levine BG, Martinez TJ (2007). Isomerization through conical intersections. Annu. Rev. Phys. Chem..

[64] Celani P (1995). Molecular “Trigger” for radiationless deactivation of photoexcited conjugated hydrocarbons. J. Am. Chem. Soc..

[65] Gonzalez-Luque R (2000). Computational evidence in favor of a two-state, two-mode model of the retinal chromophore photoisomerization. Proc. Natl Acad. Sci. USA.

[66] Cembran A, Bernardi F, Olivucci M, Garavelli M (2005). The retinal chromophore/chloride ion pair: Structure of the photoisomerization path and interplay of charge transfer and covalent states. Proc. Natl Acad. Sci. USA.

[67] Polli D (2010). Conical intersection dynamics of the primary photoisomerization event in vision. Nature.

[68] Conti I, Garavelli M, Orlandi G (2008). The different photoisomerization efficiency of azobenzene in the lowest nπ* and ππ* singlets: the role of a phantom state. J. Am. Chem. Soc..

