# Supplementary material for: Direct evidence for hula twist and single-bond rotation photoproducts
Source: Nat Commun. 2018 Jun 28;9:2510. doi: 10.1038/s41467-018-04928-9 (PMC6023863; doi:10.1038/s41467-018-04928-9)
Supplement: Supplementary file 1 — Supplementary Information [file 41467_2018_4928_MOESM1_ESM.pdf]

Supplementary Information

Direct Evidence for Hula Twist and Single-Bond Rotation Photoproducts

A. Gerwien et al.

Supplementary Methods

Synthesis

General experimental

Reagents and solvents were obtained from *abcr*, *Acros, Fluka, Merck, Sigma-Aldrich* or *TCI* in the qualities *puriss., p.a., or purum* and used as recieved. Technical solvents were distilled before use for column chromatography and extraction on a rotary evaporator (*Heidolph Hei-VAP Value*, *vacuubrand* CVC 3000). Reactions were monitored on *Merck* Silica 60 F254 TLC plates. Detection was done by irradiation with UV light (254 nm or 366 nm).

Column chromatography was performed with silica gel 60 (*Merck*, particle size 0.063- 0.200 mm) and distilled technical solvents.

^{1}H NMR and ^{13}C NMR spectra were measured on a *Varian Mercury 200 VX, Varian 300, Inova 400, Varian 600 NMR* or *Bruker Avance III HD 800 MHz* spectrometer at different temperatures. Chemical shifts (δ) are given relative to tetramethylsilane as external standard. Residual solvent signals in the ^{1}H and ^{13}C NMR spectra were used as internal reference. Deuterated solvents were obtained from *Cambridge Isotope Laboratories* or *Eurisotop* and used without further purification. ^{1}H NMR: $CDCl_3$ = 7.26 ppm, CD_2Cl_2 = 5.32 ppm, benzene-d_6 = 7.16 ppm, toluene-d_8 = 2.08 ppm, $(CDCl_2)_2$ = 6.00 ppm, cyclohexane-d_{12} = 1.38 ppm, $(CD_3)_2SO$ = 2.50 ppm, THF-d_8 = 1.72, 3.58 ppm, MeOH-d_4 = 3.31 ppm. For ^{13}C NMR: $CDCl_3$ = 77.16 ppm, CD_2Cl_2 = 53.84 ppm, benzene-d_6 = 128.06 ppm, toluene-d_8 = 20.43, cyclohexane-d_{12} = 26.43 ppm, THF-d_8 = 67.57, 23.37 ppm, MeOH-d_4 = 49.00 ppm The resonance multiplicity is indicated as *s* (singlet), *d* (doublet), *t* (triplet), *q* (quartet) and *m* (multiplet). The chemical shifts are given in parts per million (ppm) on the delta scale (δ). The coupling constant values (J) are given in hertz (Hz).

Electron Impact (EI) mass spectra were measured on a *Finnigan MAT95Q* or on a *Finnigan MAT90* mass spectrometer. **Electronspray ionisation (ESI) mass spectra were** measured on a *Thermo Finnigan LTQ-FT*. The most important signals are reported in *m/z* units with *M* as the molecular ion.

Elemental analysis were performed in the micro analytical laboratory of the LMU department of chemistry on an *Elementar Vario EL* apparatus.

Infrared spectra were recorded on a *Perkin Elmer Spectrum BX-FT-IR* instrument equipped with a *Smith DuraSamplIR II ATR*-device. Transmittance values are qualitatively described by wavenumber (cm^{-1}) as very strong (vs), strong (s), medium (m) and weak (w).

UV/Vis spectra were measured on a *Varian Cary 5000* spectrophotometer. The spectra were recorded in a quartz cuvette (1 cm). Solvents for spectroscopy were obtained from *VWR* and *Merck*. Absorption

wavelength (λ) are reported in nm and the molar absorption coefficients (ε) in L mol^{-1} cm^{-1}. Shoulders are declared as sh.

Low temperature UV/vis spectra in EPA glass (ether/isopentane/ethanol 5:5:2) at 90 K were measured on a *Varian Cary® 50* spectrophotometer with an *Oxford DN 1704* optical cryostat controlled by an *Oxford ITC 4* device. Low temperatures were reached by cooling slowly with liquid nitrogen. The spectra were recorded in a quartz cuvette (1 cm). Solvents for spectroscopy were obtained from *VWR*, *Merck* and *Sigma Aldrich* and were dried, degassed and filtrated prior use. For irradiation studies a *Mightex FCS-0405-200 LED* (405 nm) was used as light source. Absorption wavelength (λ) are reported in nm and the molar absorption coefficients (ε) in L·mol^{-1}·cm^{-1}.

Melting points (M.p.) were measured on a *Stuart* SMP10 melting point apparatus in open capillaries and are not corrected.

Photoisomerization experiments. Continuous irradiations of the solutions were conducted in NMR tubes in different solvents (CD_2Cl_2, $(CDCl_2)_2$, benzene-d_6, toluene-d_8, MeOH-d_4, DMSO-d_6, EPA, EG). Irradiations were conducted using LEDs from Roithner Lasertechnik GmbH (305 nm, 365 nm, 405 nm). For low temperature studies a *Mightex FCS-0405-200 LED* (405 nm) was used as light source and the light beam was guided by a fiber-optic cable (0.39 NA, one SMA, one blank end) and pointed directly into the NMR tube during NMR measurements.

HTI Synthesis

2-((3,3-Dimethyl-2-oxobutyl)thio)benzoic acid (**2**)

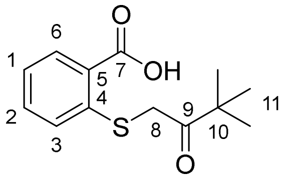

Thiosalicylic acid (50 mmol, 1.0 equiv.) and sodium acetate (100 mmol, 2.0 equiv.) were added to the *in situ* generated 1-bromo-3, 3-dimethyl-2-butanone (50 mmol, 1.0 equiv., based on used ketone) in methanol (1 M solution) and the solution was stirred for 30 min at 23 °C. Afterwards the reaction suspension was poured on ice water and the reaction product was filtered off, washed with water and hexanes and dried *in vacuo*. The title compound (**2**) was isolated as colorless solid (12.4 g, 49 mmol, 98%).[1]

^{1}H NMR (400 MHz, $CDCl_3$) δ = 8.08 (dd, ^{3}J(H,H) = 8.0 Hz, ^{4}J(H,H) = 1.6 Hz, 1H, H-C(6)), 7.47 (ddd, ^{3}J(H,H) = 8.0 Hz, ^{3}J(H,H) = 7.8 Hz, ^{4}J(H,H) = 1.6 Hz, 1H, H-C(2)), 7,34 (dd, ^{3}J(H,H) = 7.8 Hz, ^{4}J(H,H) = 1.6 Hz, 1H, H-C(3)), 7.25 (td, ^{3}J(H,H) = 8.0 Hz, ^{4}J(H,H) = 1.6 Hz, 1H, H-C(1)), 4.02 (s, 2H, H-C(8)), 1.24 (s, 9H, H-C(11)) ppm.

^{13}C NMR (101 MHz, $CDCl_3$) δ = 209.9 (C(9)), 170.9 (C(7)), 140.1 (C(4)), 133.2 (C(2)), 132.4 (C(6)), 128.3 (C(5)), 127.7 (C(3)), 125.4 (C(1)), 44.7 (C(8)), 39.3 (C(10)), 26.8 (C(11)) ppm.

IR $\tilde{\nu}$ (cm^{-1}) = 2969 (w), 2643 (w), 1684 (vs), 1583 (w), 1561 (w), 1472 (w), 1460 (m), 1404 (m), 1395 (m), 1367 (w), 1314 (m), 1289 (m), 1255 (vs), 1151 (w), 1058 (m), 1050 (m), 1044 (s), 917 (s), 815 (w), 803 (w), 793 (w), 734 (vs), 703 (m), 686 (w).

Melting point: 161°C.

HRMS (EI^+), [M^+]: *m/z* calcd. 252.0815 für $[C_{13}H_{16}O_3S]^+$, found 252.0815.

R_f (SiO_2, *i*Hex/EtOAc = 50/50) = 0.12.

1-(3-Hydroxybenzo[*b*]thiophen-2-yl)-2,2-dimethylpropan-1-one (**3**)

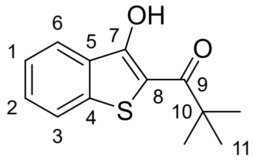

2-((3,3-Dimethyl-2-oxobutyl)thio)benzoic acid (**2**, 40 mmol, 1 equiv.) was dissolved in DMF to receive a 1 M solution, sodium acetate (120 mmol, 3.0 equiv.) was added and the solution was heated to 140 °C for 4 h under continuous stirring. After the mixture was cooled down to room temperature, water (50 ml) was added, the product was extracted with dichloromethane (3 x 50 ml) and dried over Na_2SO_4. The combined organic phases were removed *in vacuo* and the title compound (**3**) was isolated after crystallization from hexanes/dichloromethane as light yellow crystals (9.1 g, 39 mmol, 97%).[1]

^{1}H NMR (400 MHz, $CDCl_3$) δ = 13.51 (s, 1H, H-O), 8.06 (d, ^3J(H,H) = 9.1 Hz, 1H, H-C(6)), 7.70 (d, ^3J(H,H) = 8.1 Hz, 1H, H-C(3)), 7.52 (t, ^3J(H,H) = 8.1 Hz, 1H, H-C(2)), 7.38 (t, ^3J(H,H) = 8.1 Hz, 1H, H-C(1)), 1.43 (s, 9H, H-C(11)) ppm.

^{13}C NMR (101 MHz, $CDCl_3$) δ = 206.1 (C(9)), 165.3 (C(7)), 139.6 (C(4)), 130.4 (C(5)), 130.3 (C(2)), 124.9 (C(1)), 124.0 (C(6)), 123.0 (C(3)), 107.9 (C(8)), 44.0 (C(10)), 27.6 (C(11)) ppm.

IR $\tilde{\nu}$ (cm^{-1}) = 2969 (w), 2643 (w), 1684 (vs), 1583 (w), 1561 (w), 1472 (w), 1460 (m), 1404 (m), 1395 (m), 1367 (w), 1314 (m), 1289 (m), 1255 (vs), 1151 (w), 1058 (m), 1050 (m), 1044 (s), 917 (s), 815 (w), 803 (w), 793 (w), 734 (vs), 703 (m), 686 (w).

Melting point: 161°C.

HRMS (EI$^+$), [M$^+$]: *m/z* calcd. 234.0715 für $[C_{13}H_{14}O_2S]^+$, found 234.0716.

R_f (SiO_2, *i*Hex/EtOAc = 98/2) = 0.56.

(*Z*)-2-(1-Chloro-2,2-dimethylpropylidene)benzo[*b*]thiophen-3(2*H*)-one (**4**)

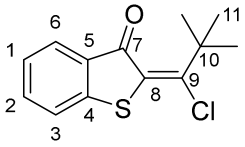

1-(3-Hydroxybenzo[*b*]thiophen-2-yl)-2,2-dimethylpropan-1-one (**3**, 8.5 mmol, 1 equiv.) was dissolved in toluene to receive a 1 M solution. Thionylchloride (2.0 equiv.) and DMF (0.1 equiv.) were added and the solution was stirred at 50 °C for 3 h. Saturated sodium carbonate solution was added until the solution was neutralized, the product was extracted with dichloromethane (3 x 50 mL) and the combined organic phases were dried over Na_2SO_4. The solvent was removed *in vacuo*. The product was purified by column chromatography over a short plug of silica (SiO_2, *i*Hex/EtOAc = 99/1 → 95/5) and further by crystallization from *n*heptane as light yellow solid (2.15 g, 8.5 mmol, quant., only the *Z* isomer was obtained after crystallization).[1]

^{1}H NMR (400 MHz, CD_2Cl_2) δ = 7.75 (dd, ^3J(H,H) = 7.8, ^4J(H,H) = 1.4, 1H, H-C(6)), 7.55 (ddd, ^3J(H,H) = 7.9, ^3J(H,H) = 7.2, ^4J(H,H) = 1.3 Hz, 1H, H-C(2)), 7.39 (dd, ^3J(H,H) = 7.9, ^4J(H,H) = 0.9 Hz, 1H, H-C(3)), 7.25 (ddd, ^3J(H,H) = 8.1, ^3J(H,H) = 7.3, ^4J(H,H) = 1.0 Hz, 1H, H-C(1)), 1.49 (s, 9H, H-C(11)) ppm.

^{13}C NMR (101 MHz, CD_2Cl_2) δ = 184.0 (C(7)), 159.3 (C(9)), 144.9 (C(4)), 135.5 (C(2)), 134.3 (C(8)), 134.3 (C(5)), 127.3 (C(6)), 125.8 (C(1)), 123.5 (C(3)), 42.1 (C(10)), 28.8 (C(11)) ppm.

IR $\tilde{\nu}$ (cm^{-1}) = 2975 (w), 1583 (vs), 1509 (s), 1477 (m), 1461 (w), 1400 (w), 1390 (w), 1366 (m), 1334 (m), 1304 (s), 1288 (m), 1275 (m), 1239 (m), 1205 (vs), 1172 (m), 1129 (w), 1095 (s), 1063 (m), 999 (vs), 940 (w), 856 (w), 821 (m), 774 (s), 750 (s), 735 (vs).

Melting point: 51°C.

HRMS (EI$^+$), [M$^+$]: *m/z* calcd. 252.0370 für $[C_{13}H_{13}OClS]^+$, found 252.0361.

R_f (SiO_2, *i*Hex/EtOAc = 98/2) = 0.51.

(Z)-2-(1-(2-Methoxyphenyl)-2,2-dimethylpropylidene)benzo[*b*]thiophen-3(2*H*)-one (**5**)

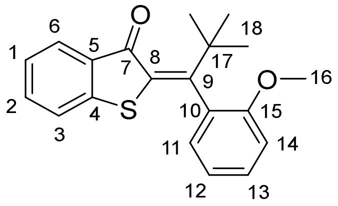

(*Z*)-2-(1-Chloro-2,2-dimethylpropylidene)benzo[*b*]thiophen-3(2*H*)-one (**4**, 1.1 mmol 1.0 equiv.) was dissolved in toluene (18 mL). (2-Methoxyphenyl)boronic acid (1.65 mmol, 1.5 equiv.), potassium carbonate (2.2 mmol, 2.0 equiv.) and water (2 mL) were added. After degassing with argon, $Pd(PPh_3)_4$ (5 mol%) was added and the reaction mixture was stirred for 24 h at 80 °C. Subsequently, saturated sodium carbonate solution (5 mL) was added, the aqueous phase was extracted with dichloromethane (3 x 20 mL) and the combined organic phases were dried over Na_2SO_4. After removing the solvent *in vacuo*, the crude product was purified by column chromatography (SiO_2, *i*Hex/EtOAc = 95/5) and further by crystallization from *n*heptane. The title compound was isolated as yellow crystals (341 mg, 1.05 mmol, 96%, only the *Z* isomer was obtained after crystallization).

^{1}H NMR (400 MHz, $CDCl_3$) δ = 7.81 (dd, ^3J(H,H) = 8.3 Hz, ^4J(H,H) = 1.3 Hz, 1H, H-C(6)), 7.43 (ddd, ^3J(H,H) = 7.9 Hz, ^3J(H,H) = 7.2 Hz, ^4J(H,H) = 1.4 Hz, 1H, H-C(2)), 7.37 (ddd, ^3J(H,H) = 8.5 Hz, ^3J(H,H) = 7.2 Hz, ^4J(H,H) = 1.4 Hz, 1H, H-C(13)), 7.21 – 7.15 (m, 2H, H-C(1), H-C(3)), 7.04 – 7.00 (m, 2H, H-C(11), H-C(12)), 6.99 – 6.94 (m, 1H, H-C(14)), 3.82 (s, 3H, H-C(16)), 1.36 (s, 9H, H-C(18)) ppm.

^{13}C NMR (101 MHz, $CDCl_3$) δ = 186.4 (C(7)), 163.93 (C(9)), 155.4 (C(15)), 145.9 (C(4)), 134.5 (C(2)), 3x 133.7 (C(5,8,12)), 129.4 (C(13)), 128.4 (C(11)), 126.8 (C(6)), 124.5 (C(3)), 122.8 (C(1)), 120.6 (C(12)), 111.0 (C(14)), 55.7 (C(16)), 38.7 (C(17)), 28.6 (C(18)) ppm.

IR $\tilde{\nu}$ (cm^{-1}) = 2960 (w), 2910 (w), 1667 (vs), 1588 (s), 1536 (m), 1487 (m), 1451 (s), 1433 (w), 1390 (w), 1359 (w), 1313 (w), 1280 (s), 1257 (m), 1239 (m), 1218 (m), 1178 (w), 1160 (w), 1116 (m), 1069 (s), 1047 (w), 1024 (m), 953 (w), 929 (w), 899 (w), 821 (w), 743 (vs), 670 (w).

Melting point: 109 °C.

HRMS (EI$^+$), [M$^+$]: *m/z* calcd: 324.1179 for $[C_{20}H_{20}O_2S]^+$, found: 324.1179.

R_f (SiO_2, *i*Hex/EtOAc = 98/2) = 0.42.

2-(1-(2-Methoxyphenyl)-2,2-dimethylpropylidene)benzo[*b*]thiophen-3(2*H*)-one-1-oxide (**1**)

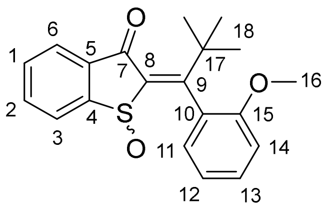

2-(1-(2-Methoxyphenyl)-2,2-dimethylpropylidene)benzo[*b*]thiophen-3(2*H*)-one (**5**, 0.55 mmol 1.0 equiv.) was dissolved in acetic acid (10 mL). Sodium perborate (0.60 mmol, 1.1 equiv.) was added and the reaction mixture was stirred for 12 h at 23 °C. Subsequently, saturated sodium carbonate solution (15 mL) was added, the aqueous phase was extracted with dichloromethane (3 x 20 mL) and the combined organic phases were dried over Na_2SO_4. After removing the solvent *in vacuo*, the crude product was purified by column chromatography (SiO_2, *i*Hex/EtOAc = 1/1) as yellow solid (182 mg, 0.53 mmol, 97%) as a mixture of four isomers. These isomers could be separated by column chromatography in the dark (SiO_2, *i*Hex/EtOAc = 6/4) and were subsequently purified further by individual crystallization from *n*heptane/dichloromethane.

A-1

^{1}H NMR (600 MHz, CD_2Cl_2) δ = 7.97 (dt, ^3J(H,H) = 7.7, ^4J(H,H) = 1.0 Hz, 1H, H-C(6)), 7.83 – 7.77 (m, 2H, H-C(2), H-C(3)), 7.71 (ddd, ^3J(H,H) = 7.7, ^3J(H,H) = 6.4, ^4J(H,H) = 2.0 Hz, 1H, H-C(1)), 7.44 (ddd, ^3J(H,H) = 8.4, ^3J(H,H) = 7.2, ^4J(H,H) = 2.0 Hz, 1H, H-C(13)), 7.08 – 7.01 (m, 2H, H-C(11), H-C(12)), 6.99 (d, ^3J(H,H) = 8.4 Hz, 1H, H-C(14)), 3.81 (s, 3H, H-C(16)), 1.37 (s, 9H, H-C(18)) ppm.

^{13}C NMR (150 MHz, CD_2Cl_2) δ = 185.1 (C(7)), 180.2 (C(9)), 157.6 (C(15)), 149.0 (C(4)), 143.6 (C(8)), 136.7 (C(5)), 136.1 (C(2)), 133.1 (C(1)), 130.8 (C(10)), 130.6 (C(13)), 128.0 (C(11)), 127.5 (C(3)), 126.4 (C(6)), 120.7 (C(12)), 111.5 (C(14)), 56.0 (C(16)), 40.1 (C(17)), 28.8 (C(18)) ppm.

IR $\tilde{\nu}$ (cm^{-1}) = 1683 (vs), 1653 (w), 1591 (m), 1580 (m), 1551 (s), 1488 (s), 1457 (m), 1433 (m), 1392 (w), 1360 (w), 1274 (m), 1258 (s), 1221 (s), 1161 (w), 1117 (m), 1072 (m), 1044 (s), 1023 (m), 955 (m), 930 (w), 820 (w), 752 (vs), 703 (w), 678 (m), 609 (m).

Melting point: 134 °C.

R_f (SiO_2, *i*Hex/EtOAc = 1/1) = 0.15.

B-1

^{1}H NMR (600 MHz, CD_2Cl_2) δ = 7.96 (dt, ^3J(H,H) = 7.7, 1.0 Hz, 1H, H-C(6)), 7.81 – 7.76 (m, 2H, H-C(2), H-C(3)), 7.71 (ddd, ^3J(H,H) = 7.7, ^3J(H,H) = 6.3, ^4J(H,H) = 2.1 Hz, 1H, H-C(1)), 7.44 (ddd, ^3J(H,H) = 8.4, ^3J(H,H) = 7.4, ^4J(H,H) = 1.8 Hz, 1H, H-C(13)), 7.13 (dd, ^3J(H,H) = 7.4, ^4J(H,H) = 1.8 Hz, 1H, H-C(11)), 7.07 (td, ^3J(H,H) = 7.4, ^4J(H,H) = 1.0 Hz, 1H, H-C(12)), 7.01 (dd, ^3J(H,H) = 8.4, ^4J(H,H) = 0.9 Hz, 1H, H-C(14)), 3.78 (s, 3H, H-C(16)), 1.33 (s, 9H, H-C(18)) ppm.

^{13}C NMR (150 MHz, CD_2Cl_2) δ = 186.6 (C(7)), 179.0 (C(9)), 155.6 (C(15)), 148.8 (C(4)), 142.9 (C(8)), 136.6 (C(5)), 136.3 (C(2)), 133.2 (C(1)), 130.7 (C(11)), 130.6 (C(13)), 129.3 (C(10)), 127.6 (C(3)), 126.7 (C(6)), 120.2 (C(12)), 111.0 (C(14)), 55.9 (C(16)), 39.8 (C(17)), 29.3 (C(18)) ppm.

IR $\tilde{\nu}$ (cm^{-1}) = 1686 (vs), 1653 (w), 1616 (w), 1592 (m), 1580 (m), 1557 (m), 1521 (w), 1506 (w), 1487 (m), 1457 (m), 1435 (w), 1419 (w), 1393 (w), 1361 (w), 1321 (w), 1274 (m), 1255 (m), 1236 (m), 1220 (s), 1162 (w), 1118 (m), 1067 (m), 1038 (s), 955 (m), 930 (w), 851 (w), 820 (w), 800 (w), 752 (vs), 720 (w), 704 (m), 679 (m), 667 (w), 607 (w).

Melting point: 183 °C.

R_f (SiO_2, *i*Hex/EtOAc = 1/1) = 0.21.

C-1

^{1}H NMR (600 MHz, CD_2Cl_2) δ = 8.02 (dt, ^3J(H,H) = 7.8, ^4J(H,H) = 0.8 Hz, 1H, H-C(3)), 7.84 (t, ^3J(H,H) = 8.4 Hz, 1H, H-C(2)), 7.75 (dt, ^3J(H,H) = 8.0, ^4J(H,H) = 0.9 Hz, 1H, H-C(6)), 7.67 (td, ^3J(H,H) = 7.5, ^4J(H,H) = 1.0 Hz, 1H, H-C(1)), 7.37 (ddd, ^3J(H,H) = 8.3, ^3J(H,H) = 7.4, ^4J(H,H) = 1.7 Hz, 1H, H-C(13)), 7.00 (td, ^3J(H,H) = 7.5, ^4J(H,H) = 1.0 Hz, 1H, H-C(12)), 6.95 (dd, ^3J(H,H) = 8.3, ^4J(H,H) = 1.0 Hz, 1H, H-C(14)), 6.90 (dd, ^3J(H,H) = 7.4, ^4J(H,H) = 1.7 Hz, 1H, H-C(11)), 3.73 (s, 3H, H-C(16)), 1.46 (s, 9H, H-C(18)) ppm.

^{13}C NMR (150 MHz, CD_2Cl_2) δ = 185.5 (C(7)), 175.1 (C(9)), 156.3 (C(15)), 148.4 (C(4)), 140.0 (C(8)), 136.3 (C(2)), 134.7 (C(5)), 133.2 (C(1)), 130.1 (C(10)), 129.2 (C(13)), 127.5 (C(3)), 126.9 (C(11)), 126.2 (C(6)), 120.9 (C(14)), 110.7 (C(12)), 55.8 (C(16)), 41.1 (C(17)), 30.2 (C(18)) ppm.

IR $\tilde{\nu}$ (cm^{-1}) = 1691 (s), 1593 (w), 1551 (m), 1487 (m), 1463 (m), 1451 (m), 1433 (m), 1393 (w), 1360 (w), 1274 (m), 1256 (w), 1227 (m), 1115 (s), 1072 (m), 1057 (m), 1023 (s), 933 (w), 863 (w), 828 (w), 801 (w), 777 (w), 753 (vs), 700 (m), 675 (m).

Melting point: 145 °C.

R_f (SiO_2, *i*Hex/EtOAc = 1/1) = 0.42.

D-1

^{1}H NMR (800 MHz, CD_2Cl_2) δ = 8.00 (d, ^{3}J(H,H) = 7.7 Hz, 1H, H-C(3)), 7.84 (t, ^{3}J(H,H) = 7.8 Hz, 1H, H-C(2)), 7.74 (d, ^{3}J(H,H) = 7.6 Hz, 1H, H-C(1)), 7.68 (t, ^{3}J(H,H) = 7.4 Hz, 1H, H-C(6)), 7.36 (t, ^{3}J(H,H) = 7.5 Hz, 1H, H-C(13)), 7.00 (d, ^{3}J(H,H) = 8.1 Hz, 1H, H-C(14)), 6.94 (t, ^{3}J(H,H) = 7.5 Hz, 1H, H-C(12)), 6.68 (d, ^{3}J(H,H) = 8.8 Hz, 1H, H-C(11)), 3.82 (s, 3H, H-C(16)), 1.45 (s, 9H, H-C(18)) ppm.

^{13}C NMR (200 MHz, CD_2Cl_2) δ = 187.5 (C(7)), 174.6 (C(9)), 156.5 (C(15)), 148.4 (C(4)), 139.5 (C(8)), 136.4 (C(2)), 134.3 (C(5)), 133.4 (C(6)), 130.1 (C(10)), 129.3 (C(13)), 127.5 (C(3)), 126.5 (C(11)), 126.4 (C(1)), 120.8 (C(12)), 111.0 (C(14)), 56.0 (C(16)), 40.9 (C(17)), 31.4 (C(18)) ppm.

IR $\tilde{\nu}$ (cm^{-1}) = 1693 (s), 1580 (w), 1556 (m), 1488 (m), 1451 (m), 1430 (w), 1364 (w), 1325 (w), 1276 (m), 1253 (m), 1221 (s), 1200 (w), 1176 (w), 1156 (w), 1116 (m), 1057 (w), 1044 (w), 1026 (vs), 996 (m), 928 (w), 865 (w), 848 (w), 829 (w), 801 (w), 779 (w), 755 (vs), 722 (w), 702 (m), 677 (m), 655 (m).

Melting point: 192 °C.

R_f (SiO_2, *i*Hex/EtOAc = 1/1) = 0.31.

For all isomers:

HRMS (EI^+), [M^+]: *m/z* calcd: 340.1122 for $[C_{20}H_{20}O_3S]^+$, found: 340.1126.

Elemental analysis: calcd (%) for $C_{20}H_{20}O_3S$: C 70.56, H 5.92, S 9.42; found: C 70.26, H 5.85, S 9.49.

Physical and photophysical properties

Thermal atropisomerizations A to B and C to D

NMR tubes were charged with 0.8 mg to 2.5 mg of respective isomer **A**, **B**, **C**, or **D** and 0.7 mL of deuterated solvent. Subsequent heating was carried out in amberized NMR tubes at 82 °C or 100 °C. Kinetics were followed by ^{1}H NMR measurements at defined time intervals. The equilibrium concentrations of isomers after prolonged heating were obtained from integration of the corresponding signals in the ^{1}H NMR spectrum.

At 82 °C or 100 °C only the atropisomerizations between **A** and **B** and between **C** and **D** proceed. The thermal atropisomerizations follow unimolecular first order reactions and proceed towards an equilibrium composition with both atropisomers present, according to Supplementary Equation 1 and Supplementary Equation 2, respectively:

$$\ln\left(\frac{[\mathbf{A}]_{t_0}-[\mathbf{A}]_{eq}}{[\mathbf{A}]_t-[\mathbf{A}]_{eq}}\right) = (k_{\mathbf{A/B}} + k_{\mathbf{B/A}})t \qquad \text{(Supplementary Equation 1)}$$

$$\ln\left(\frac{[\mathbf{C}]_{t_0}-[\mathbf{C}]_{eq}}{[\mathbf{C}]_t-[\mathbf{C}]_{eq}}\right) = (k_{\mathbf{C/D}} + k_{\mathbf{D/C}})t \qquad \text{(Supplementary Equation 2)}$$

with [**A** or **C**]$_{t_0}$ being the initial concentration of the isomers **A** or **C** at the time $t = 0$, [**A** or **C**]$_{eq}$ being the concentration of the isomers **A** or **C** at equilibrium, [**A** or **C**]$_t$ representing the concentration of the isomers **A** or **C** at specific times in the measurement t, $k_{\mathbf{A/B}}$ being the rate constant k of the **A** to **B** conversion, $k_{\mathbf{B/A}}$ being the rate constant k of the **B** to **A** conversion, $k_{\mathbf{C/D}}$ being the rate constant k of the **C** to **D** conversion, $k_{\mathbf{D/C}}$ being the rate constant k of the **D** to **C** conversion, and t being the elapsed time. When plotting the logarithmic left part of Supplementary Equation 1 or Supplementary Equation 2 versus time t, the obtained slope m contains rate constants for both proceeding atropisomerization reactions. The rate constants $k_{\mathbf{A/B}}$ and $k_{\mathbf{C/D}}$ can be calculated according to Supplementary Equation 3 and Supplementary Equation 4:

$$k_{\mathbf{A/B}} = \frac{m}{1+\frac{[\mathbf{A}]_{eq}}{[\mathbf{B}]_{eq}}} \qquad \text{(Supplementary Equation 3)}$$

$$k_{\mathbf{C/D}} = \frac{m}{1+\frac{[\mathbf{C}]_{eq}}{[\mathbf{D}]_{eq}}} \qquad \text{(Supplementary Equation 4)}$$

If the corresponding laws of mass action (Supplementary Equation 5 and Supplementary Equation 6) are taken into account:

$$\frac{[\mathbf{A}]_{\mathrm{eq}}}{[\mathbf{B}]_{\mathrm{eq}}} = \frac{k_{\mathbf{B/A}}}{k_{\mathbf{A/B}}} \qquad \text{(Supplementary Equation 5)}$$

$$\frac{[\mathbf{C}]_{\mathrm{eq}}}{[\mathbf{D}]_{\mathrm{eq}}} = \frac{k_{\mathbf{D/C}}}{k_{\mathbf{C/D}}} \qquad \text{(Supplementary Equation 6)}$$

Likewise the reverse rate constants are defined and could be determined from separate measurements starting from the opposite pure isomers **B** and **D** and application of Supplementary Equation 7 to Supplementary Equation 10, respectively.

$$\ln\left(\frac{[\mathbf{B}]_{\mathrm{t_0}}-[\mathbf{B}]_{\mathrm{eq}}}{[\mathbf{B}]_{t}-[\mathbf{B}]_{\mathrm{eq}}}\right) = (k_{\mathbf{A/B}} + k_{\mathbf{B/A}})t \qquad \text{(Supplementary Equation 7)}$$

$$\ln\left(\frac{[\mathbf{D}]_{t_0}-[\mathbf{D}]_{\mathrm{eq}}}{[\mathbf{D}]_{t}-[\mathbf{D}]_{\mathrm{eq}}}\right) = (k_{\mathbf{C/D}} + k_{\mathbf{D/C}})t \qquad \text{(Supplementary Equation 8)}$$

$$k_{\mathbf{B/A}} = \frac{m}{1+\frac{[\mathbf{B}]_{\mathrm{eq}}}{[\mathbf{A}]_{\mathrm{eq}}}} \qquad \text{(Supplementary Equation 9)}$$

$$k_{\mathbf{D/C}} = \frac{m}{1+\frac{[\mathbf{D}]_{\mathrm{eq}}}{[\mathbf{C}]_{\mathrm{eq}}}} \qquad \text{(Supplementary Equation 10)}$$

$$\Delta G^{\ddagger} = \ln\left(\frac{k*h}{k_{\mathrm{B}}*T}\right) * R * T \qquad \text{(Supplementary Equation 11)}$$

By using the *Eyring* equation (Supplementary Equation 11) the free activation enthalpies ΔG^{*} can be calculated from the rate constants $k_{\mathbf{A/B}}$ to $k_{\mathbf{D/C}}$ for the corresponding reaction. The obtained free activation enthalpies ΔG^{*} for the thermal atropisomerizations between **A** to **B** as well as **C** to **D** and *vice versa* in $(CDCl_2)_2$ and the corresponding half-lives at 25 °C are given in Supplementary Table 1 together with the equilibrium atropisomer compositions obtained at high temperatures.

From these measurements we could quantify the ground state energy profile of hemithioindigo **1**. As we did not observe any thermal double-bond isomerizations over the course of several hours at temperatures >100 °C a lower limit for the energy barriers could be given, which are at least 30 kcal/mol high for each double-bond isomerization. The equilibrium atropisomer compositions at high temperatures deliver the thermodynamic energy differences between the corresponding states (red Δ values in Supplementary Figure 9) according to the relation of the change of *Gibbs* free energy and the equilibrium constant $-\Delta G = R{\cdot}T{\cdot}\ln K$

(see also Supplementary Table 1). The theoretical values are in good agreement with the experimentally determined ones.

Photoconversion of A, B, C, and D determined by quantum yield measurements

The photochemical quantum yield of the different photoconversion reactions ϕ were calculated as the ratio between the numbers of isomerized molecules n(molecules isomerized) and the number of absorbed photons $n(hv)$ according to Supplementary Equation 12:

$$\phi = \frac{n(\text{molecules isomerized})}{n(hv)} \qquad \text{(Supplementary Equation 12)}$$

To determine the quantum yields ϕ, a sample with known concentration (3.0 mM) of each pure isomer **A**, **B**, **C**, or **D** in benzene-d_6 was irradiated with a focused light beam of a 405 nm LED within the published instrumental setup from the group of *E. Riedle*.[2] The number of absorbed photons over time $n(hv)$ was measured directly at the thermal photometer of the instrument according to Supplementary Equation 13:

$$n(hv) = \frac{\Delta P \cdot \lambda_{\text{ex}} \cdot t}{c \cdot h} \qquad \text{(Supplementary Equation 13)}$$

Where c is the speed of light ($2.99792 \cdot 10^{8}$ m·s^{-1}), h is Planck's constant ($6.62607 \cdot 10^{-34}$ J·s), λ_{ex} is the excitation wavelength in m, t is the elapsed time during irradiation, and ΔP is the difference in power read-outs at the thermal photometer between a solvent filled cuvette (P_0) and a cuvette containing the sample solution (P_t) during the irradiation period in Watt (Supplementary Equation 14). The power read out during irradiation did not change substantially (<5%) over the irradiation periods.

$$\Delta P = P_t - P_0 \qquad \text{(Supplementary Equation 14)}$$

Since more than one photoproduct is formed during irradiation, the number of each type of photoconverted molecules n(molecules isomerized) was determined by ^{1}H NMR spectroscopy directly after the irradiation step. Multiple measurements with increasing time of irradiation were conducted and the obtained ϕ values were averaged. The amount of isomerized molecules was also plotted against irradiation time showing linear

behavior over measurement periods up to 30 min in case of **A**, **B**, and **C** (Supplementary Figure 14 - 16). Therefore, the quantum yield measurements conform to initial-slope behavior, where only the initial isomer is photoconverted but not the photoproducts. The photoconversion of **D** did not conform very well to linear behavior (Supplementary Figure 17). Nevertheless, the initial slope assumption gave good starting points for the comprehensive analysis of all quantum yields ϕ using a rate matrix as described below.

For hemithioindigo **1** the rate of an individual phototransition e.g. **A** to **B**, depends on all other absorbing species present at the same time and is described by the corresponding rate matrix element $r_{\mathbf{A/B}}$ (Supplementary Equation 15):

$$r_{\mathbf{A/B}} = \phi_{\mathbf{A/B}} \cdot I_0 \cdot \varepsilon_{\mathbf{A}} \cdot d \cdot [\mathrm{A}] \cdot \left(\frac{1-e^{-d\sum_{\mathrm{i}} \varepsilon_{\mathrm{i}} \cdot [\mathrm{i}]}}{d\sum_{\mathrm{i}} \varepsilon_{\mathrm{i}} \cdot [\mathrm{i}]}\right) \qquad \text{(Supplementary Equation 15)}$$

Likewise every phototransition from isomer **i** to isomer **j** can be written as:

$$r_{\mathbf{i/j}} = \phi_{\mathbf{i/j}} \cdot I_0 \cdot \varepsilon_{\mathrm{i}} \cdot d \cdot [\mathrm{i}] \cdot \left(\frac{1-e^{-d\sum_{\mathrm{i}} \varepsilon_{\mathrm{i}} \cdot [\mathrm{i}]}}{d\sum_{\mathrm{i}} \varepsilon_{\mathrm{i}} \cdot [\mathrm{i}]}\right) \qquad \text{(Supplementary Equation 16)}$$

with $\phi_{\mathbf{i/j}}$ = photoisomerization quantum yield for the phototransition of **i** to **j**, I_0 the photon flux (Einstein L^{-1} s^{-1}) of the light, ε_{i} the molar absorption coefficient of **i** at the wavelength of irradiation, d the pathlength of the light through the sample, [**i**] the concentration of **i**, ε_{i} the molar absorption coefficient of species **i**. The corresponding rate matrix containing all possible rates $r_{\mathbf{i/j}}$ for the transitions of isomers **i** into isomers **j** for hemithioindigo **1** is therefore written as:

$$M_1 = \begin{pmatrix} r_{A/A} & r_{A/B} & r_{A/C} & r_{A/D} \\ r_{B/A} & r_{B/B} & r_{B/C} & r_{B/D} \\ r_{C/A} & r_{C/B} & r_{C/C} & r_{C/D} \\ r_{D/A} & r_{D/B} & r_{D/C} & r_{D/D} \end{pmatrix} \qquad \text{(Supplementary Equation 17)}$$

(columns A, B, C, D; rows A, B, C, D)

Matrix M_1 (Supplementary Equation 17) describes all possible photoconversions quantitatively, but each element is a nonlinear differential equation dependent on the other elements at a given time, which makes an analytical solution impossible.

We have used the different rate elements $r_{i/j}$ in the rate matrix M_1 to simulate our quantum yield measurements. For every incremental irradiation step (Δt) we have used the following expressions (Supplementary Equation 18 - 21) to achieve the corresponding next concentration of the respective isomer:

$$[A]_{t+1} = [A]_t - r_{A/B}\Delta t - r_{A/C}\Delta t - r_{A/D}\Delta t + r_{B/A}\Delta t + r_{C/A}\Delta t + r_{D/A}\Delta t \quad \text{(Supplementary Equation 18)}$$

$$[B]_{t+1} = [B]_t - r_{B/A}\Delta t - r_{B/C}\Delta t - r_{B/D}\Delta t + r_{A/B}\Delta t + r_{C/B}\Delta t + r_{D/B}\Delta t \quad \text{(Supplementary Equation 19)}$$

$$[C]_{t+1} = [C]_t - r_{C/A}\Delta t - r_{C/B}\Delta t - r_{C/D}\Delta t + r_{A/C}\Delta t + r_{B/C}\Delta t + r_{D/C}\Delta t \quad \text{(Supplementary Equation 20)}$$

$$[D]_{t+1} = [D]_t - r_{D/A}\Delta t - r_{D/B}\Delta t - r_{D/C}\Delta t + r_{A/D}\Delta t + r_{B/D}\Delta t + r_{C/D}\Delta t \quad \text{(Supplementary Equation 21)}$$

Since every component of $r_{i/j}$ (Supplementary Equation 16) is known from our experiment except for the quantum yields ϕ_{ij} the latter can be obtained from the best fit to the rate model M_1 (Supplementary Equation 17). To this end we have started the simulation with experimental quantum yield values obtained from the initial slope analyses described above. 10,000 incremental time points were used in the simulations and the quantum yields were adjusted manually until an adequate match of experimental and simulated data were obtained. While the quantum yields of **A** and **B** obtained from the simulation were very similar to the ones obtained from the initial slope method (maximum ±2% deviation), the quantum yields of **C** and **D** were considerably improved by the simulation. This can clearly be seen by the much better description of the nonlinear photoconversions of **C** and **D** using the optimized quantum yields from the fitting simulation (Supplementary Figure 18).

Markov matrix analysis of the photoconversion of A, B, C, and D in different solvents

The photoconversions of pure **A**, **B**, **C**, and **D** in different solvents were determined by *in situ* irradiations within the NMR spectrometer at ambient temperature (exact temperatures are given in Supplementary Figures 20 - 43 for each experiment) while recording a ^{1}H NMR spectrum in short time intervals (typically several seconds) for prolonged overall times. For the *in situ* irradiations, a fiber-coupled LED setup from *Mightex* (UHP 405 nm LED) was used. To ensure very high reproducibility and comparability between individual experiments, the glass fiber was fixed to the NMR tubes in the same position for each irradiation and its light intensity kept constant. With this setup it is however not possible to determine the light pathlength *d*. The same concentrations (3 mM) of the pure initial isomer and the same solvent volumes were used as well.

The relative percentage of each isomer **A**, **B**, **C**, and **D** was then plotted against irradiation time for each pure starting isomer. These kinetic data were then simulated using an adjusted Markov matrix accounting for the different conversion probabilities per time increment (1 min in this case).

In general a Markov matrix describes the probabilities for different transitions of different states p(**ij**) within a given time increment. For a photoreaction the probabilities are directly proportional to the rate $r_{\mathbf{i/j}}$ of the transition if all molar absorption coefficients are the same and therefore the kinetics are first order:

$$\mathrm{p}(\mathbf{ij}) = k_{\mathbf{i/j}} \cdot \Delta t = \frac{r_{\mathbf{i/j}}}{[\mathbf{i}]} \cdot \Delta t = \frac{\phi_{\mathbf{i/j}} \cdot I_0 \cdot \varepsilon_{\mathbf{i}} \cdot d \cdot [\mathbf{i}] \cdot \left(\frac{1-e^{-d \sum_{\mathbf{i}} \varepsilon_{\mathbf{i}} \cdot [\mathbf{i}]}}{d \sum_{\mathbf{i}} \varepsilon_{\mathbf{i}} \cdot [\mathbf{i}]}\right)}{[\mathbf{i}]} \cdot \Delta t \quad \text{(Supplementary Equation 22)}$$

Additionally we have to include the diagonal elements p(**ii**) describing the probability that no conversion occurs:

$$\mathrm{p}(\mathbf{ii}) = 1 - \textstyle\sum_{\mathbf{j}} \mathrm{p}(\mathbf{ij}) \quad \text{(Supplementary Equation 23)}$$

Therefore, a Markov matrix can be written for hemithioindigo **1** in which the transition probabilities p(**ij**) are given:

$$M_2 = \begin{array}{c} \\ \\ \end{array} \begin{array}{cccc} \mathbf{A} & \mathbf{B} & \mathbf{C} & \mathbf{D} \end{array}$$

$$M_2 = \left(\begin{array}{cccc} \mathrm{p}(\mathbf{AA}) & \mathrm{p}(\mathbf{AB}) & \mathrm{p}(\mathbf{AC}) & \mathrm{p}(\mathbf{AD}) \\ \mathrm{p}(\mathbf{BA}) & \mathrm{p}(\mathbf{BB}) & \mathrm{p}(\mathbf{BC}) & \mathrm{p}(\mathbf{BD}) \\ \mathrm{p}(\mathbf{CA}) & \mathrm{p}(\mathbf{CB}) & \mathrm{p}(\mathbf{CC}) & \mathrm{p}(\mathbf{CD}) \\ \mathrm{p}(\mathbf{DA}) & \mathrm{p}(\mathbf{DB}) & \mathrm{p}(\mathbf{DC}) & \mathrm{p}(\mathbf{DD}) \end{array} \right) \begin{array}{c} \mathbf{A} \\ \mathbf{B} \\ \mathbf{C} \\ \mathbf{D} \end{array} \quad \text{(Supplementary Equation 24)}$$

By plotting the relative isomer percentage against the elapsed time during irradiation, four different kinetic plots (starting from either pure **A**, **B**, **C**, or **D**) were obtained from the experiments. These plots show the changes in isomer composition over time during the irradiation. These experimental kinetics were then simulated via an iterative process in which a Markov matrix M_2 (Supplementary Equation 24) is multiplied with the corresponding isomer percentage vector for each time point (Supplementary Equation 25). The

product vector is then multiplied by the same Markov matrix to give the corresponding vector of the next time point:

$$\begin{pmatrix} \%\mathbf{A} \\ \%\mathbf{B} \\ \%\mathbf{C} \\ \%\mathbf{D} \end{pmatrix}_{t_{x+1}} = \begin{pmatrix} p(\mathbf{AA}) & p(\mathbf{AB}) & p(\mathbf{AC}) & p(\mathbf{AD}) \\ p(\mathbf{BA}) & p(\mathbf{BB}) & p(\mathbf{BC}) & p(\mathbf{BD}) \\ p(\mathbf{CA}) & p(\mathbf{CB}) & p(\mathbf{CC}) & p(\mathbf{CD}) \\ p(\mathbf{DA}) & p(\mathbf{DB}) & p(\mathbf{DC}) & p(\mathbf{DD}) \end{pmatrix} \cdot \begin{pmatrix} \%\mathbf{A} \\ \%\mathbf{B} \\ \%\mathbf{C} \\ \%\mathbf{D} \end{pmatrix}_{t_x}$$

(Supplementary Equation 25)

The elements of the Markov matrix in Supplementary Equation 24 were adjusted manually until the best match with the experimental data was obtained.

We conducted four independent irradiation experiments for each solvent starting from either pure **A**, **B**, **C**, or **D**. If it is possible to keep I_0, d, and the initial concentration of the pure isomer [**i**] constant and the same in every experiment and if also the molar absorption coefficients of all different isomers are the same at the irradiation wavelength the same Markov matrix can be used to describe all experiments. In this case Supplementary Equation 22 transforms into:

$$p(\mathbf{ij}) = \frac{\phi_{i/j} \cdot I_0 \cdot \varepsilon_i \cdot d \cdot [\mathrm{i}] \cdot (\frac{1-e^{-d\sum_i \varepsilon_i \cdot [\mathrm{i}]}}{d\sum_i \varepsilon_i \cdot [\mathrm{i}]})}{[\mathrm{i}]} \cdot \Delta t = \phi_{i/j} \cdot I_0 \cdot \varepsilon_i \cdot d \cdot (\frac{1-e^{-d\sum_i \varepsilon_i \cdot [\mathrm{i}]}}{d\sum_i \varepsilon_i \cdot [\mathrm{i}]}) \cdot \Delta t = \phi_{i/j} \cdot const \cdot \Delta t$$

(Supplementary Equation 26)

and only depends on the individual quantum yields $\phi_{i/j}$.For benzene as solvent we were able to achieve a consistency high enough to be able to describe all four different isomers conversions with the same Markov matrix (see Supplementary Figure 20 - 23).

For all other solvents, different Markov matrices had to be used for each irradiation experiment of a different starting isomer **A**, **B**, **C**, or **D**. However, the relative ratios of off-diagonal elements in each row of the Markov matrix reproduce the relative ratios of the corresponding quantum yields ϕ according to Supplementary Equation 27 (exemplarily for the photoconversions of **A** to **B** and **C**):

$$\frac{p(\mathbf{AB})}{p(\mathbf{AC})} = \frac{\phi_{A/B} \cdot I_0 \cdot \varepsilon_A \cdot d \cdot (\frac{1-e^{-d\sum_i \varepsilon_i \cdot [\mathrm{i}]}}{d\sum_i \varepsilon_i \cdot [\mathrm{i}]}) \cdot \Delta t}{\phi_{A/C} \cdot I_0 \cdot \varepsilon_A \cdot d \cdot (\frac{1-e^{-d\sum_i \varepsilon_i \cdot [\mathrm{i}]}}{d\sum_i \varepsilon_i \cdot [\mathrm{i}]}) \cdot \Delta t} = \frac{\phi_{A/B}}{\phi_{A/C}}$$

(Supplementary Equation 27)

As can be seen from Supplementary Equation 26 the relative ratios of off-diagonal elements in each row of a Markov matrix directly correspond to the respective quantum yield ratios even if the molar absorption coefficients of different present isomers are different to each other (the sum expressions cancel each other out). As the matrix elements of one row correspond to one and the same experiment I_0, d, and the initial concentration of the pure isomer [**i**] are constant per se. Therefore, the relative ratios within one row of each Markov matrix have to be the same for all matrices.

Likewise the ratio of one off-diagonal probability to the sum of all off-diagonal probabilities within one row of the Markov matrix is related to the corresponding ratio of the quantum yields (exemplarily for the photoconversions of **A** to **B**, **C**, and **D**) giving the relative percentage of each process:

$$\text{rel. \%} = \frac{\mathrm{p}(\mathbf{AB})}{\mathrm{p}(\mathbf{AB})+\mathrm{p}(\mathbf{AC})+\mathrm{p}(\mathbf{AD})} = \frac{\phi_{\mathbf{A/B}}}{\phi_{\mathbf{A/B}}+\phi_{\mathbf{A/C}}+\phi_{\mathbf{A/D}}} \qquad \text{(Supplementary Equation 28)}$$

Additionally, the diagonal elements in the same Markov matrix can be compared at least qualitatively if the different molar absorptions coefficients ε_i are similar enough. This gives a qualitative assessment of the relative efficiencies of the overall photoconversions for each starting isomer. In the case of HTI **1** it is clear that isomers **A** and **B** are much more efficiently photoconverted than the isomers **C** and **D**, independent of the solvent.

All ratios determined from the irradiation experiments in different solvents are summarized in Supplementary Table 2.

In the solvent benzene-d_6 we could use the same Markov matrix for all four individual measurements (each corresponding to one individual row in the Markov matrix), which means that also ratios of quantum yields in different rows can be determined. The obtained ratios are in very good agreement with the ratios of the direct quantum yield measurements (see Supplementary Table 2). Absolute quantum yield values cannot be determined from the Markov matrix analysis since the exact values of I_0 and d are not known.

Calculated Ground State Energy Profile of Compound 1

A relaxed optimization of the four isomeric states and all four transition state structures of compound **1** at the B3LYP level of theory with the 6-311G(d,p) basis set has been conducted using the Gaussian09 Revision A.02 program package.[3] To account for solvent effects, the calculations have been carried out using the Polarizable Continuum Model (PCM) with dichloromethane parameters. The convergence criteria have been set tight and an ultrafine integration grid has been used. A following frequency analysis confirmed all four structures **A-1** (*Z*-(*S*)-(R_a)), **B-1** (*Z*-(*S*)-(S_a)), **C-1** (*E*-(*S*)-(R_a)), and **D-1** (*E*-(*S*)-(S_a)) to be minimum structures since no imaginary frequencies have been found. All other structures were shown to be first order saddle points on the potential energy surface since only one imaginary vibrational mode has been found, which confirmed them to be transition state structures.

Supplementary Figures

Determination of constitution and conformation in the crystalline state and in solution

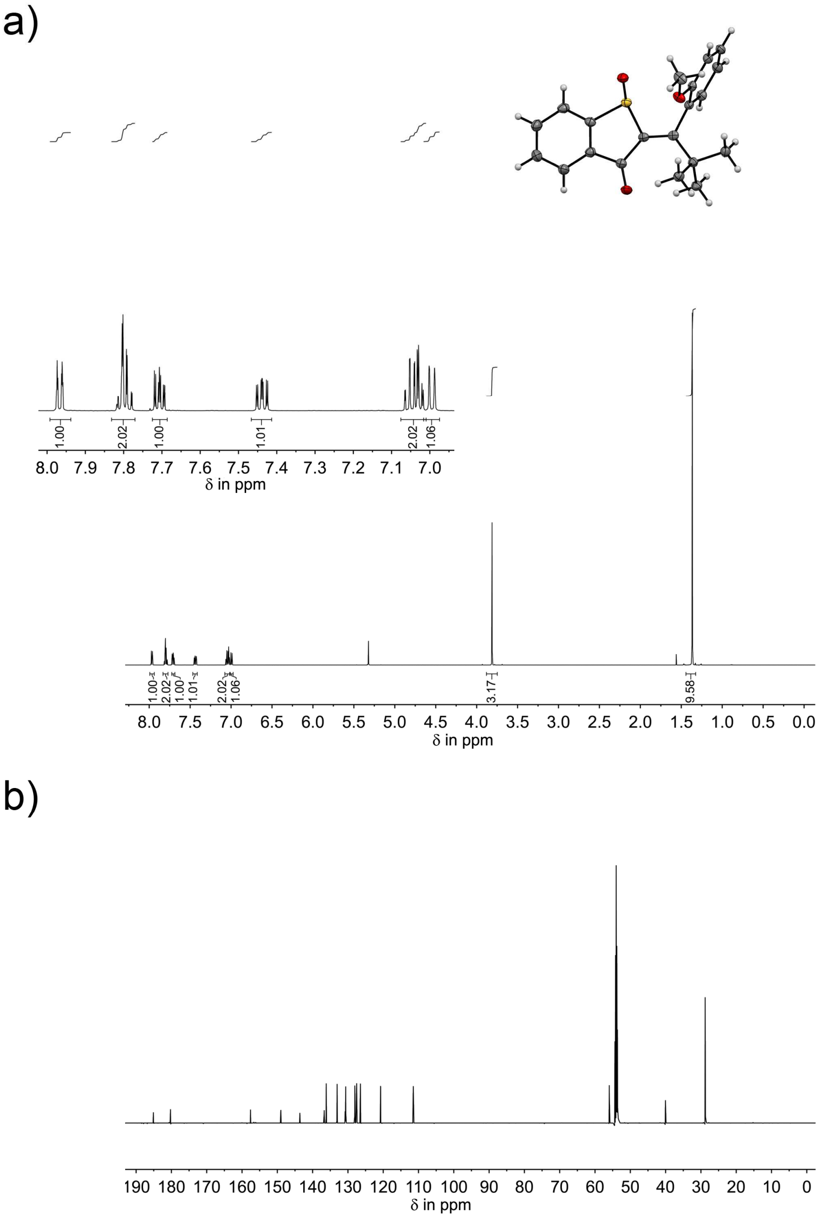

Supplementary Figure 1 | Structure assignment of A-1. a) Crystal structure of **A-1** and the corresponding ^{1}H NMR spectrum (CD_2Cl_2, 600 MHz, 27 °C) of the same crystal batch. The aromatic part of the spectrum is enlarged. The signals of only one single species are observed in the ^{1}H NMR spectrum, which could thus be directly assigned to the **A-1**. b) Corresponding ^{13}C NMR spectrum (CD_2Cl_2, 150 MHz, 27 °C).

a)

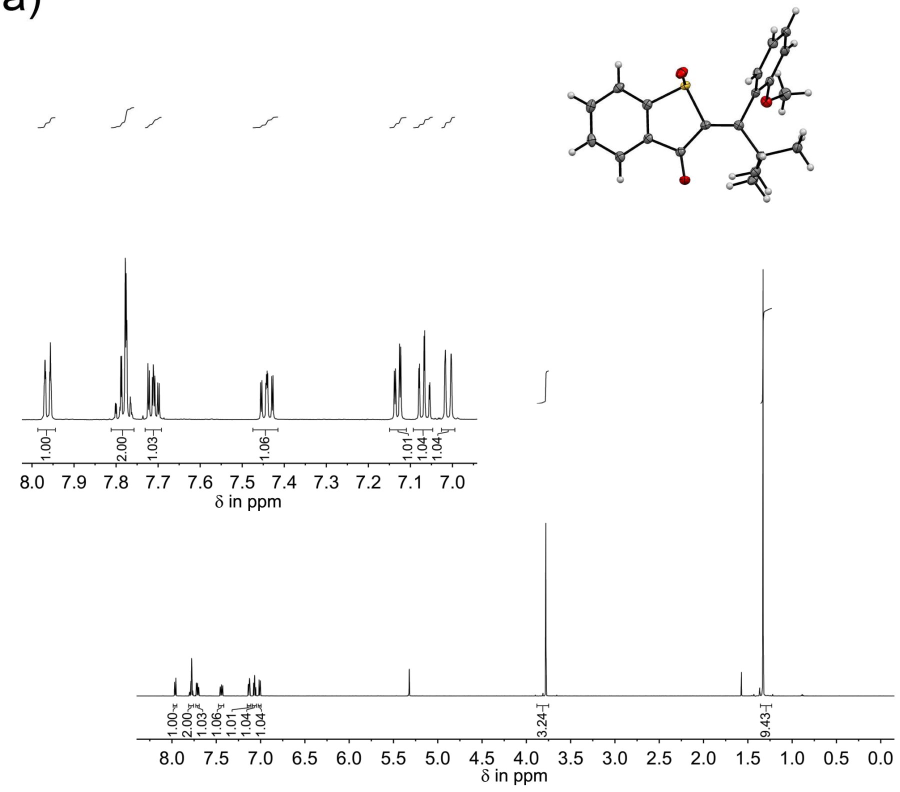

b)

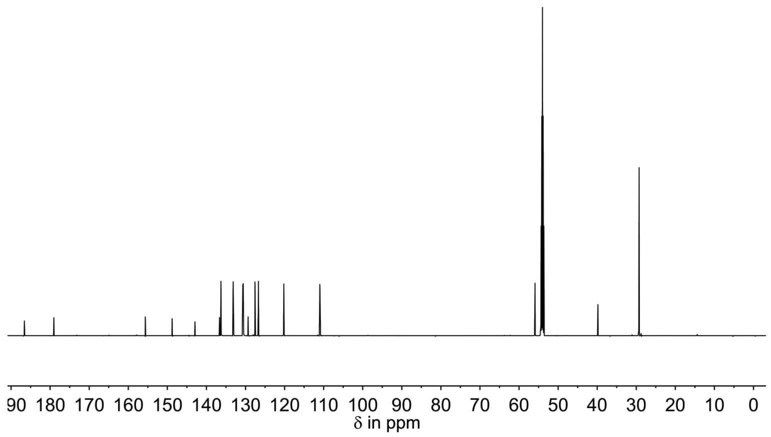

Supplementary Figure 2 | Structure assignment of B-1. a) Crystal structure of **B-1** and the corresponding ^{1}H NMR spectrum (CD_2Cl_2, 600 MHz, 27 °C) of the same crystal batch. The aromatic part of the spectrum is enlarged. The signals of only one single species are observed in the ^{1}H NMR spectrum, which could thus be directly assigned to the **B-1**. b) Corresponding ^{13}C NMR spectrum (CD_2Cl_2, 150 MHz, 27 °C).

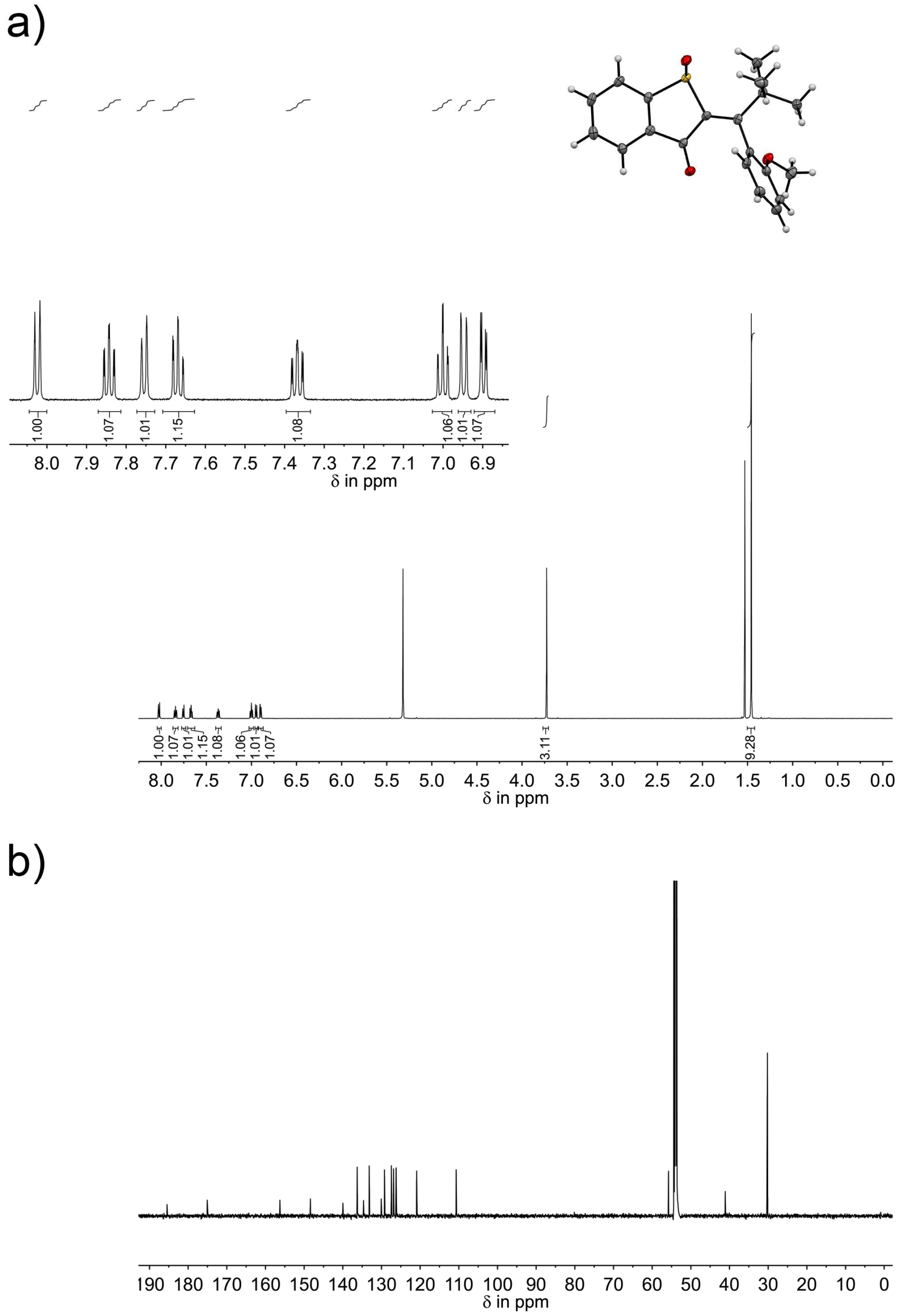

Supplementary Figure 3 | Structure assignment of C-1. a) Crystal structure of **C-1** and the corresponding ^{1}H NMR spectrum (CD_2Cl_2, 600 MHz, 27 °C) of the same crystal batch. The aromatic part of the spectrum is enlarged. The signals of only one single species are observed in the ^{1}H NMR spectrum, which could thus be directly assigned to the **C-1**. b) Corresponding ^{13}C NMR spectrum (CD_2Cl_2, 150 MHz, 27 °C).

a)

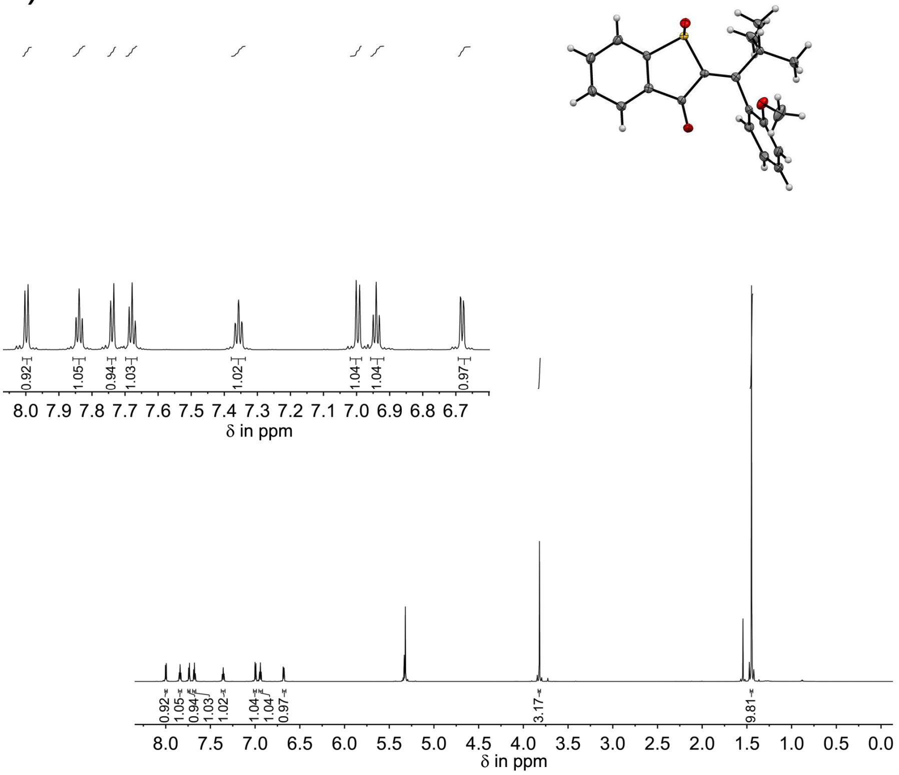

b)

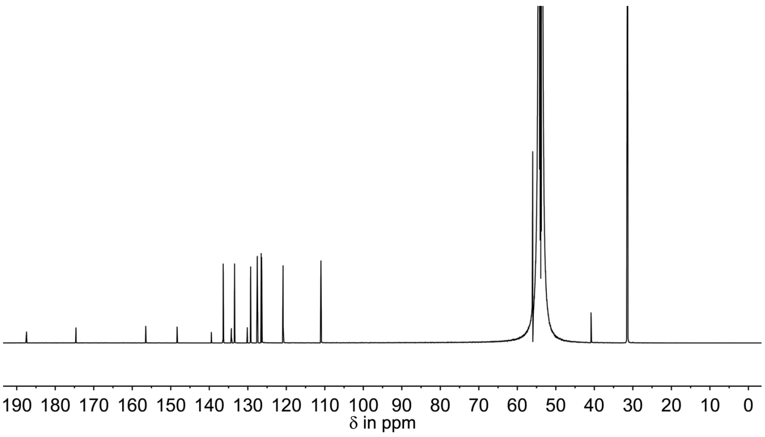

Supplementary Figure 4 | Structure assignment of D-1. a) Crystal structure of **D-1** and the corresponding ^{1}H NMR spectrum (CD_2Cl_2, 800 MHz, 25 °C) of the same crystal batch. The aromatic part of the spectrum is enlarged. The signals of only one single species are observed in the ^{1}H NMR spectrum, which could thus be directly assigned to the **D-1**. b) Corresponding ^{13}C NMR spectrum (CD_2Cl_2, 200 MHz, 25 °C).

Thermal atropisomerizations

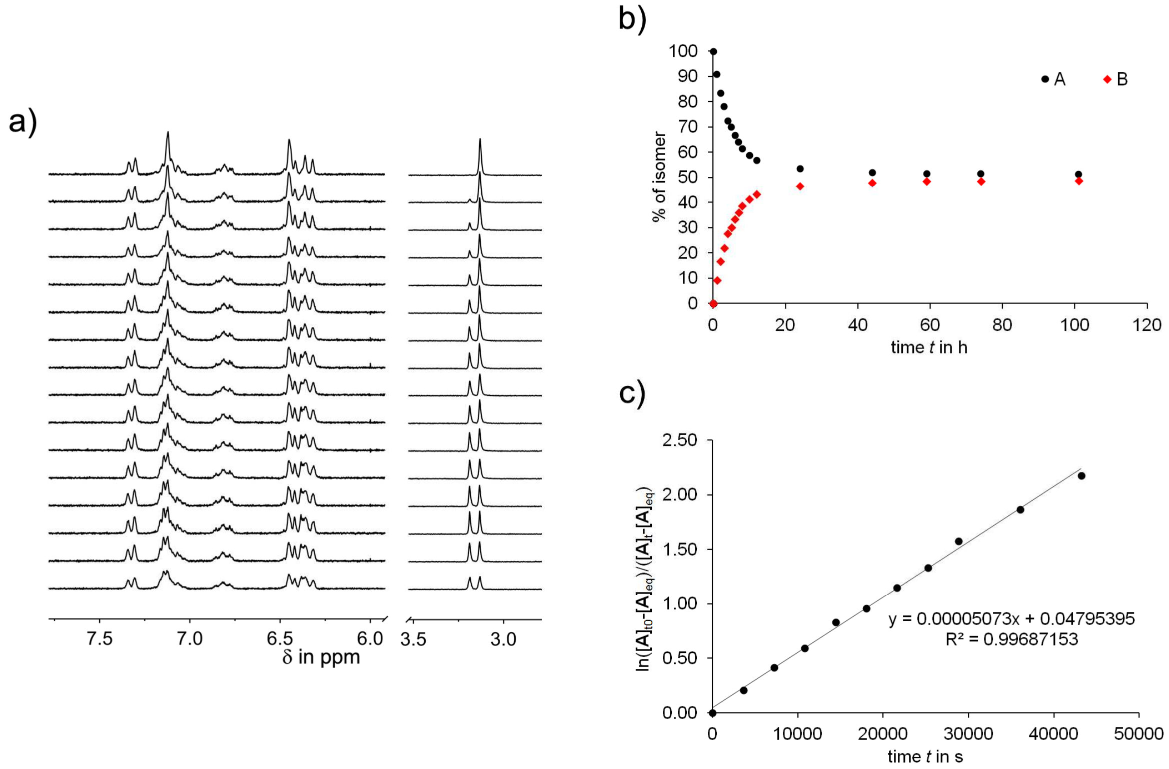

Supplementary Figure 5 | Thermal atropisomerization of A to B. a) Thermal atropisomerization of **A** to **B** in $(CDCl_2)_2$ at 82 °C followed by ^1H NMR spectroscopy (200 MHz, 25 °C) in regular time intervals . b) Atropisomer conversion over time. c) First order kinetic analysis of the thermal atropisomerization of **A** to **B**. Taking into account the dynamic equilibrium by plotting according to Supplementary Equation 1 gives a linear relationship. The slope *m* can be translated into the rate constants $k_{(\mathbf{A/B})}$ according to Supplementary Equation 3. The corresponding Gibbs energy of activation for the thermal **A** to **B** isomerization are given in Supplementary Table 1.

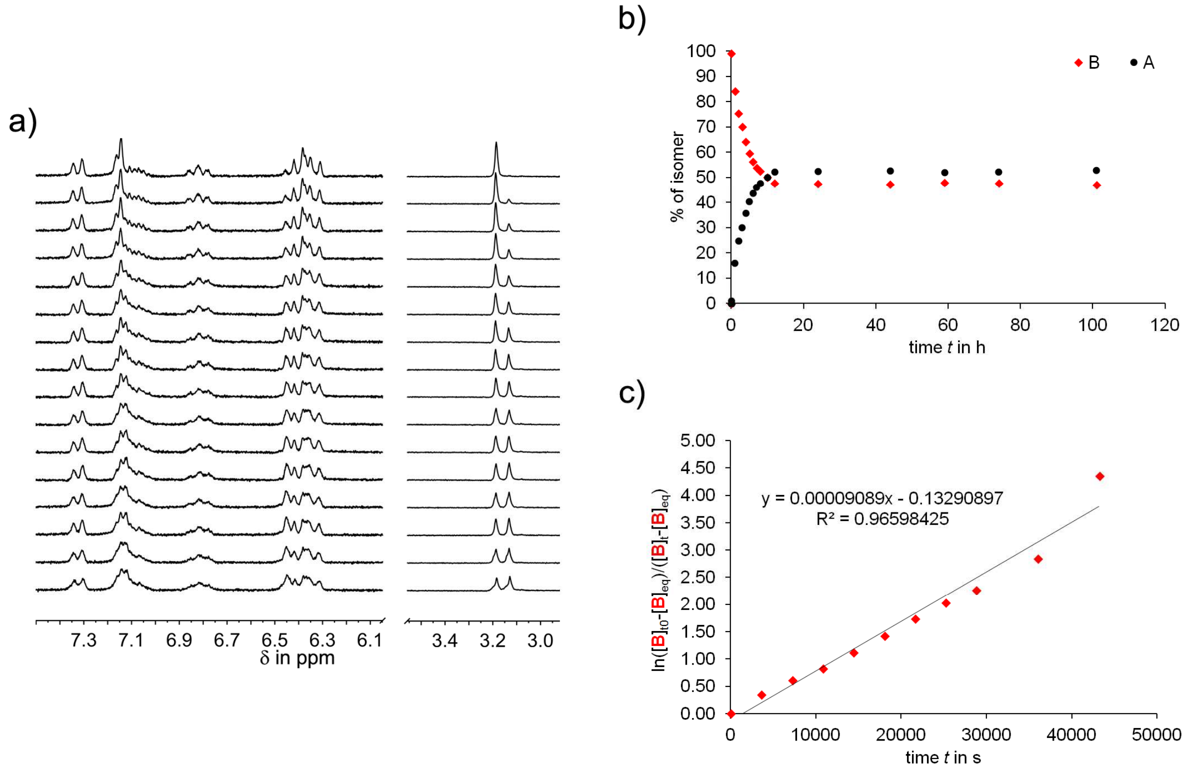

Supplementary Figure 6 | Thermal atropisomerization of B to A. a) Thermal atropisomerization of **B** to **A** in $(CDCl_2)_2$ at 82 °C followed by ^{1}H NMR spectroscopy (200 MHz, 25 °C) in regular time intervals. b) Atropisomer conversion over time. c) First order kinetic analysis of the thermal atropisomerization of **B** to **A**. Taking into account the dynamic equilibrium by plotting according to Supplementary Equation 7 gives a linear relationship. The slope *m* can be translated into the rate constants $k_{(\mathbf{B/A})}$ according to Supplementary Equation 9. The corresponding Gibbs energy of activation for the thermal **B** to **A** isomerization are given in Supplementary table 1.

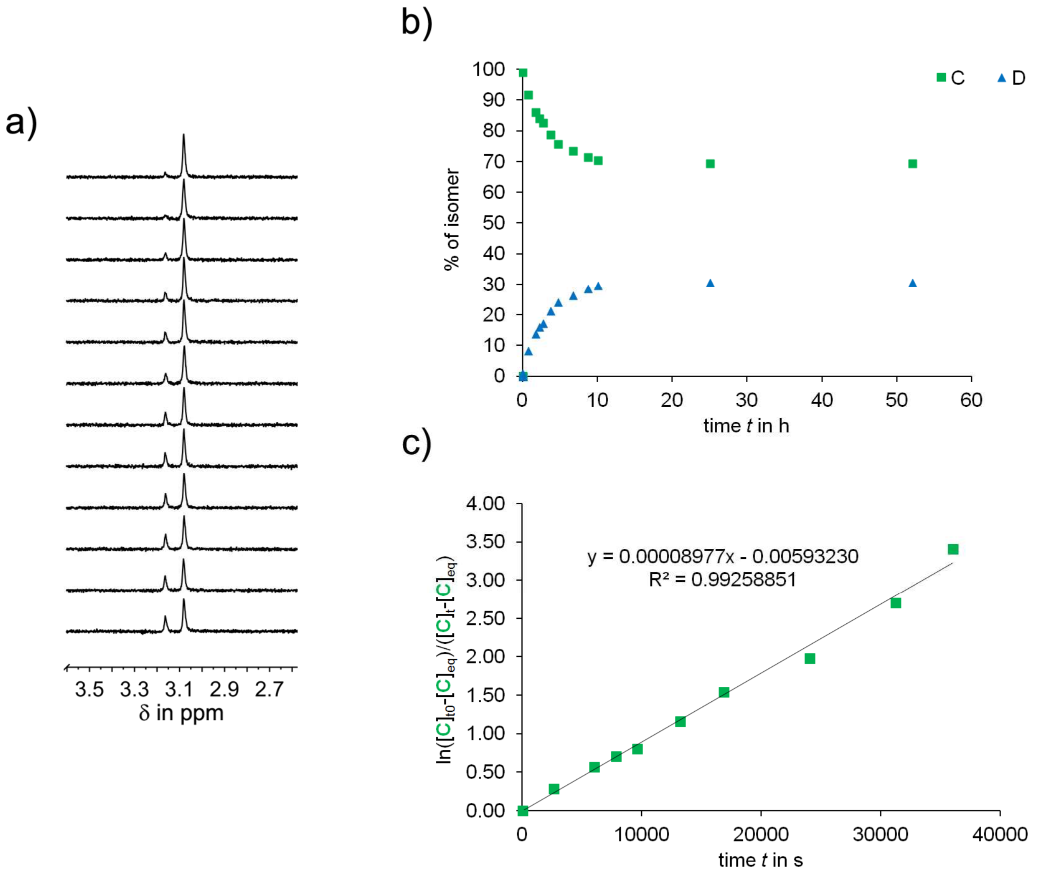

Supplementary Figure 7 | Thermal atropisomerization of C to D. a) Thermal atropisomerization of **C** to **D** in $(CDCl_2)_2$ at 100 °C followed by ^{1}H NMR spectroscopy (200 MHz, 25 °C) in regular time intervals. b) Atropisomer conversion over time. c) First order kinetic analysis of the thermal atropisomerization of **C** to **D**. Taking into account the dynamic equilibrium by plotting according to Supplementary Equation 2 gives a linear relationship. The slope *m* can be translated into the rate constants $k_{(\mathbf{C/D})}$ according to Supplementary Equation 4. The corresponding Gibbs energy of activation for the thermal **C** to **D** isomerization are given in Supplementary Table 1.

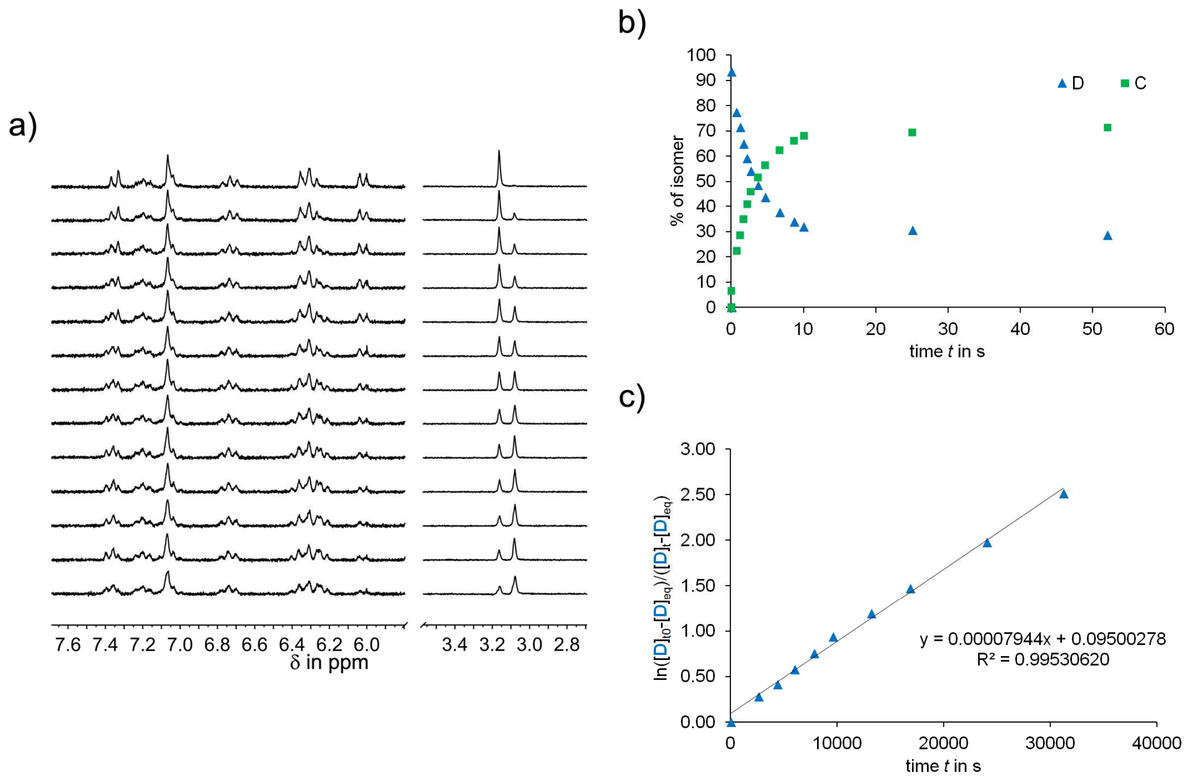

Supplementary Figure 8 | Thermal atropisomerization of D to C. a) Thermal atropisomerization of **D** to **C** in $(CDCl_2)_2$ at 100 °C followed by ^{1}H NMR spectroscopy (200 MHz, 25 °C) in regular time intervals. b) Atropisomer conversion over time. c) First order kinetic analysis of the thermal atropisomerization of **D** to **C**. Taking into account the dynamic equilibrium by plotting according to Supplementary Equation 8 gives a linear relationship. The slope *m* can be translated into the rate constants $k_{(\mathbf{D/C})}$ according to Supplementary Equation 10. The corresponding Gibbs energy of activation for the thermal **D** to **C** isomerization are given in Supplementary Table 1.

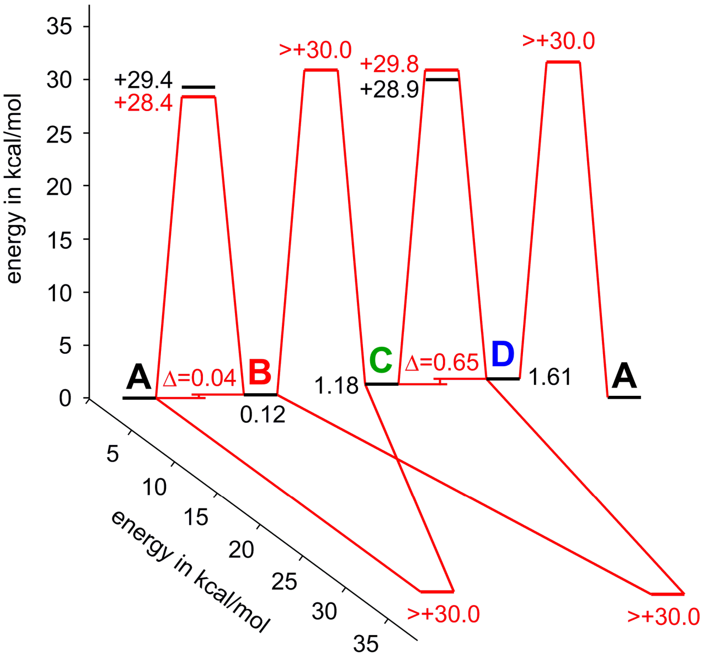

Supplementary Figure 9 | Ground state energy profile for 1. Thermal interconversion between the four different isomers **A** to **D** at ambient temperature is completely prevented by very high kinetic barriers. Black values are derived from quantum chemical calculations (B3LYP/6-311G(d,p)), red values were determined experimentally. The Δ values correspond to the experimentally determined $\Delta\Delta G^0$ values.

Molar absorption coefficients at room temperature

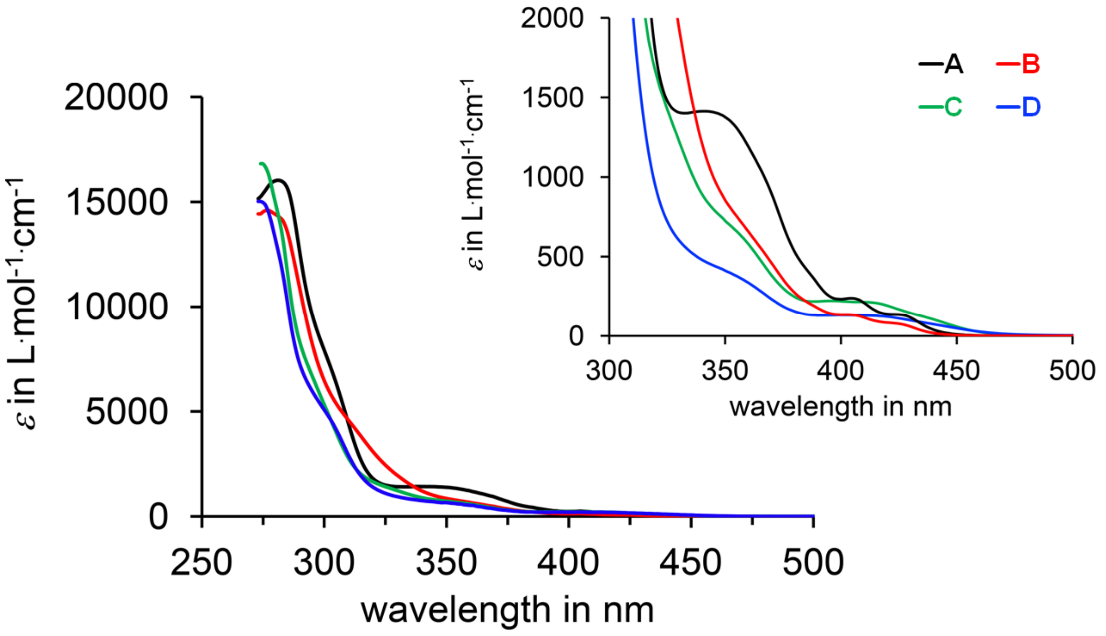

Supplementary Figure 10 | Molar absorption coefficients of 1. Molar absorption coefficients ε of **A** (black), **B** (red), **C** (green), and **D** (blue) at 23 °C in benzene solution.

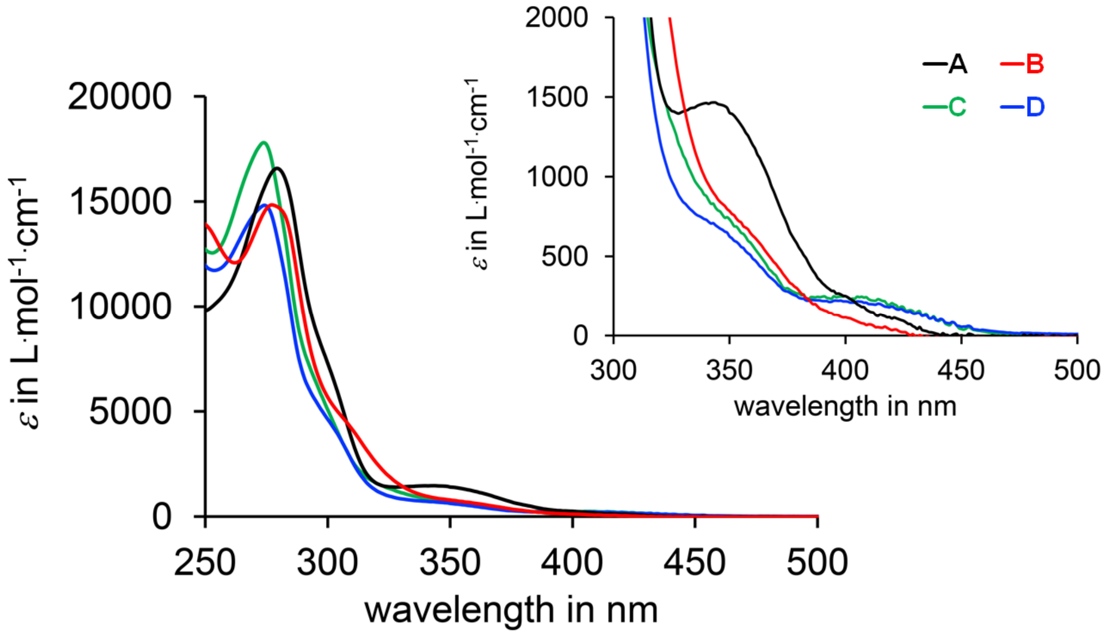

Supplementary Figure 11 | Molar absorption coefficients of 1. Molar absorption coefficients ε of **A** (black), **B** (red), **C** (green), and **D** (blue) at 23 °C in CH_2Cl_2 solution.

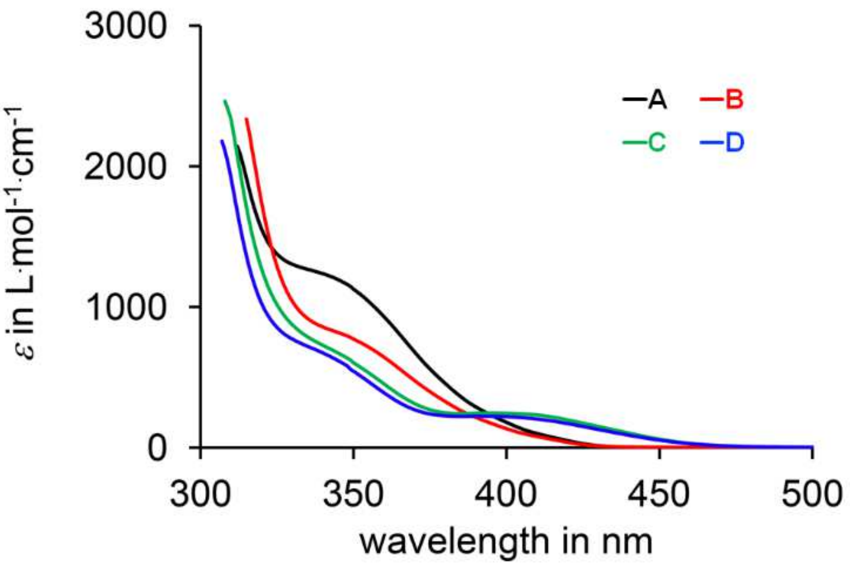

Supplementary Figure 12 | Molar absorption coefficients of 1. Molar absorption coefficients ε of **A** (black), **B** (red), **C** (green), and **D** (blue) at 23 °C in MeOH solution.

Molar absorption coefficients in EPA glass at 90 K

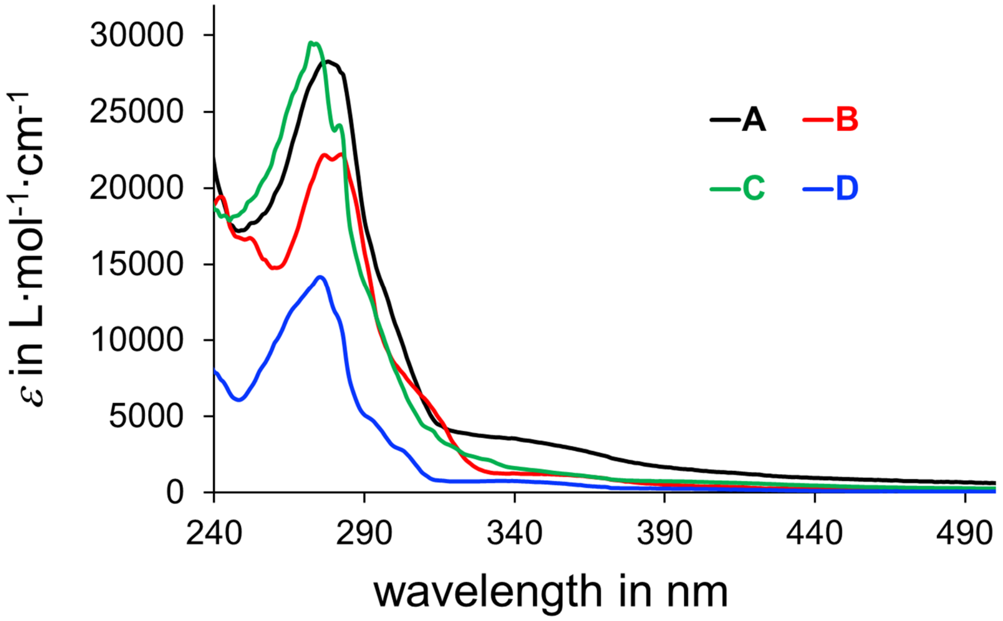

Supplementary Figure 13 | Molar absorption coefficients of 1. Molar absorption coefficients ε of **A** (black), **B** (red), **C** (green), and **D** (blue) in EPA glass (diethyl ether/*iso*pentane/ethanol 5:5:2) at 90 K.

Photoconversion of A, B, C, and D determined by quantum yield measurements

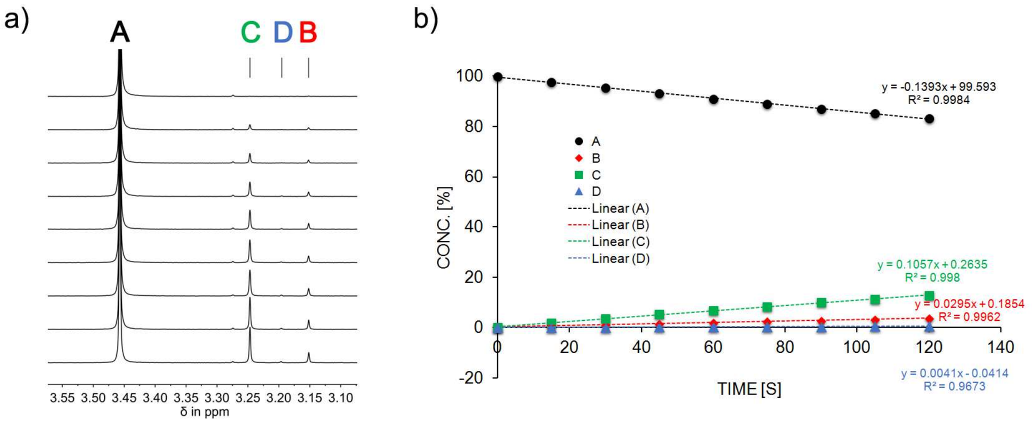

Supplementary Figure 14 | Quantum yields of A. Quantum yield ϕ measurement for the photoconversion of **A-1** at 23 °C in benzene-d_6 solution (3.04 mM) using a focused 405 nm LED. a) ^{1}H NMR spectra (400 MHz, 18 °C) recorded after different irradiation durations. Signals of individual isomers are indicated. b) The relative changes of the isomer composition is plotted against different duration times of irradiation. Each point represents an individual measurement, the relative isomer ratios were determined by ^{1}H NMR spectroscopy. Linear behavior is observed showing that only **A-1** undergoes photoreactions. Quantum yields were determined by averaging over all experiments (see Supplementary Table 2).

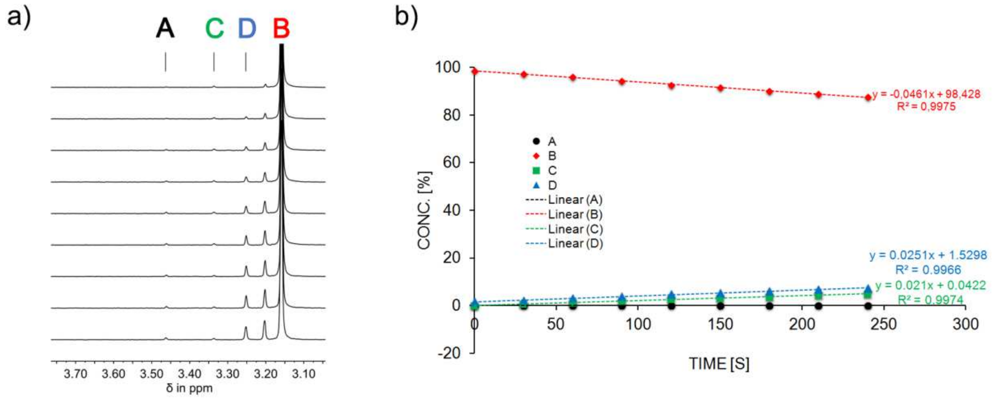

Supplementary Figure 15 | Quantum yields of B. Quantum yield ϕ measurement for the photoconversion of **B-1** at 23 °C in benzene-d_6 solution (3.04 mM) using a focused 405 nm LED. a) ^{1}H NMR spectra (400 MHz, 22 °C) recorded after different irradiation durations. Signals of individual isomers are indicated. b) The relative changes of the isomer composition is plotted against different duration times of irradiation. Each point represents an individual measurement, the relative isomer ratios were determined by ^{1}H NMR spectroscopy. Linear behavior is observed showing that only **B-1** undergoes photoreactions. Quantum yields were determined by averaging over all experiments (see Supplementary Table 2).

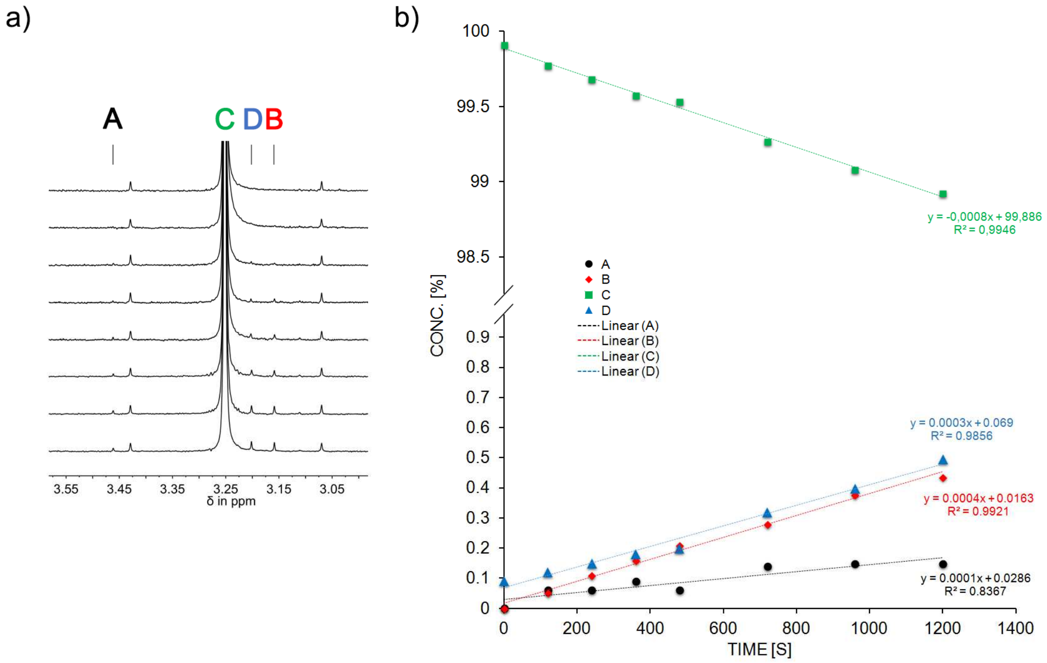

Supplementary Figure 16 | Quantum yields of C. Quantum yield ϕ measurement for the photoconversion of **C-1** at 23 °C in benzene-d_6 solution (3.04 mM) using a focused 405 nm LED. a) ^{1}H NMR spectra (400 MHz, 22 °C) recorded after different irradiation durations. Signals of individual isomers are indicated. b) The relative changes of the isomer composition is plotted against different duration times of irradiation. Each point represents an individual measurement, the relative isomer ratios were determined by ^{1}H NMR spectroscopy. Linear behavior is observed showing that only **C-1** undergoes photoreactions. Quantum yields were determined by averaging over all experiments (see Supplementary Table 2).

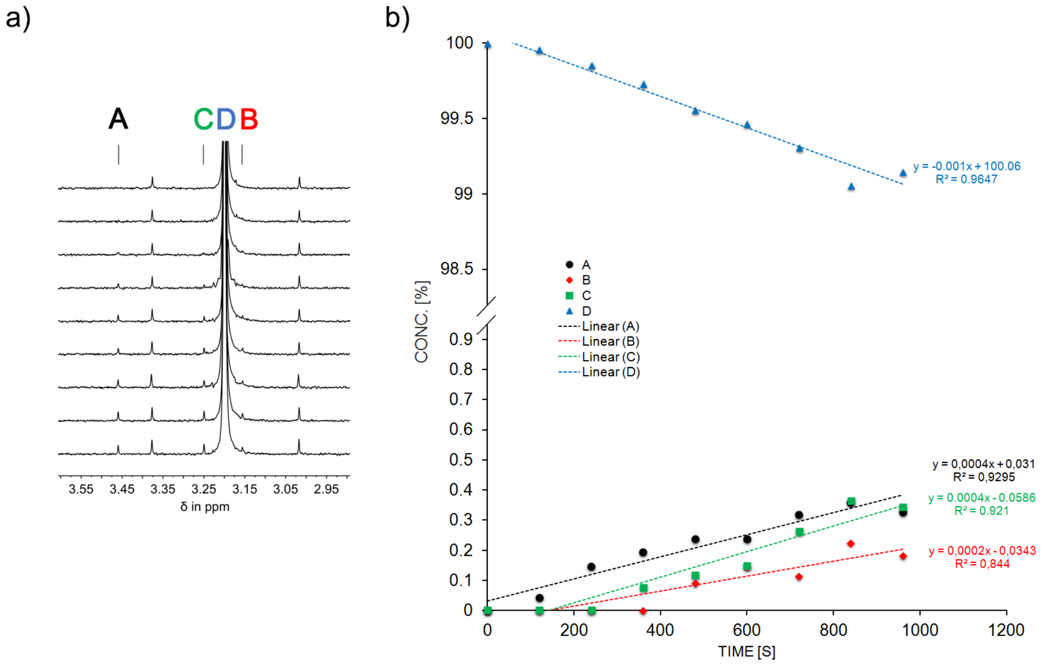

Supplementary Figure 17 | Quantum yields of D. Quantum yield ϕ measurement for the photoconversion of **D-1** at 23 °C in benzene-d_6 solution (3.04 mM) using a 405 nm LED. a) ^{1}H NMR spectra (400 MHz, 20 °C) recorded after different irradiation durations. Signals of individual isomers are indicated. b) The relative changes of the isomer composition is plotted against different duration times of irradiation. Each point represents an individual measurement, the relative isomer ratios were determined by ^{1}H NMR spectroscopy. Quantum yields were determined by averaging over all experiments (see Supplementary Table 2).

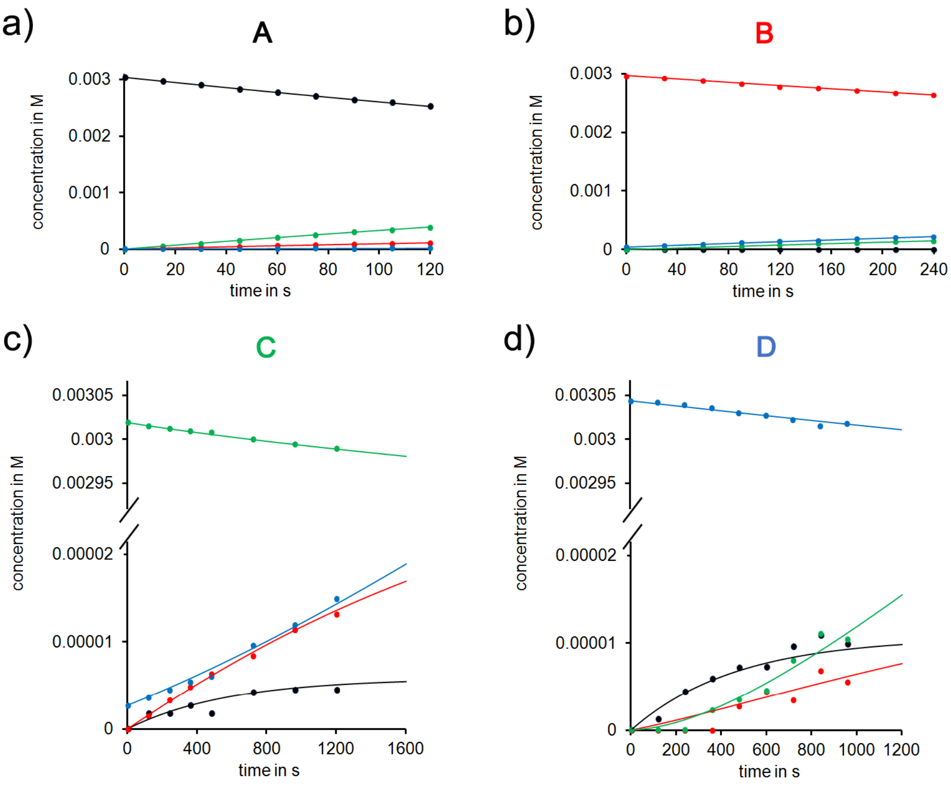

e)

	A	B	C	D	
	-	ϕ(AB)	ϕ(AC)	ϕ(AD)	A
	ϕ(BA)	-	ϕ(BC)	ϕ(BD)	B
	ϕ(CA)	ϕ(CB)	-	ϕ(CD)	C
	ϕDA)	ϕ(DB)	ϕ(DC)	-	D

⇒

	A	B	C	D	
	-	9.025	30.750	0.840	A
	0.900	-	7.520	9.300	B
	0.087	0.117	-	0.070	C
	0.261	0.058	0.012	-	D

Supplementary Figure 18 | Quantum yields of 1. Quantum yield ϕ measurements for the photoconversions of **1** at 23 °C in benzene-d_6 solution. a) Photoconversion of **A**. b) Photoconversion of **B**. c) Photoconversion of **C**. d) Photoconversion of **D**. e) Optimized fitted quantum yields for all photoconversion processes.

Markov matrix analysis of the photoconversion of A, B, C, and D in different solvents

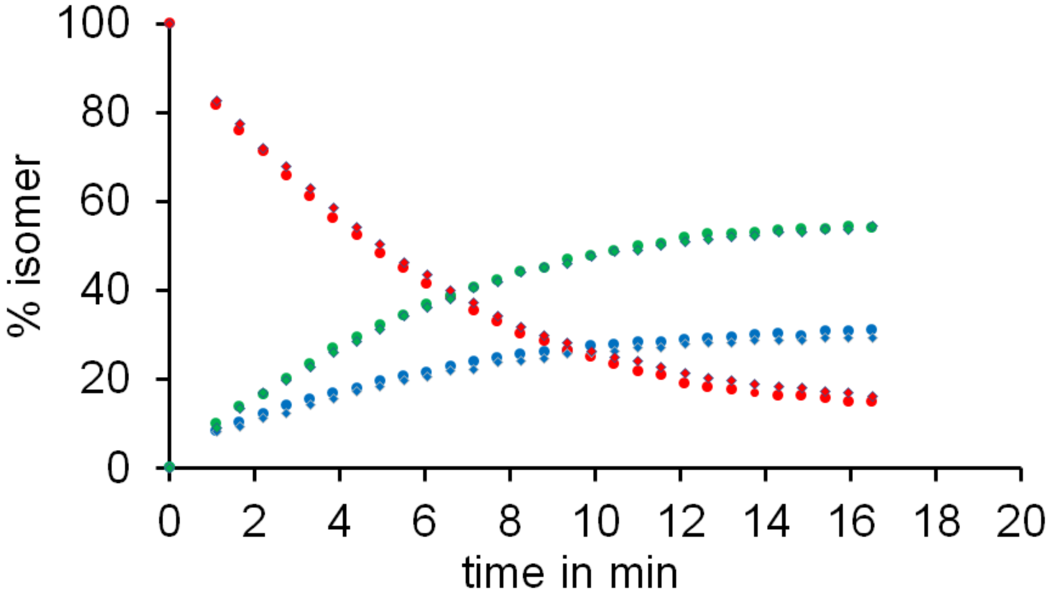

Supplementary Figure 19 | Accuracy of Markov matrix analysis. Changes in the relative concentrations of isomers **A**, **B**, **C**, and **D** during illumination of isomer **B** in tetrachloroethane-d_2 solution at 27 °C with 405 nm light. Data points were obtained via integration of indicative signals from ^{1}H NMR spectra (400 MHz) recorded during irradiation. Diamonds and circles represents two independent measurements using a freshly prepared solution of isomer **B** in a different NMR tube with newly attached optical fiber in each case. The deviation is commonly lower than 1% showing that all the external parameters I_0, d, and initial concentration of the pure isomer [**i**] were kept constant and in every independent experiment.

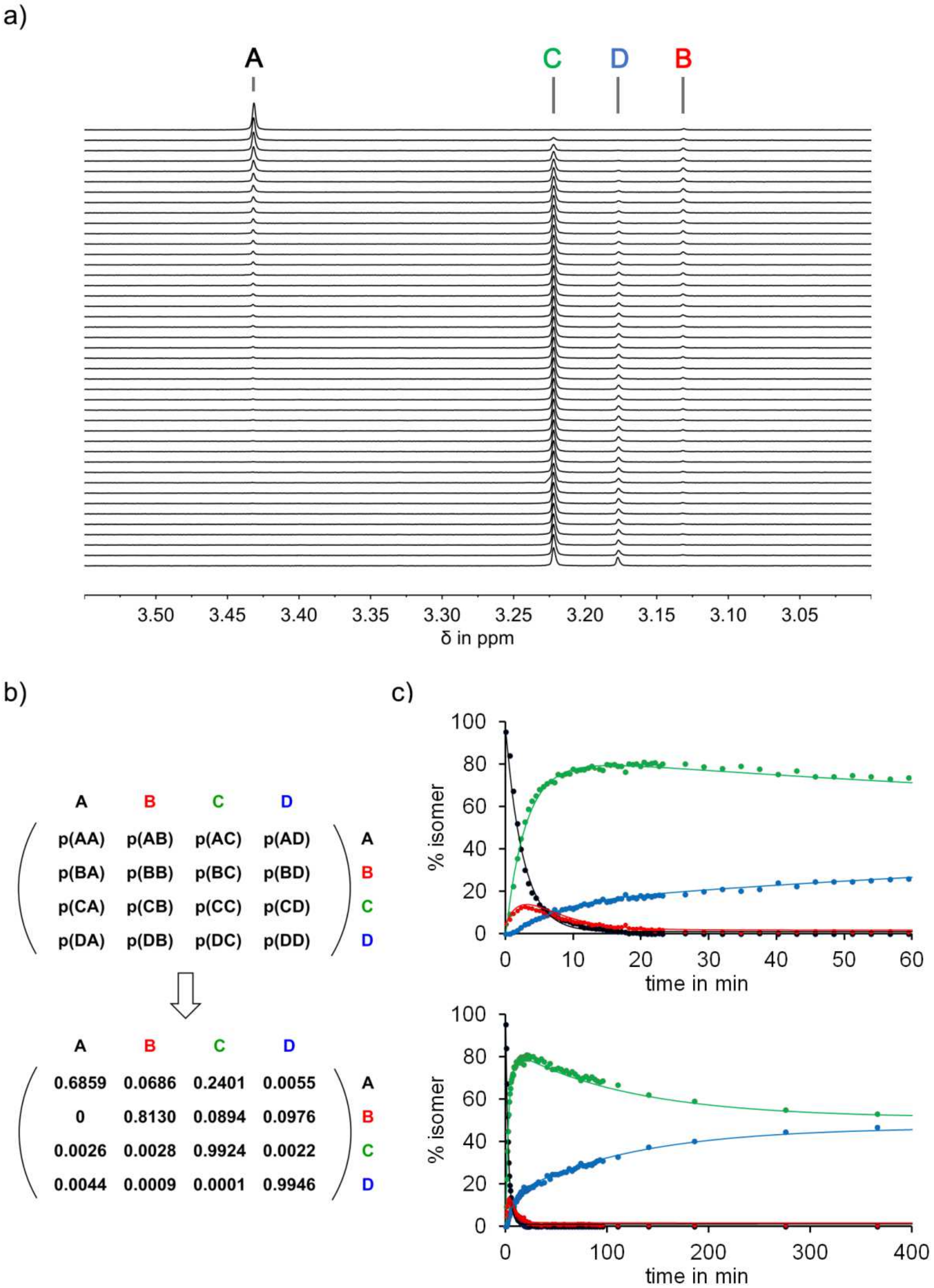

Supplementary Figure 20 | Markov matrix analysis of A in benzene-*d*$_6$. Markov matrix analysis of the photoreactions of **1** starting from isomer **A** in benzene-*d*$_6$ solution at 27 °C under 405 nm illumination. a) Section of the ^{1}H NMR spectra (400 MHz) recorded during irradiation of **A-1**. Spectra were recorded in 33 s intervals. The bottom spectrum was recorded after 365 min. b) Markov matrix describing the different phototransition probabilities p(**ij**) per minute for **1**. c) Kinetic plots of the experimentally determined changes in isomer composition during irradiation of **A-1** (dots) and fitted values derived from the Markov matrix (lines).

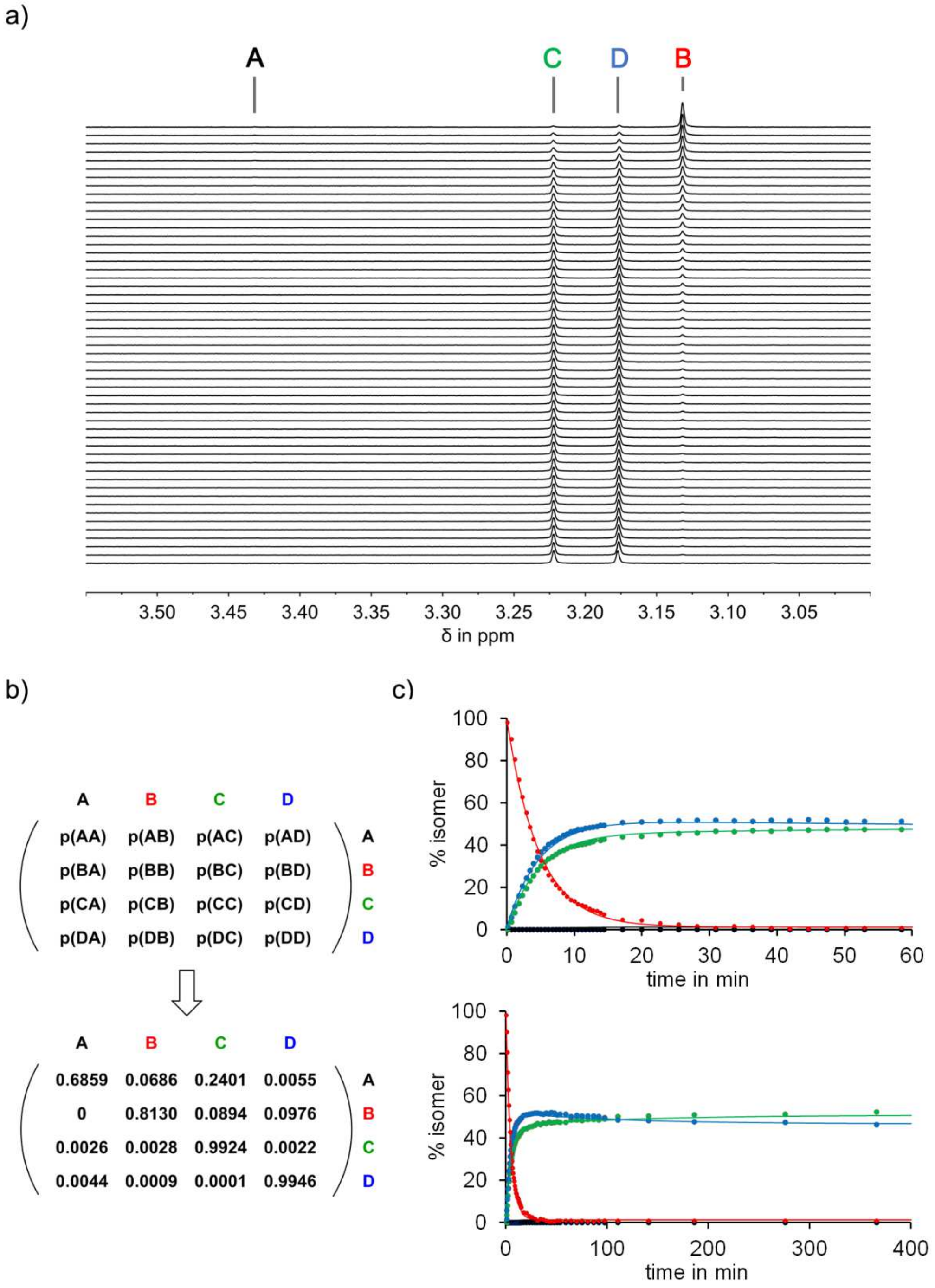

Supplementary Figure 21 | Markov matrix analysis of B in benzene-d_6. Markov matrix analysis of the photoreactions of **1** starting from isomer **B** in benzene-d_6 solution at 27 °C under 405 nm illumination. a) Section of the ^{1}H NMR spectra (400 MHz) recorded during irradiation of **B-1**. Spectra were recorded in 33 s intervals. The bottom spectrum was recorded after 365 min. b) Markov matrix describing the phototransition probabilities p(**ij**) per minute for **1**. c) Kinetic plots of the experimentally determined changes in isomer composition during irradiation of **B-1** (dots) and fitted values derived from the Markov matrix (lines).

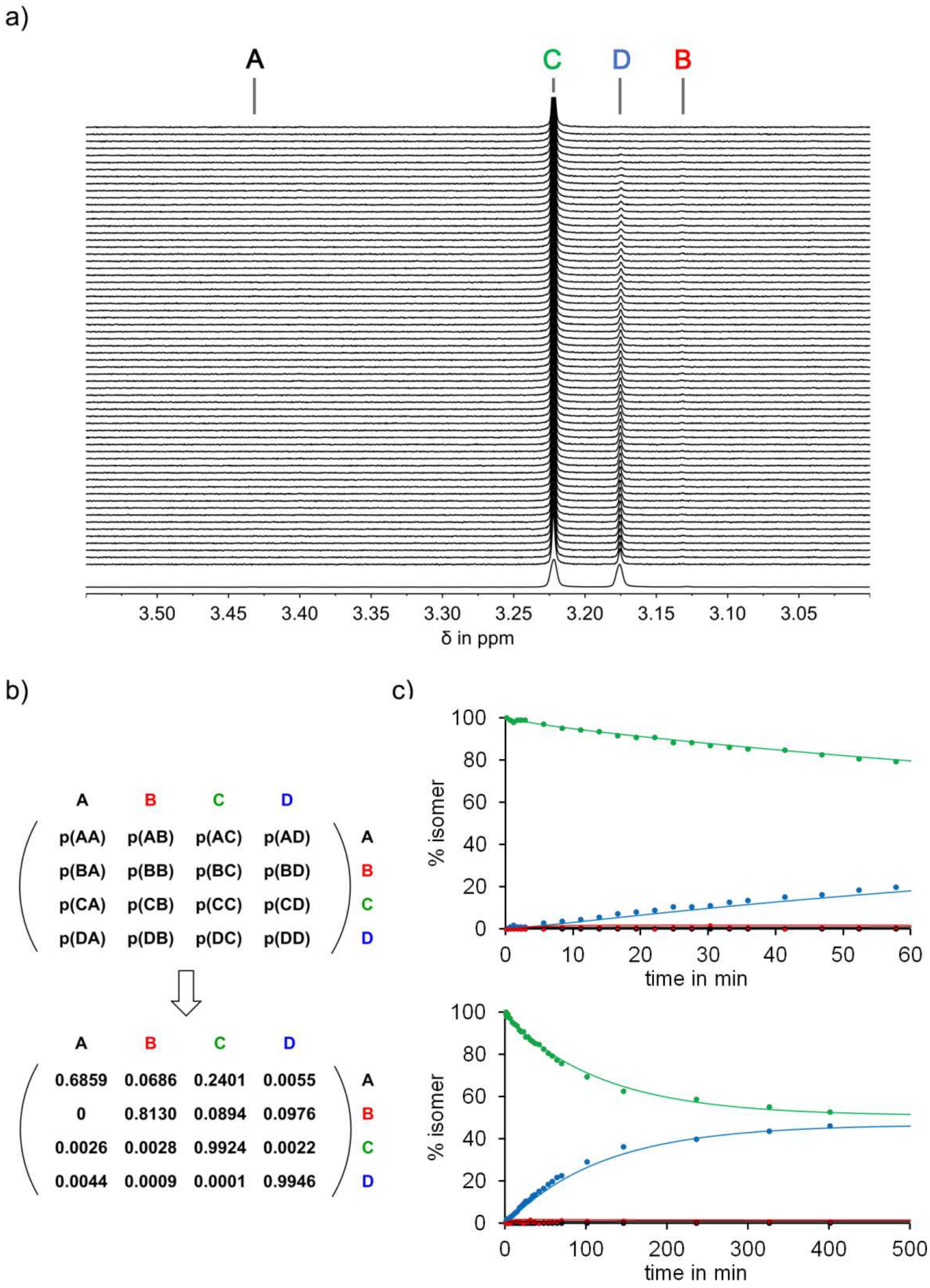

Supplementary Figure 22 | Markov matrix analysis of C in benzene-d_6. Markov matrix analysis of the photoreactions of **1** starting from isomer **C** in benzene-d_6 solution at 27 °C under 405 nm illumination. a) Section of the ^{1}H NMR spectra (400 MHz) recorded during irradiation of **C-1**. Spectra were recorded in 66 s intervals. The bottom spectrum was recorded after 12 h. b) Markov matrix describing the phototransition probabilities p(**ij**) per minute for **1**. c) Kinetic plots of the experimentally determined changes in isomer composition during irradiation of **C-1** (dots) and fitted values derived from the Markov matrix (lines).

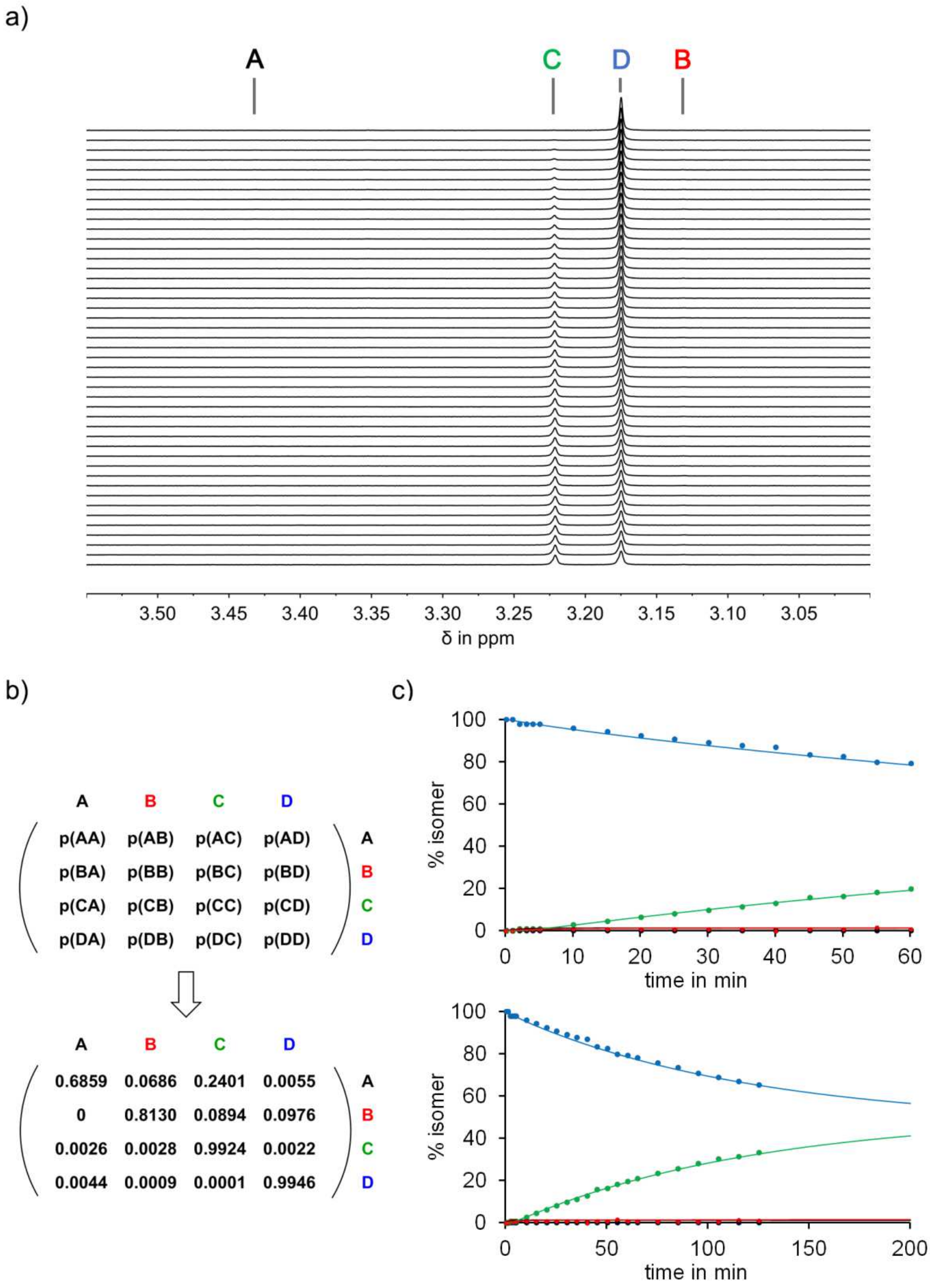

Supplementary Figure 23 | Markov matrix analysis of D in benzene-d_6. Markov matrix analysis of the photoreactions of **1** starting from isomer **D** in benzene-d_6 solution at 27 °C under 405 nm illumination. a) Section of the ^{1}H NMR spectra (400 MHz) recorded during irradiation of **D-1**. Spectra were recorded in 132 s intervals. b) Markov matrix describing the phototransition probabilities p(**ij**) per minute for **1**. c) Kinetic plots of the experimentally determined changes in isomer composition during irradiation of **D-1** (dots) and fitted values derived from the Markov matrix (lines).

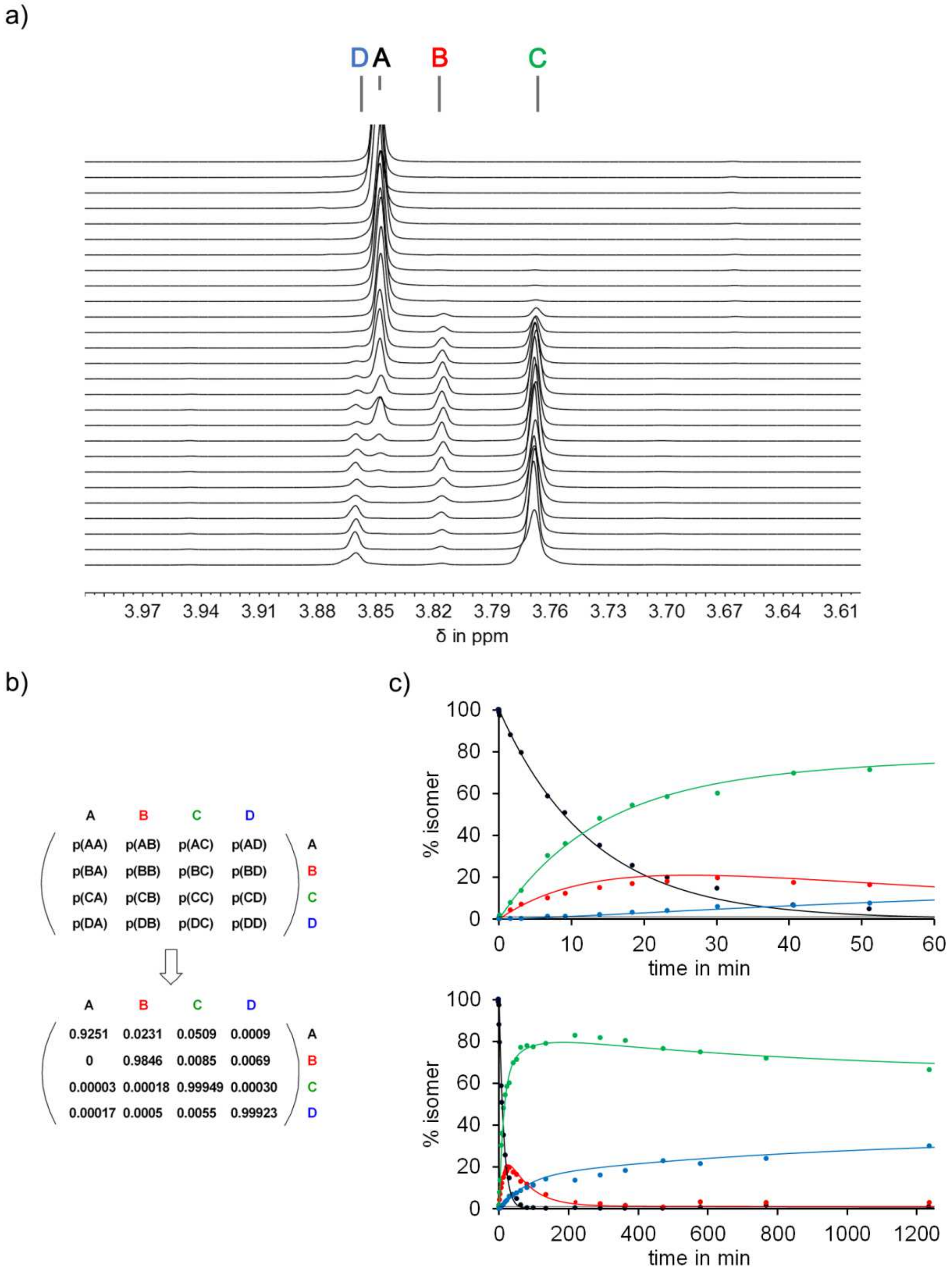

Supplementary Figure 24 | Markov matrix analysis of A in CD_2Cl_2. Markov matrix analysis of the photoreactions of **1** starting from isomer **A** in CD_2Cl_2 solution at 22 °C under 405 nm illumination. a) Section of the ^{1}H NMR spectra (400 MHz) recorded during irradiation of **A-1**. Each spectrum represents a set of data points in the kinetic plot. b) Markov matrix describing the phototransition probabilities p(**ij**) per minute for **1**. c) Kinetic plots of the experimentally determined changes in isomer composition during irradiation of **A-1** (dots) and fitted values derived from the Markov matrix (lines).

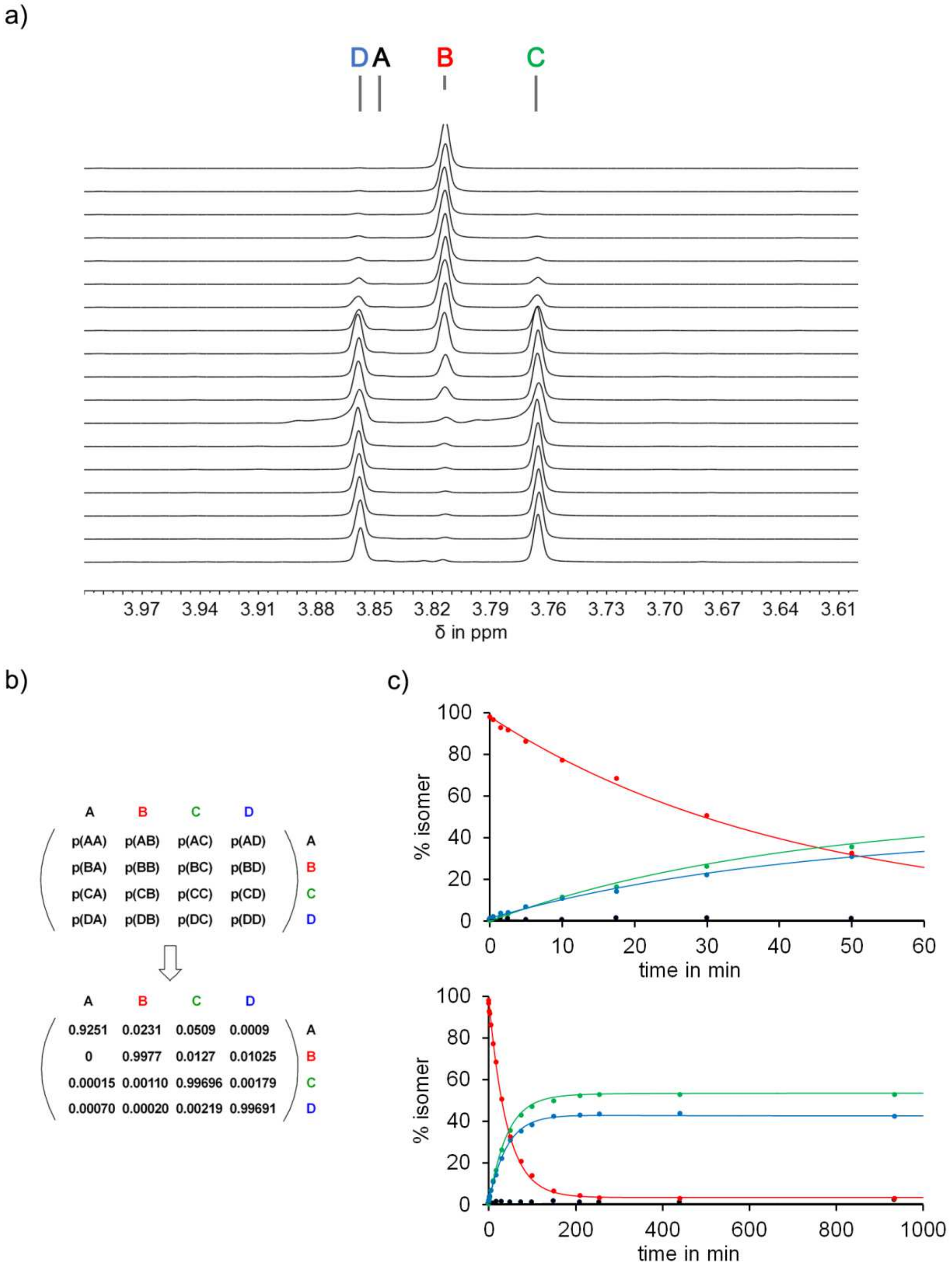

Supplementary Figure 25 | Markov matrix analysis of B in CD_2Cl_2. Markov matrix analysis of the photoreactions of **1** starting from isomer **B** in CD_2Cl_2 solution at 22 °C under 405 nm illumination. a) Section of the ^{1}H NMR spectra (400 MHz) recorded during irradiation of **B**-**1**. Each spectrum represents a set of data points in the kinetic plot. b) Markov matrix describing the phototransition probabilities p(**ij**) per minute for **1**. c) Kinetic plots of the experimentally determined changes in isomer composition during irradiation of **B**-**1** (dots) and fitted values derived from the Markov matrix (lines).

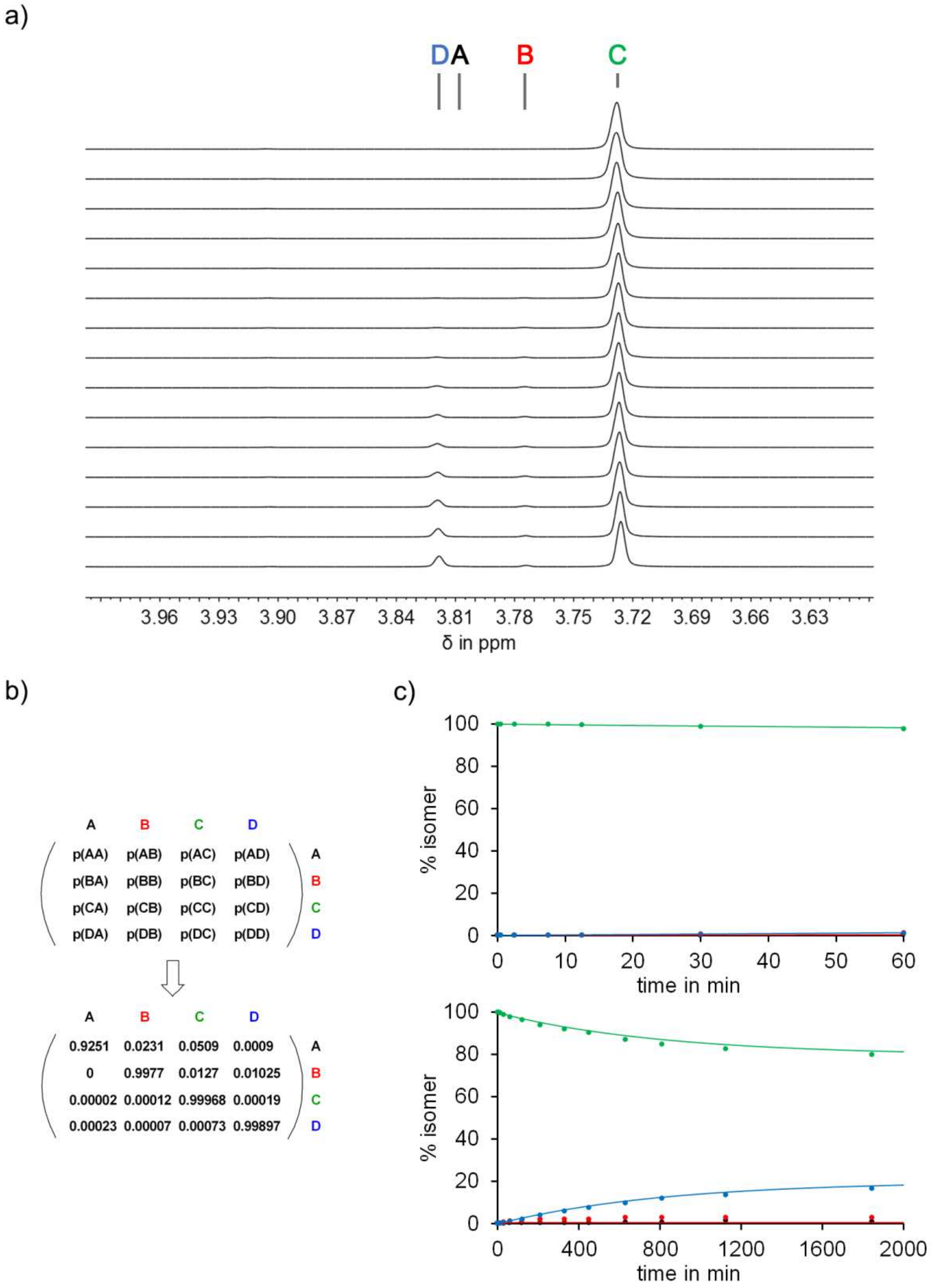

Supplementary Figure 26 | Markov matrix analysis of C in CD_2Cl_2. Markov matrix analysis of the photoreactions of **1** starting from isomer **C** in CD_2Cl_2 solution at 22 °C under 405 nm illumination. a) Section of the ^{1}H NMR spectra (400 MHz) recorded during irradiation of **C-1**. Each spectrum represents a set of data points in the kinetic plot. b) Markov matrix describing the phototransition probabilities p(**ij**) per minute for **1**. c) Kinetic plots of the experimentally determined changes in isomer composition during irradiation of **C-1** (dots) and fitted values derived from the Markov matrix (lines).

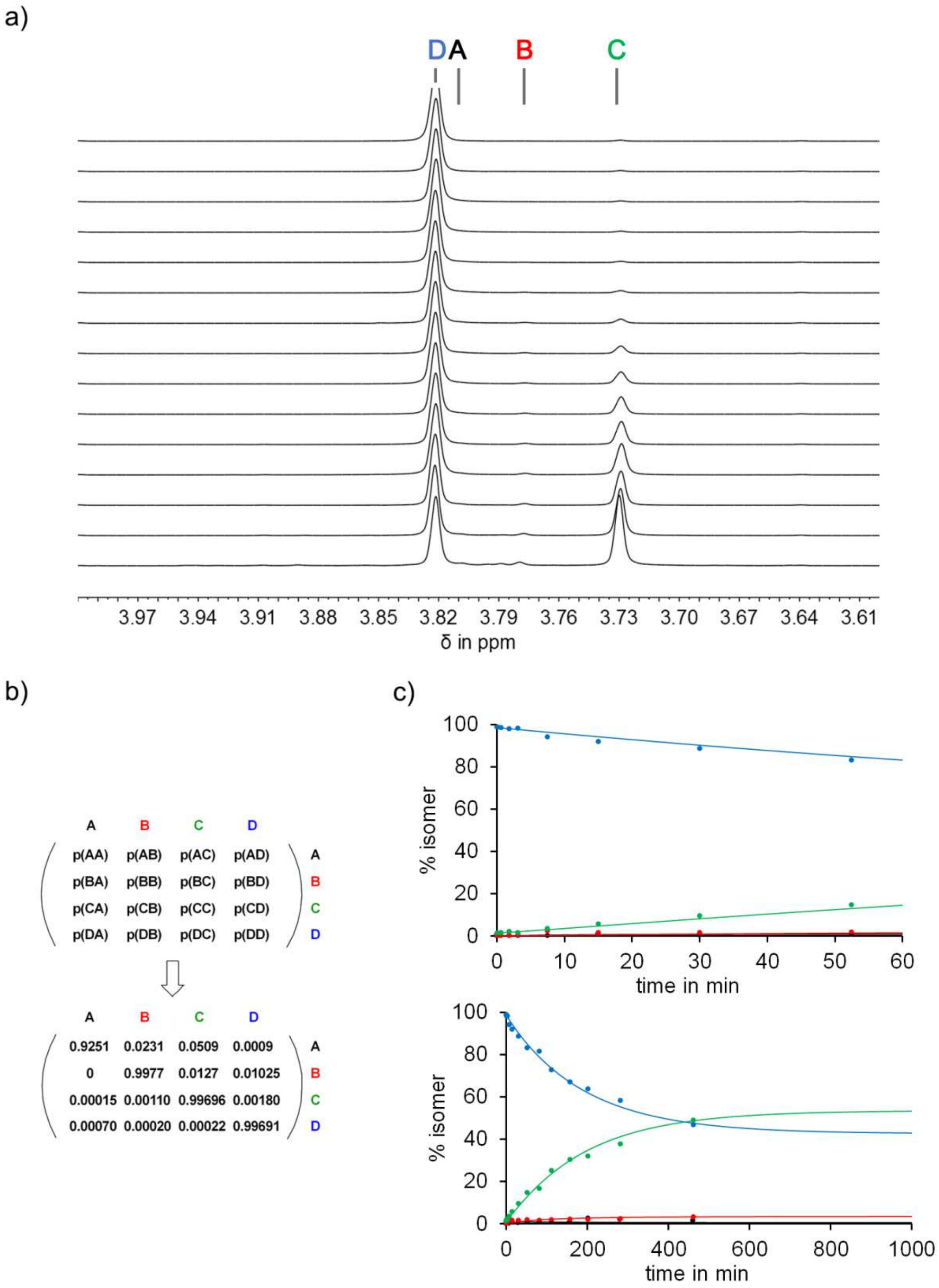

Supplementary Figure 27 | Markov matrix analysis of D in CD_2Cl_2. Markov matrix analysis of the photoreactions of **1** starting from isomer **D** in CD_2Cl_2 solution at 22 °C under 405 nm illumination. a) Section of the ^{1}H NMR spectra (400 MHz) recorded during irradiation of **D-1**. Each spectrum represents a set of data points in the kinetic plot. b) Markov matrix describing the phototransition probabilities p(**ij**) per minute for **1**. c) Kinetic plots of the experimentally determined changes in isomer composition during irradiation of **D-1** (dots) and fitted values derived from the Markov matrix (lines).

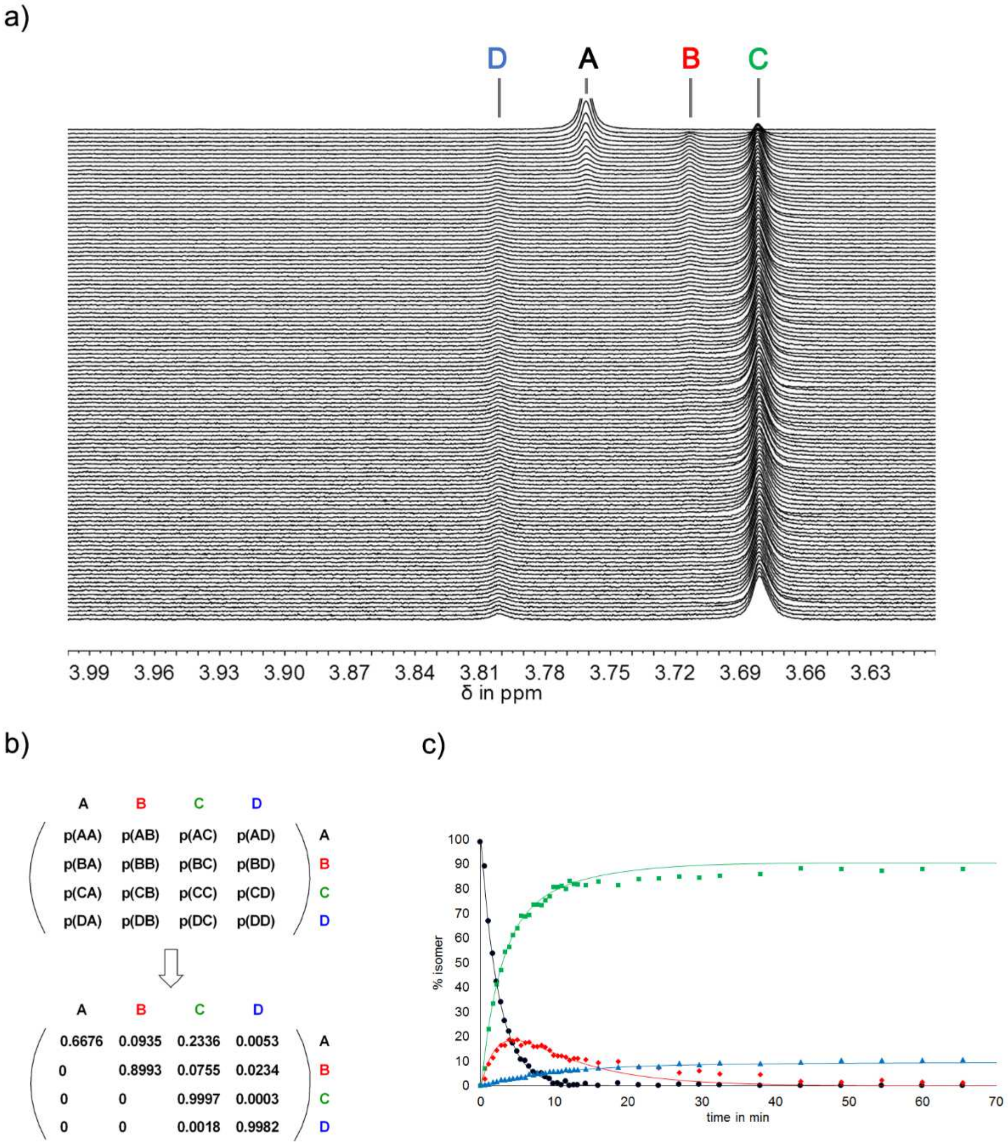

Supplementary Figure 28 | Markov matrix analysis of A in CD_2Cl_2 at -80 °C. Markov matrix analysis of the photoreactions of **1** starting from isomer **A** in CD_2Cl_2 solution at -80 °C under 405 nm illumination. a) Section of the ^{1}H NMR spectra (400 MHz) recorded during irradiation of **A-1**. Each spectrum represents a set of data points in the kinetic plot. b) Markov matrix describing the phototransition probabilities p(**ij**) per minute for **1**. c) Kinetic plots of the experimentally determined changes in isomer composition during irradiation of **A-1** (dots) and fitted values derived from the Markov matrix (lines).

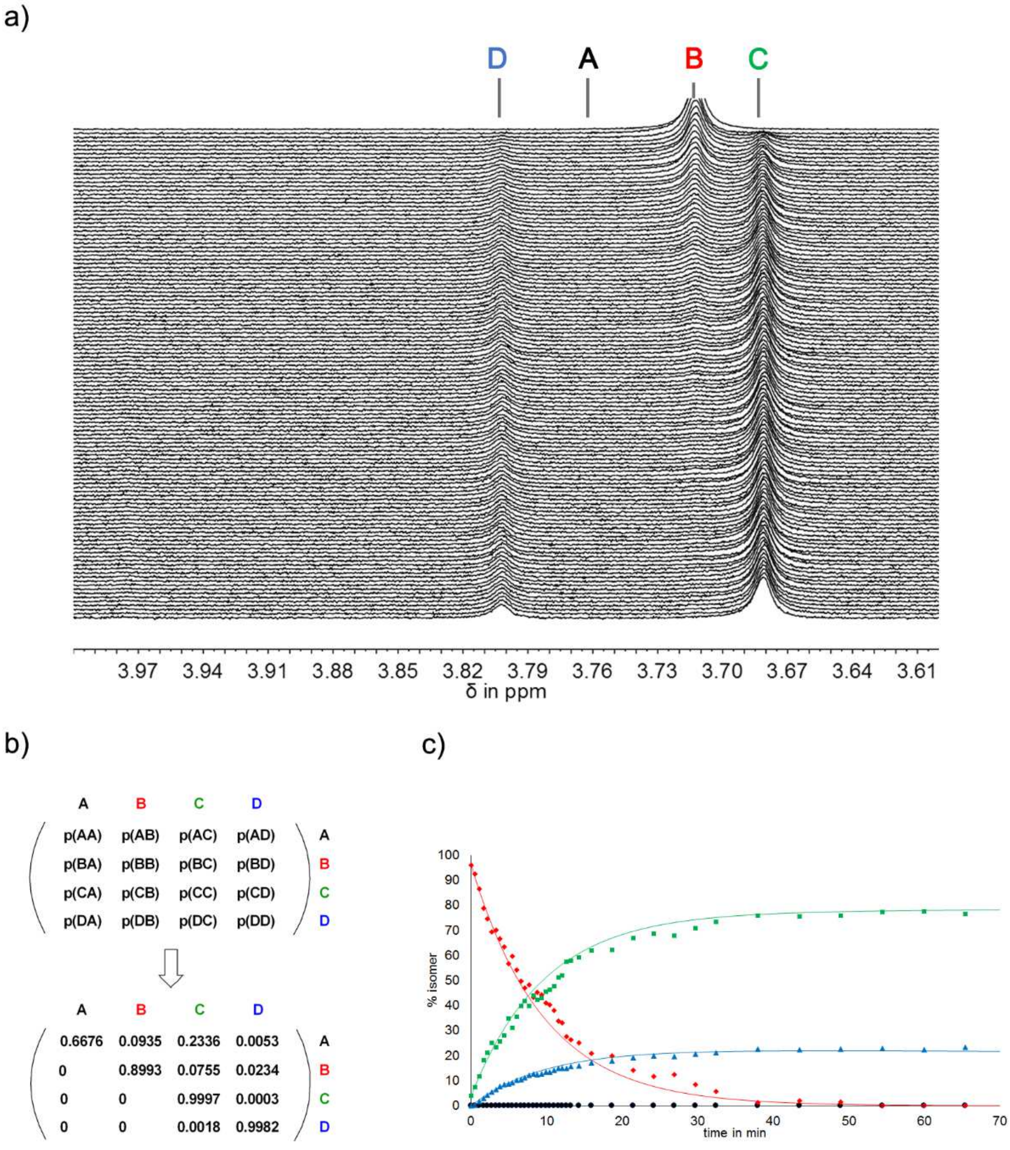

Supplementary Figure 29 | Markov matrix analysis of B in CD_2Cl_2 at -80 °C. Markov matrix analysis of the photoreactions of **1** starting from isomer **B** in CD_2Cl_2 solution at -80 °C under 405 nm illumination. a) Section of the ^{1}H NMR spectra (400 MHz) recorded during irradiation of **B-1**. Each spectrum represents a set of data points in the kinetic plot. b) Markov matrix describing the phototransition probabilities p(**ij**) per minute for **1**. c) Kinetic plots of the experimentally determined changes in isomer composition during irradiation of **B-1** (dots) and fitted values derived from the Markov matrix (lines).

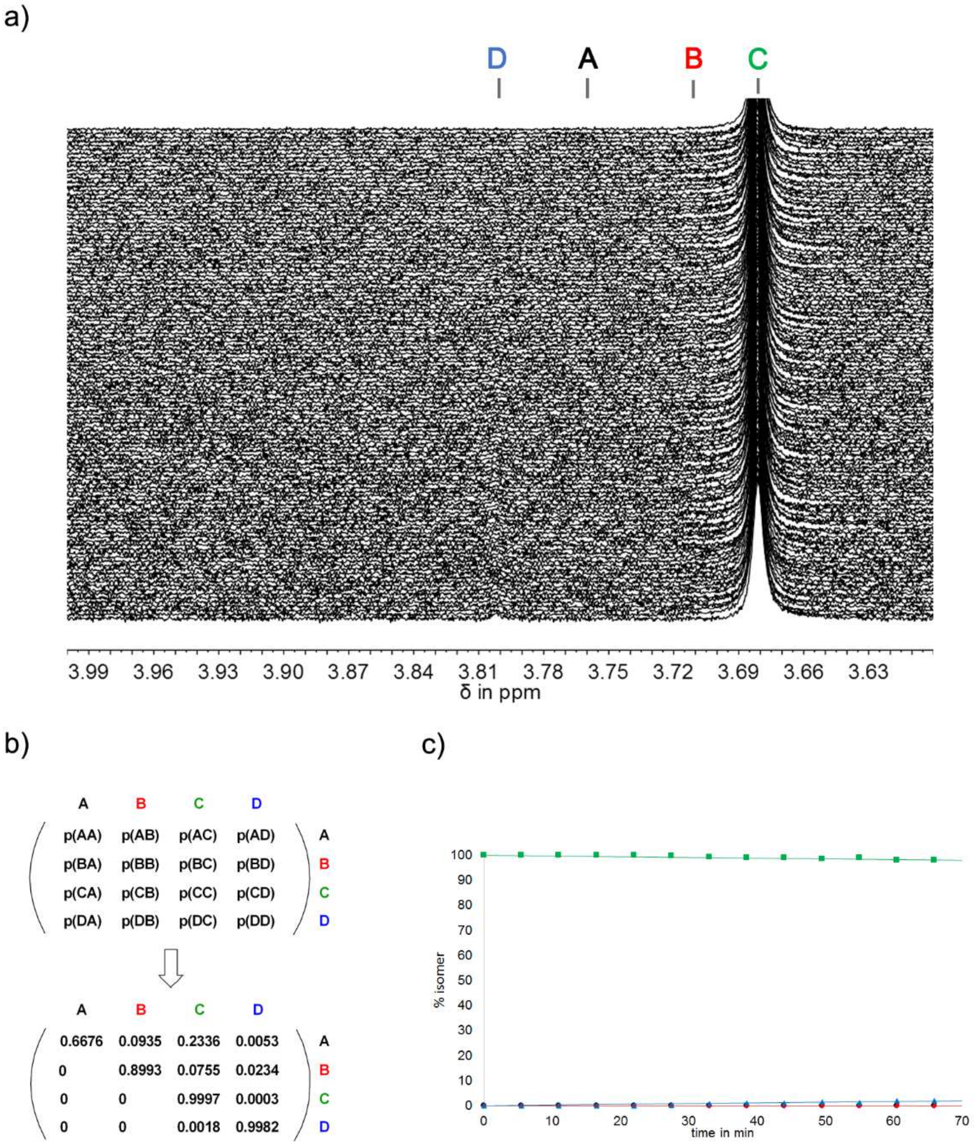

Supplementary Figure 30 | Markov matrix analysis of C in CD_2Cl_2 at -80 °C. Markov matrix analysis of the photoreactions of **1** starting from isomer **C** in CD_2Cl_2 solution at -80 °C under 405 nm illumination. a) Section of the ^{1}H NMR spectra (400 MHz) recorded during irradiation of **C**-**1**. Each spectrum represents a set of data points in the kinetic plot. b) Markov matrix describing the phototransition probabilities p(**ij**) per minute for **1**. c) Kinetic plots of the experimentally determined changes in isomer composition during irradiation of **C**-**1** (dots) and fitted values derived from the Markov matrix (lines).

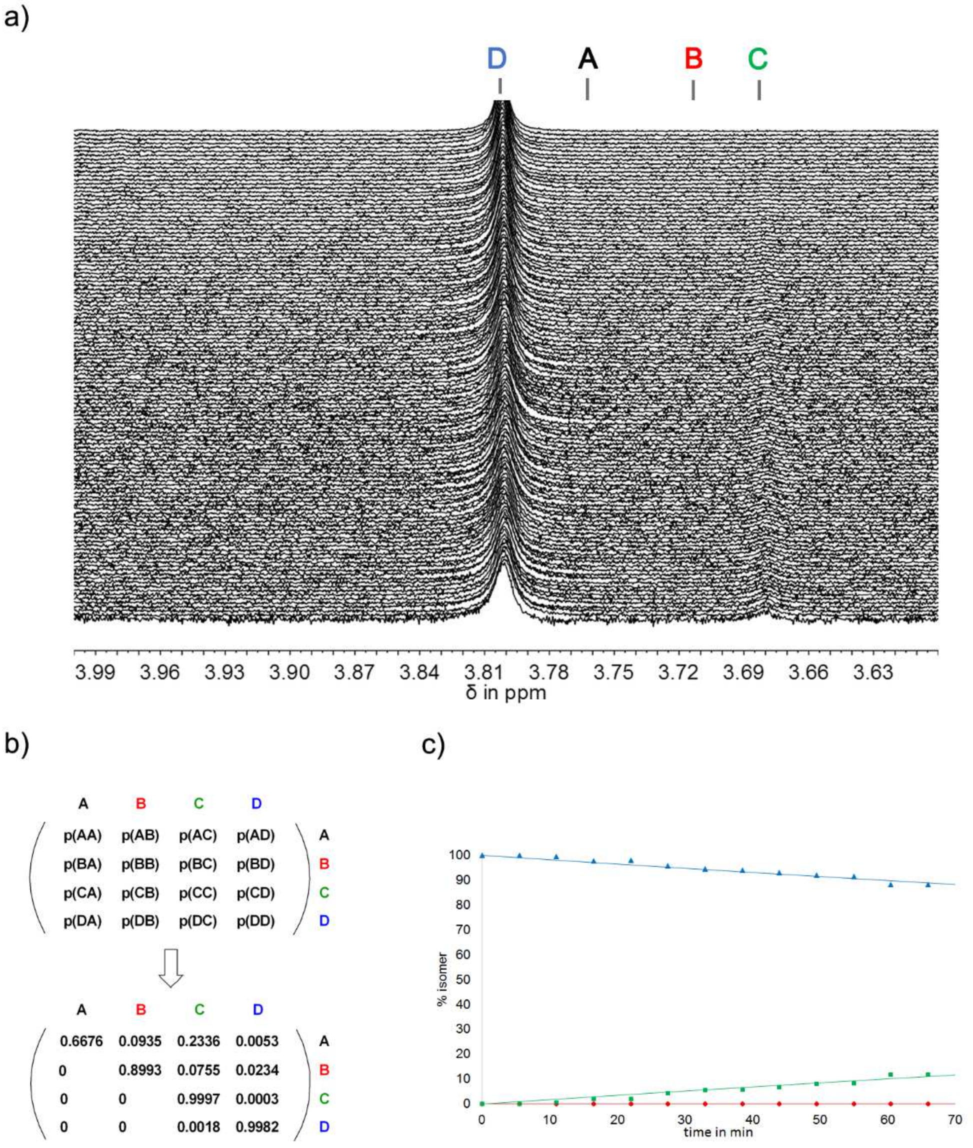

Supplementary Figure 31 | Markov matrix analysis of D in CD_2Cl_2 at -80 °C. Markov matrix analysis of the photoreactions of **1** starting from isomer **D** in CD_2Cl_2 solution at -80 °C under 405 nm illumination. a) Section of the ^{1}H NMR spectra (400 MHz) recorded during irradiation of **D-1**. Each spectrum represents a set of data points in the kinetic plot. b) Markov matrix describing the phototransition probabilities p(**ij**) per minute for **1**. c) Kinetic plots of the experimentally determined changes in isomer composition during irradiation of **D-1** (dots) and fitted values derived from the Markov matrix (lines).

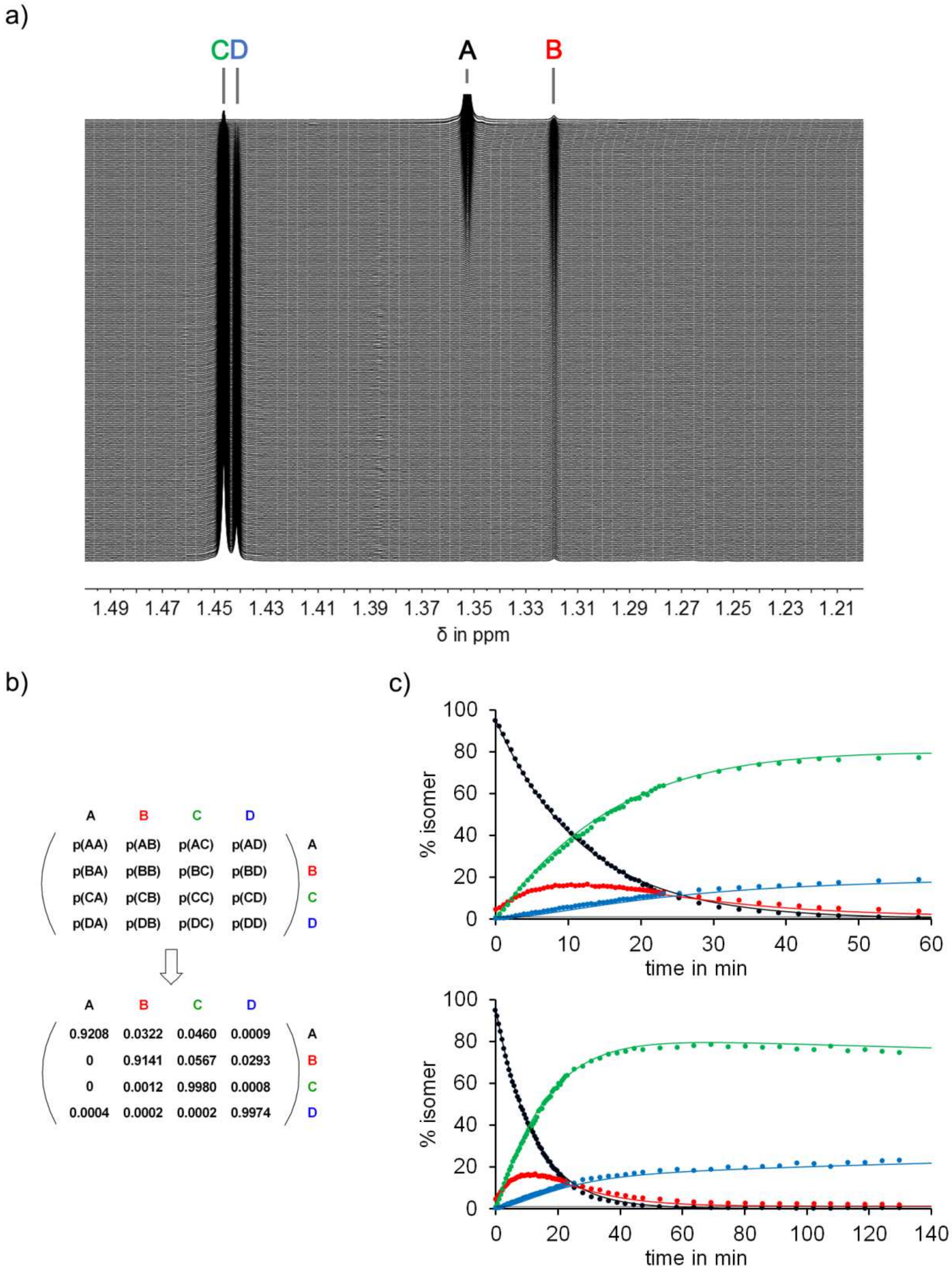

Supplementary Figure 32 | Markov matrix analysis of A in MeOH-d_4. Markov matrix analysis of the photoreactions of **1** starting from isomer **A** in MeOH-d_4 solution at 27 °C under 405 nm illumination. a) Section of the ^{1}H NMR spectra (400 MHz) recorded during irradiation of **A-1**. Spectra were recorded in 63 s intervals. b) Markov matrix describing the phototransition probabilities p(**ij**) per minute for **1**. c) Kinetic plots of the experimentally determined changes in isomer composition during irradiation of **A-1** (dots) and fitted values derived from the Markov matrix (lines).

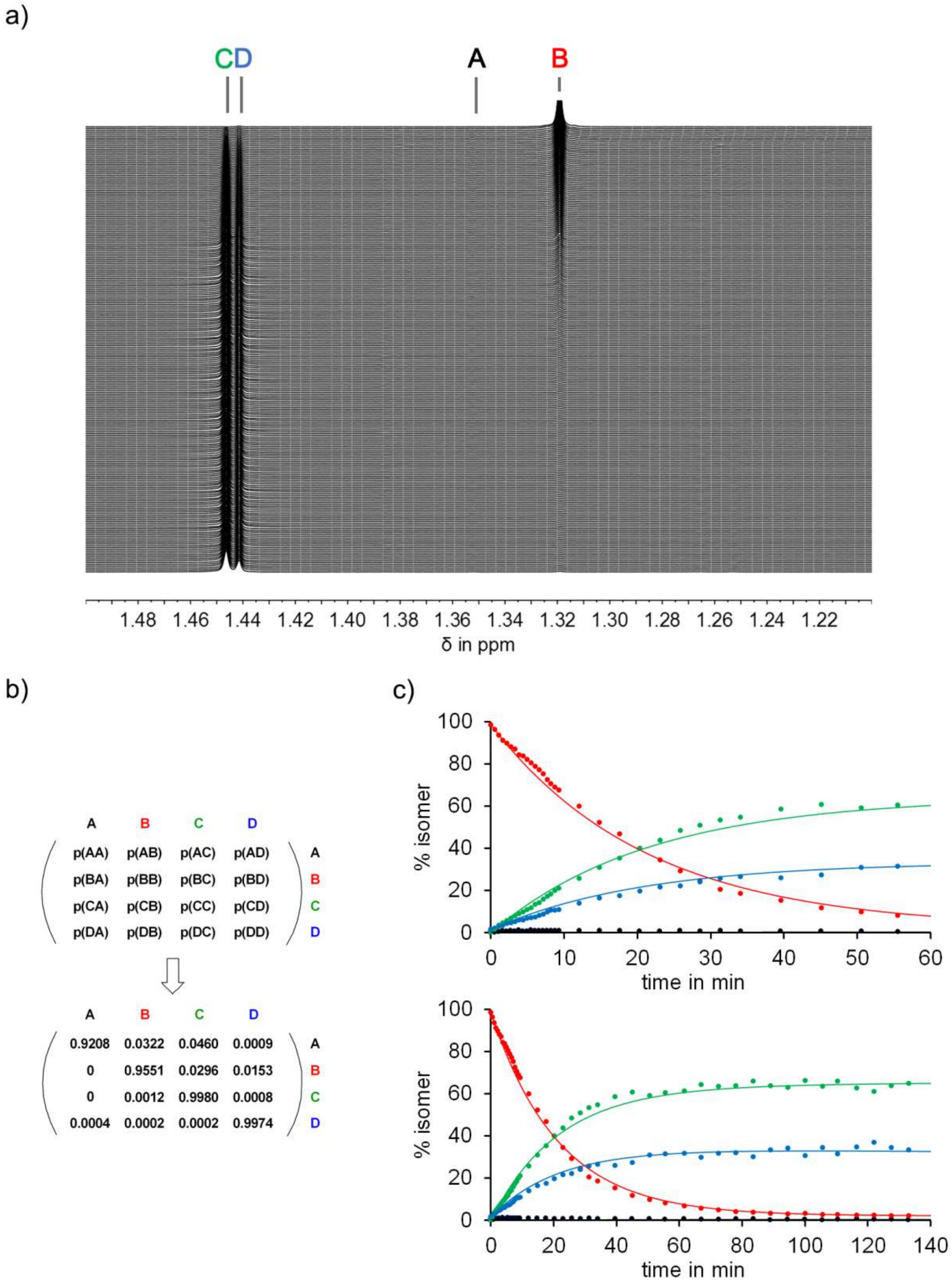

Supplementary Figure 33 | Markov matrix analysis of B in MeOH-*d*4. Markov matrix analysis of the photoreactions of **1** starting from isomer **B** in MeOH-d_4 solution at 27 °C under 405 nm illumination. a) Section of the ^{1}H NMR spectra (400 MHz) recorded during irradiation of **B-1**. Spectra were recorded in 63 s intervals. b) Markov matrix describing the phototransition probabilities p(**ij**) per minute for **1**. c) Kinetic plots of the experimentally determined changes in isomer composition during irradiation of **B-1** (dots) and fitted values derived from the Markov matrix (lines).

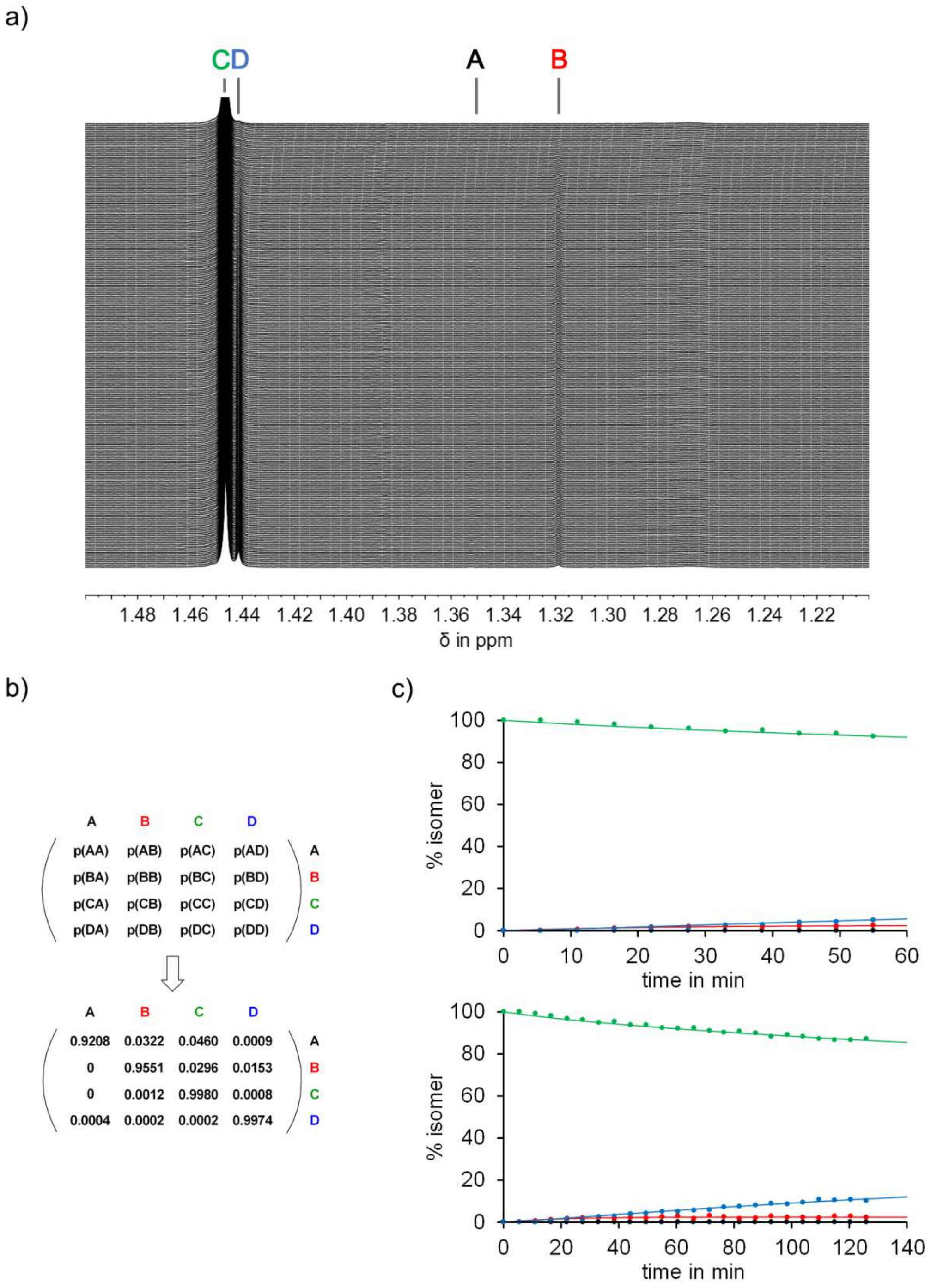

Supplementary Figure 34 | Markov matrix analysis of C in MeOH-d_4. Markov matrix analysis of the photoreactions of **1** starting from isomer **C** in MeOH-d_4 solution at 27 °C under 405 nm illumination. a) Section of the ^{1}H NMR spectra (400 MHz) recorded during irradiation of **C-1**. Spectra were recorded in 63 s intervals. b) Markov matrix describing the phototransition probabilities p(**ij**) per minute for **1**. c) Kinetic plots of the experimentally determined changes in isomer composition during irradiation of **C-1** (dots) and fitted values derived from the Markov matrix (lines).

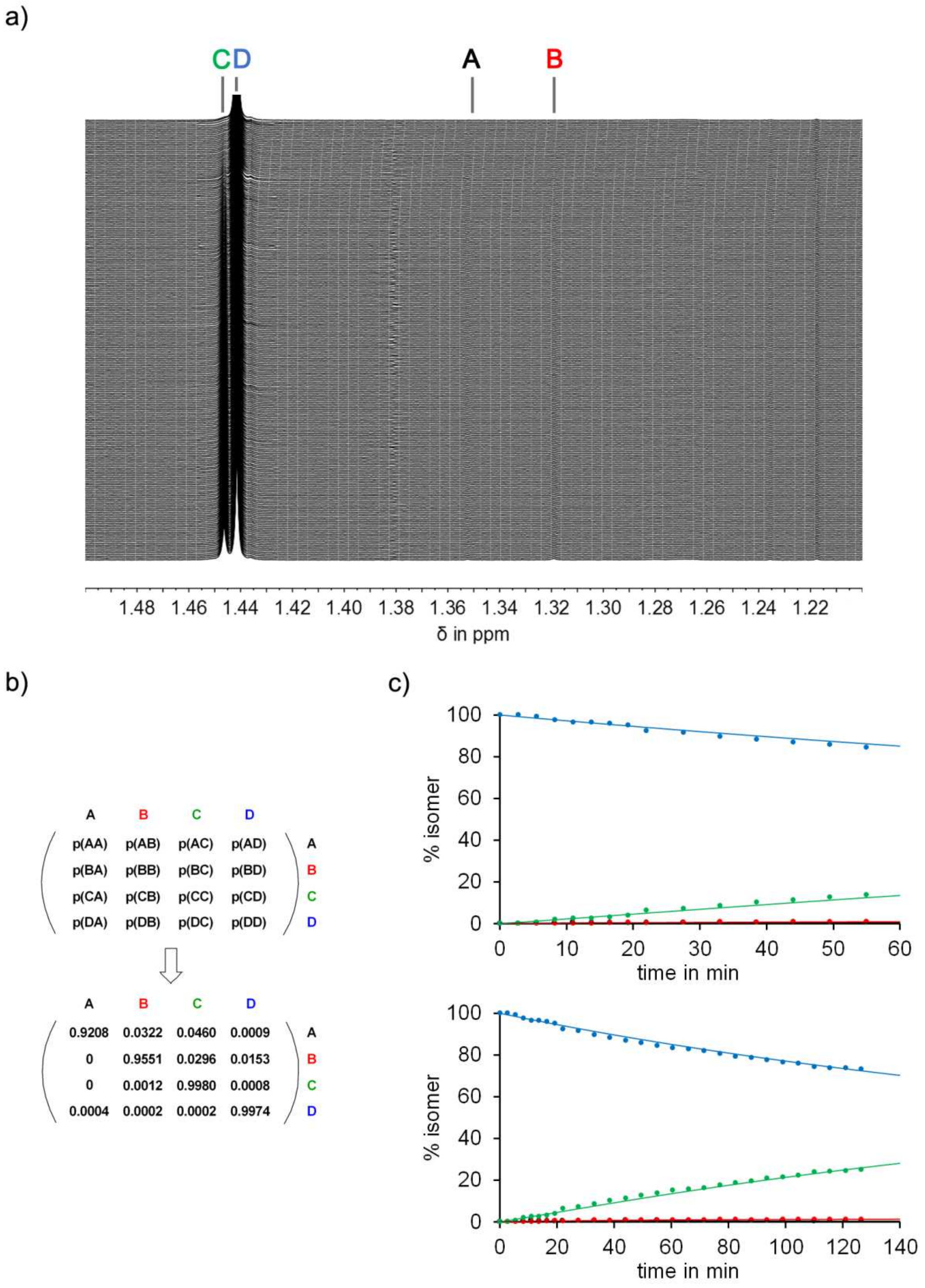

Supplementary Figure 35 | Markov matrix analysis of D in MeOH-d_4. Markov matrix analysis of the photoreactions of **1** starting from isomer **D** in MeOH-d_4 solution at 27 °C under 405 nm illumination. a) Section of the ^{1}H NMR spectra (400 MHz) recorded during irradiation of **D**-**1**. Spectra were recorded in 63 s intervals. b) Markov matrix describing the phototransition probabilities p(**ij**) per minute for **1**. c) Kinetic plots of the experimentally determined changes in isomer composition during irradiation of **D**-**1** (dots) and fitted values derived from the Markov matrix (lines).

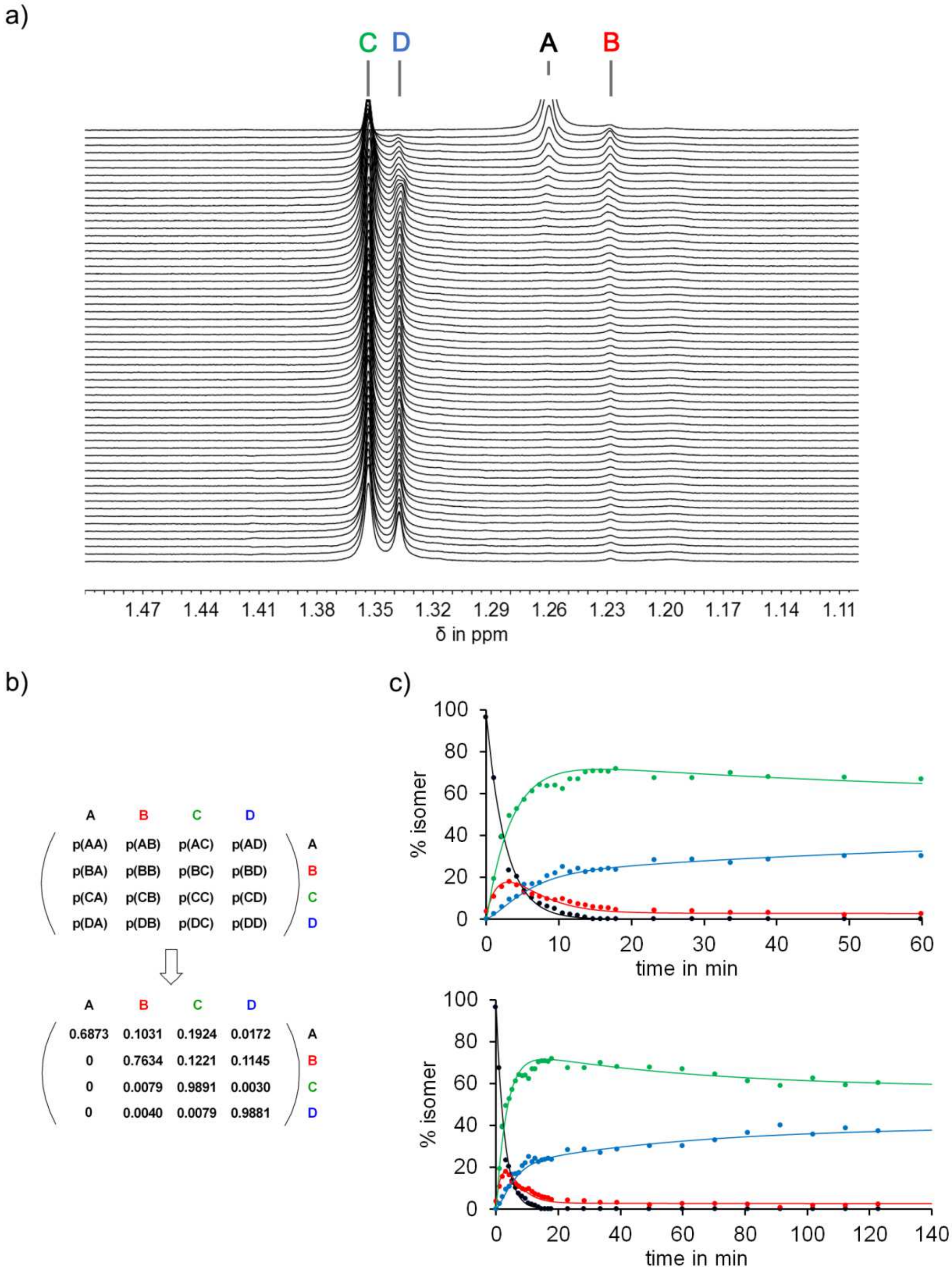

Supplementary Figure 36 | Markov matrix analysis of A in DMSO-d_6. Markov matrix analysis of the photoreactions of **1** starting from isomer **A** in DMSO-d_6 solution at 22 °C under 405 nm illumination. a) Section of the ^{1}H NMR spectra (400 MHz) recorded during irradiation of **A**-**1**. Spectra were recorded in 63 s intervals. b) Markov matrix describing the phototransition probabilities p(**ij**) per minute for **1**. c) Kinetic plots of the experimentally determined changes in isomer composition during irradiation of **A**-**1** (dots) and fitted values derived from the Markov matrix (lines).

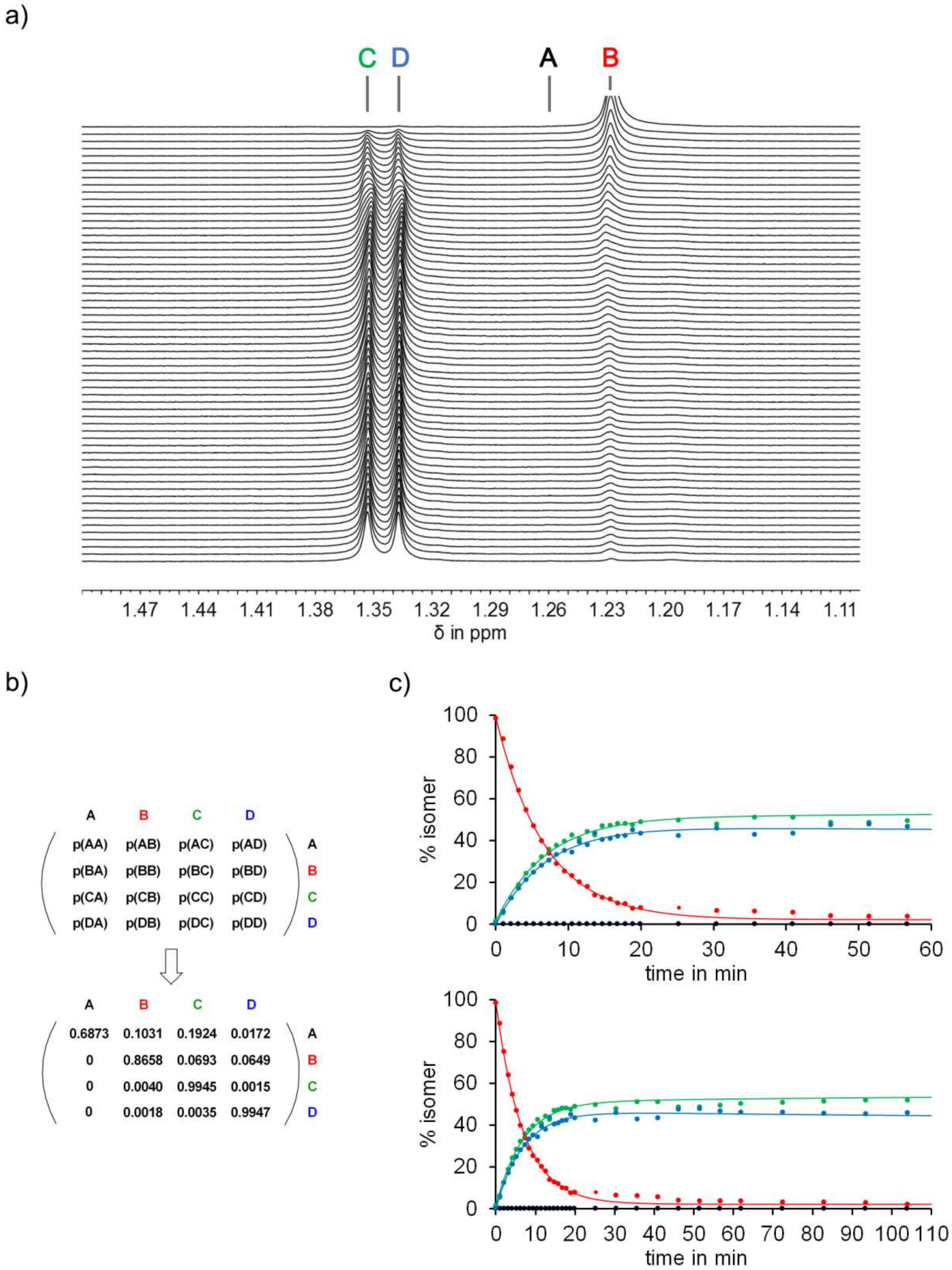

Supplementary Figure 37 | Markov matrix analysis of B in DMSO-d_6. Markov matrix analysis of the photoreactions of **1** starting from isomer **B** in DMSO-d_6 solution at 22 °C under 405 nm illumination. a) Section of the ^{1}H NMR spectra (400 MHz) recorded during irradiation of **B-1**. Spectra were recorded in 63 s intervals. b) Markov matrix describing the phototransition probabilities p(**ij**) per minute for **1**. c) Kinetic plots of the experimentally determined changes in isomer composition during irradiation of **B-1** (dots) and fitted values derived from the Markov matrix (lines).

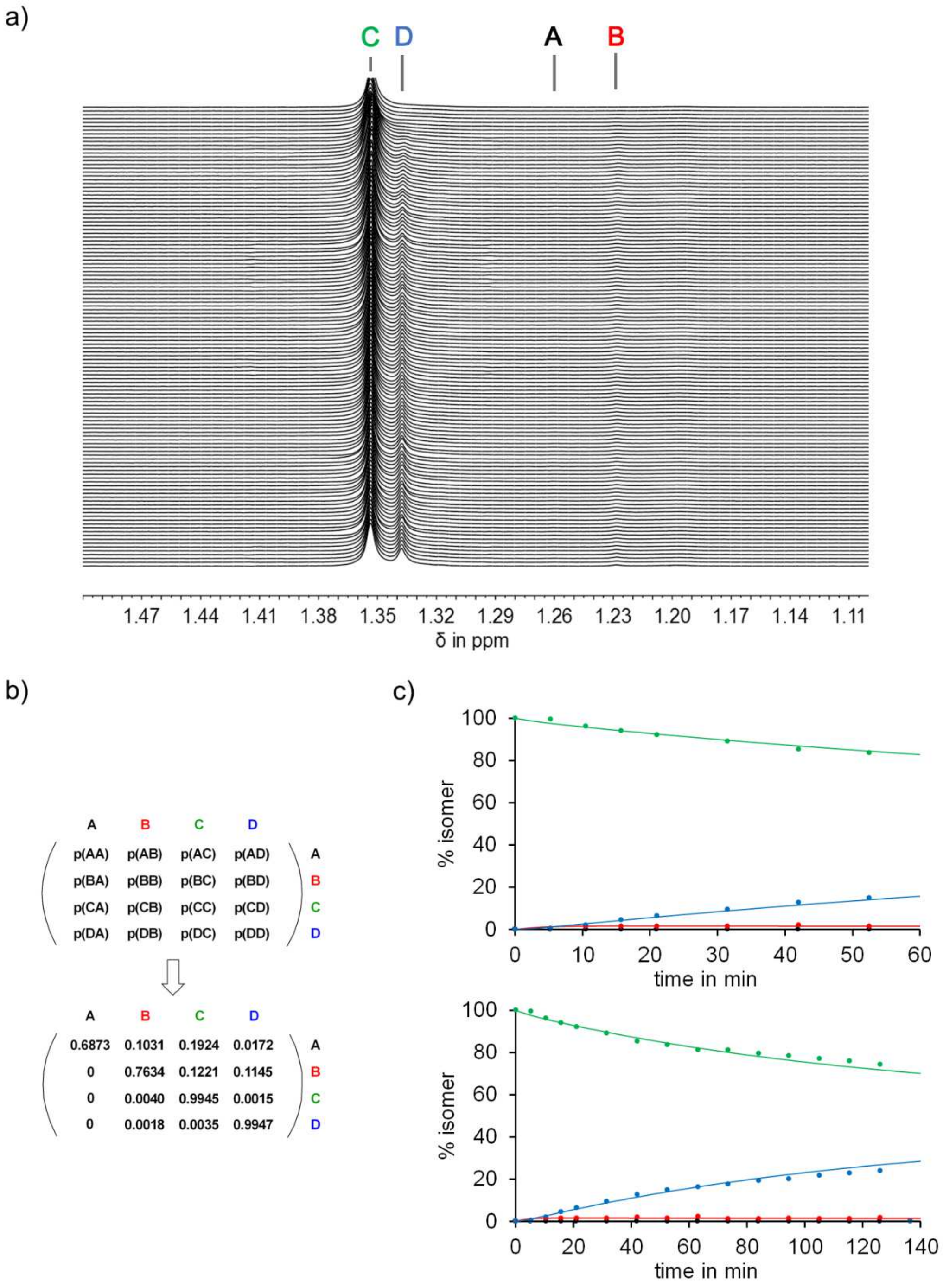

Supplementary Figure 38 | Markov matrix analysis of C in DMSO-d_6. Markov matrix analysis of the photoreactions of **1** starting from isomer **C** in DMSO-d_6 solution at 22 °C under 405 nm illumination. a) Section of the ^{1}H NMR spectra (400 MHz) recorded during irradiation of **C-1**. Spectra were recorded in 63 s intervals. b) Markov matrix describing the phototransition probabilities p(**ij**) per minute for **1**. c) Kinetic plots of the experimentally determined changes in isomer composition during irradiation of **C-1** (dots) and fitted values derived from the Markov matrix (lines).

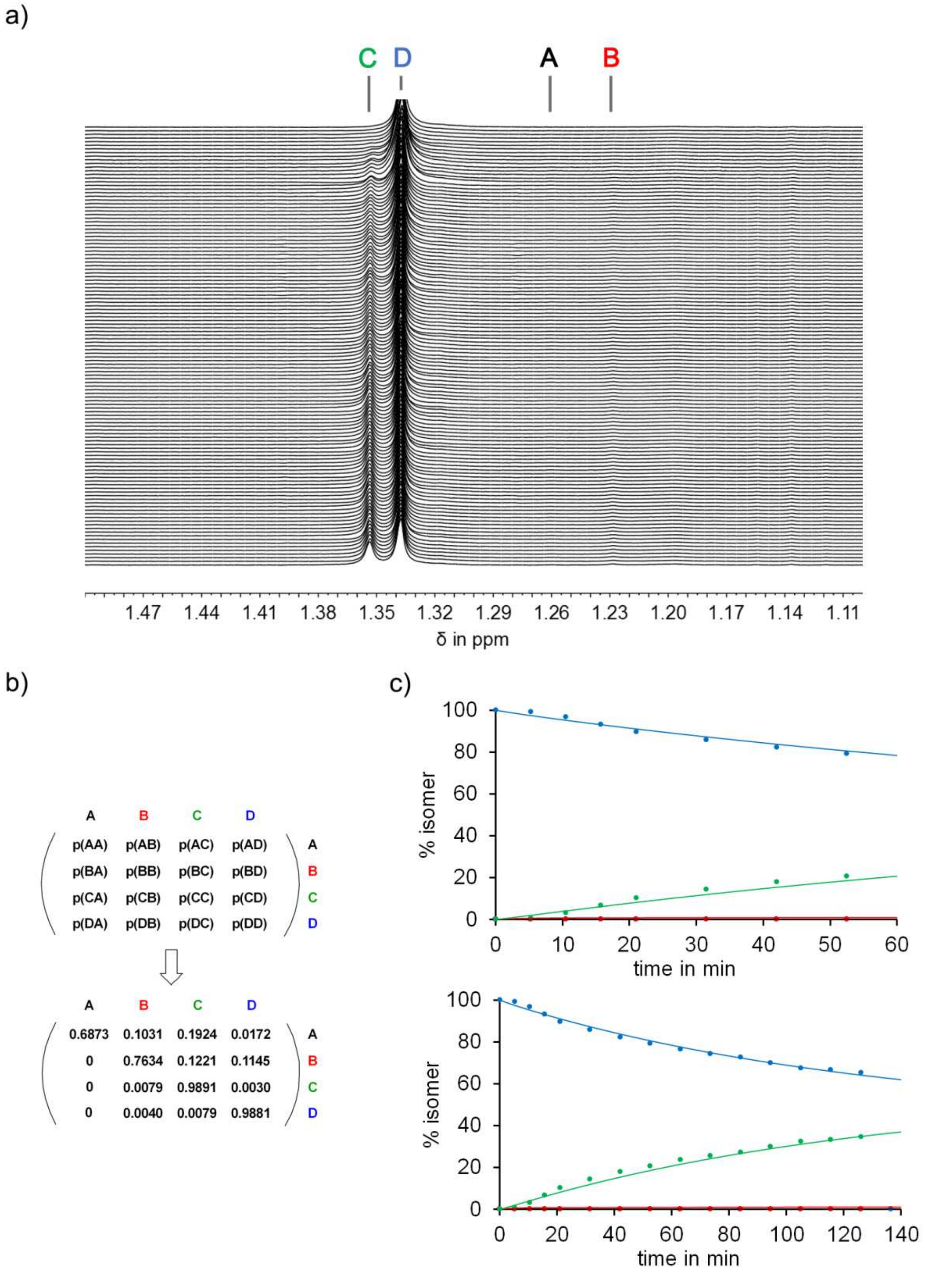

Supplementary Figure 39 | Markov matrix analysis of D in DMSO-d_6. Markov matrix analysis of the photoreactions of **1** starting from isomer **D** in DMSO-d_6 solution at 22 °C under 405 nm illumination. a) Section of the ^{1}H NMR spectra (400 MHz) recorded during irradiation of **D-1**. Spectra were recorded in 63 s intervals. b) Markov matrix describing the phototransition probabilities p(**ij**) per minute for **1**. c) Kinetic plots of the experimentally determined changes in isomer composition during irradiation of **D-1** (dots) and fitted values derived from the Markov matrix (lines).

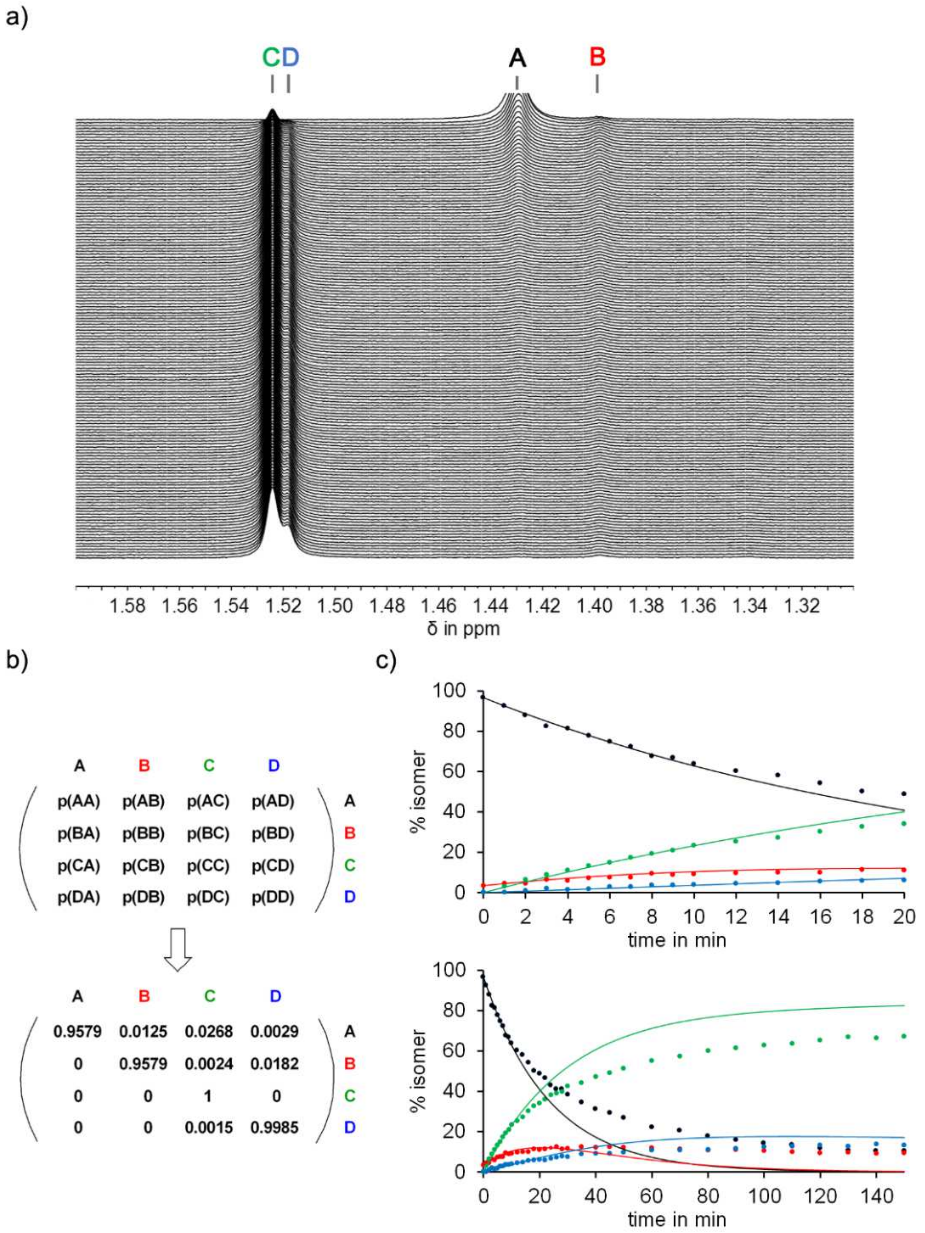

Supplementary Figure 40 | Markov matrix analysis of A in ethylene glycol. Markov matrix analysis of the photoreactions of **1** starting from isomer **A** in ethylene glycol solution at 22 °C under 405 nm illumination. The spectra were recorded with PRESAT solvent suppression and a benzene-d_6 capillary. a) Section of the ^{1}H NMR spectra (400 MHz) recorded during irradiation of **A-1**. Spectra were recorded in 60 s intervals. b) Markov matrix describing the phototransition probabilities p(**ij**) per minute for **1**. c) Kinetic plots of the experimentally determined changes in isomer composition during irradiation of **A-1** (dots) and fitted values derived from the Markov matrix (lines). As ethylene glycol is a very viscous liquid the diffusion inside the NMR-tube is very slow. Therefore unreacted **A**-1 from above the glass fiber or from the bottom of the NMR-tube diffuse slowly inside the measuring area and the kinetics slow down. This effect cannot be simulated by the Markov Matrix. Hence we used only the first minutes to fit the Markov Matrix as the diffusion should be negligible in this section.

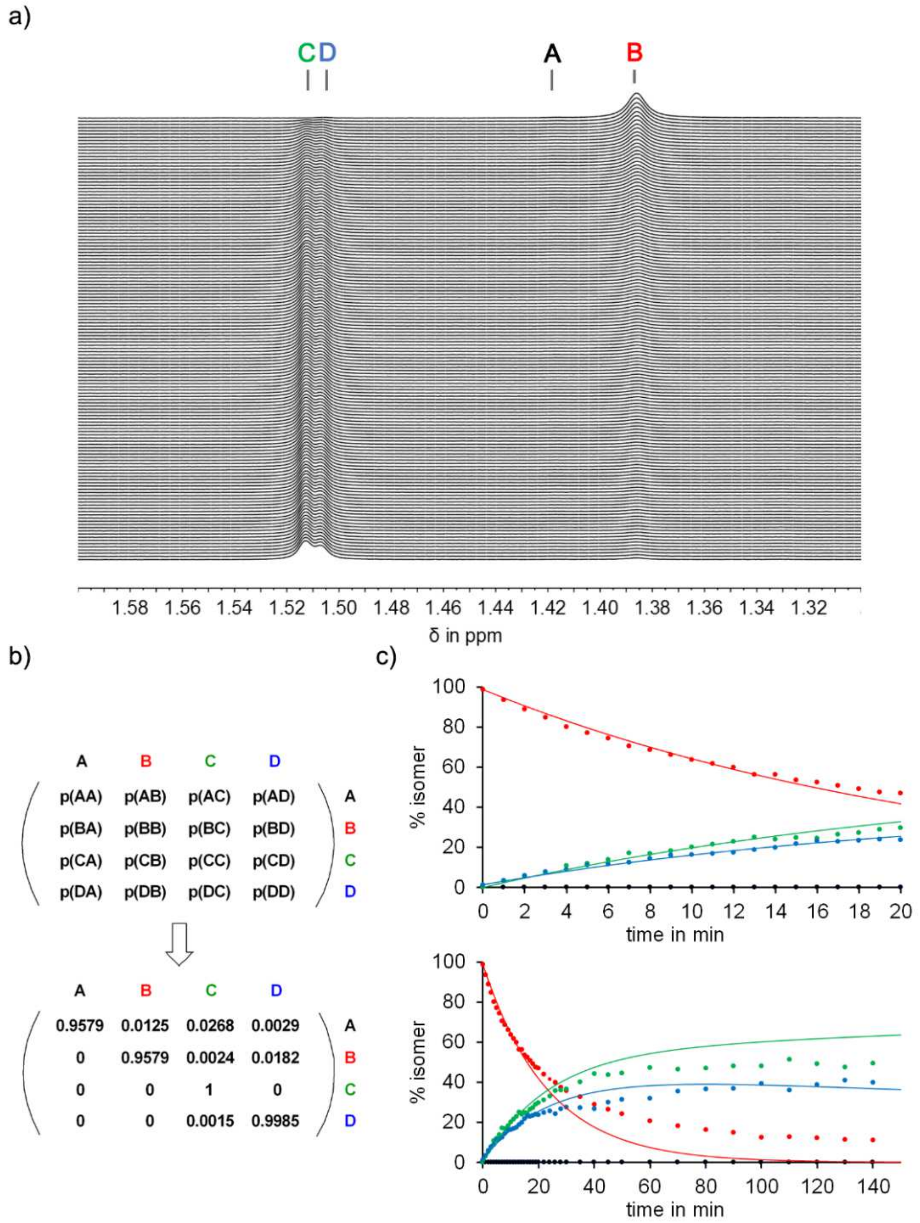

Supplementary Figure 41 | Markov matrix analysis of B in ethylene glycol. Markov matrix analysis of the photoreactions of **1** starting from isomer **B** in ethylene glycol solution at 22 °C under 405 nm illumination. The spectra were recorded with PRESAT solvent suppression and a benzene-d_6 capillary. a) Section of the ^{1}H NMR spectra (400 MHz) recorded during irradiation of **B**-**1**. Spectra were recorded in 60 s intervals. b) Markov matrix describing the phototransition probabilities p(**ij**) per minute for **1**. c) Kinetic plots of the experimentally determined changes in isomer composition during irradiation of **B**-**1** (dots) and fitted values derived from the Markov matrix (lines). As ethylene glycol is a very viscous liquid the diffusion inside the NMR-tube is very slow. Therefore unreacted **B**-1 from above the glass fiber or from the bottom of the NMR-tube diffuse slowly inside the measuring area and the kinetics slow down. This effect cannot be simulated by the Markov Matrix. Hence we used only the first minutes to fit the Markov Matrix as the diffusion should be negligible in this section.

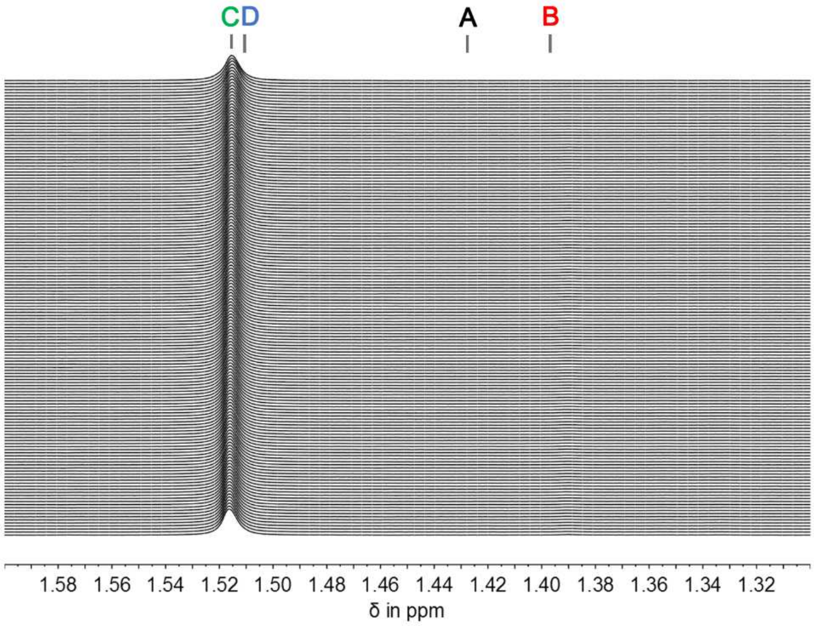

Supplementary Figure 42 | Markov matrix analysis of C in ethylene glycol. Markov matrix analysis of the photoreactions of **1** starting from isomer **C** in ethylene glycol solution at 22 °C under 405 nm illumination. The spectra were recorded with PRESAT solvent suppression and a benzene-d_6 capillary. a) Section of the ^{1}H NMR spectra (400 MHz) recorded during irradiation of **C**-**1**. Spectra were recorded in 60 s intervals. No changes in isomer concentration could be measured although the experiment was conducted twice. Probably the weak signal of upcoming isomer **D** could not be detected because it's overlapping with the signal of **C.** Nevertheless this experiment shows that the photo isomerization of **C** is very slow in comparison with those of **A** and **B** and therefore does not affect the kinetics of **A** and **B**.

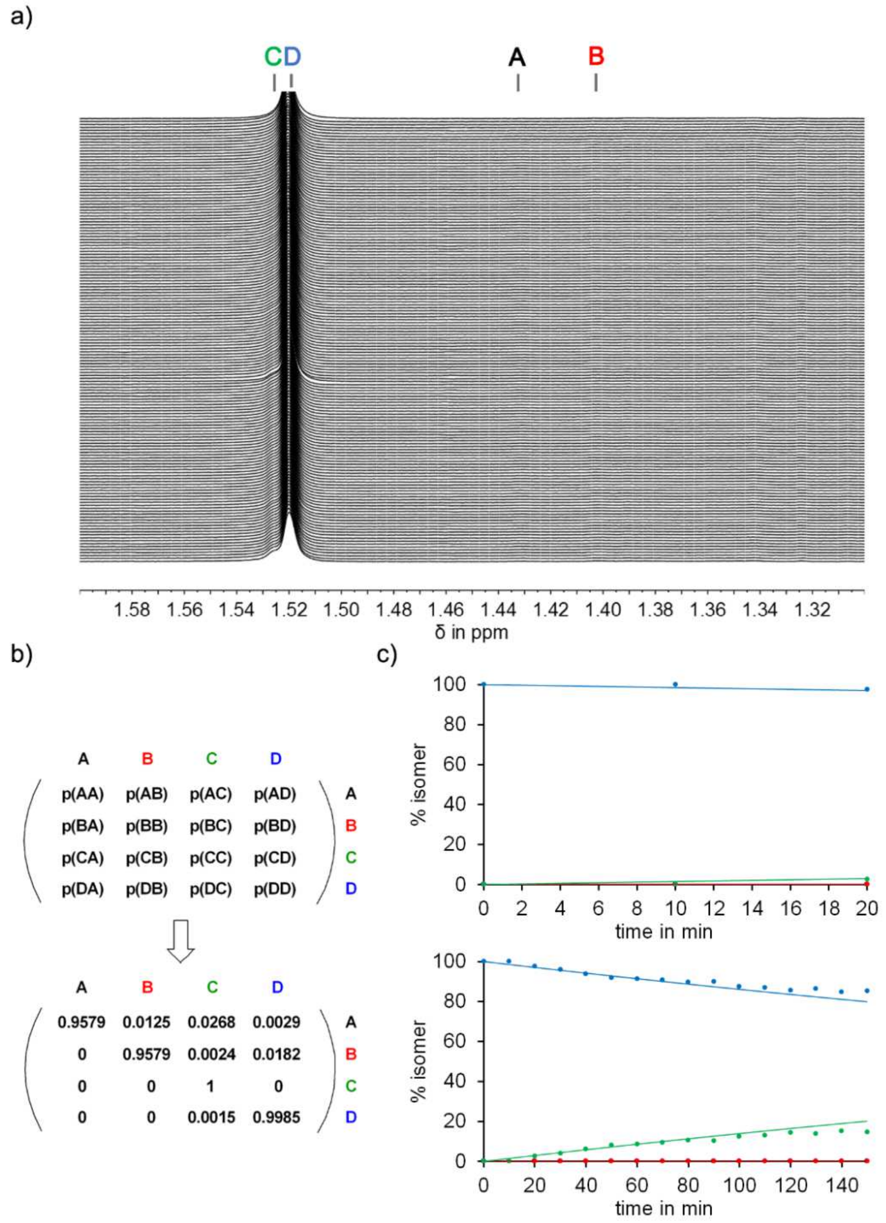

Supplementary Figure 43 | Markov matrix analysis of D in ethylene glycol. Markov matrix analysis of the photoreactions of **1** starting from isomer **D** in ethylene glycol solution at 22 °C under 405 nm illumination. The spectra were recorded with PRESAT solvent suppression and a benzene-d_6 capillary. a) Section of the ^{1}H NMR spectra (400 MHz) recorded during irradiation of **D-1**. Spectra were recorded in 60 s intervals. b) Markov matrix describing the phototransition probabilities p(**ij**) per minute for **1**. c) Kinetic plots of the experimentally determined changes in isomer composition during irradiation of **D-1** (dots) and fitted values derived from the Markov matrix (lines). As ethylene glycol is a very viscous liquid the diffusion inside the NMR-tube is very slow. Therefore unreacted **D**-1 from above the glass fiber or from the bottom of the NMR-tube diffuse slowly inside the measuring area and the kinetics slow down. This effect cannot be simulated by the Markov Matrix. Hence we used only the first minutes to fit the Markov Matrix as the diffusion should be negligible in this section.

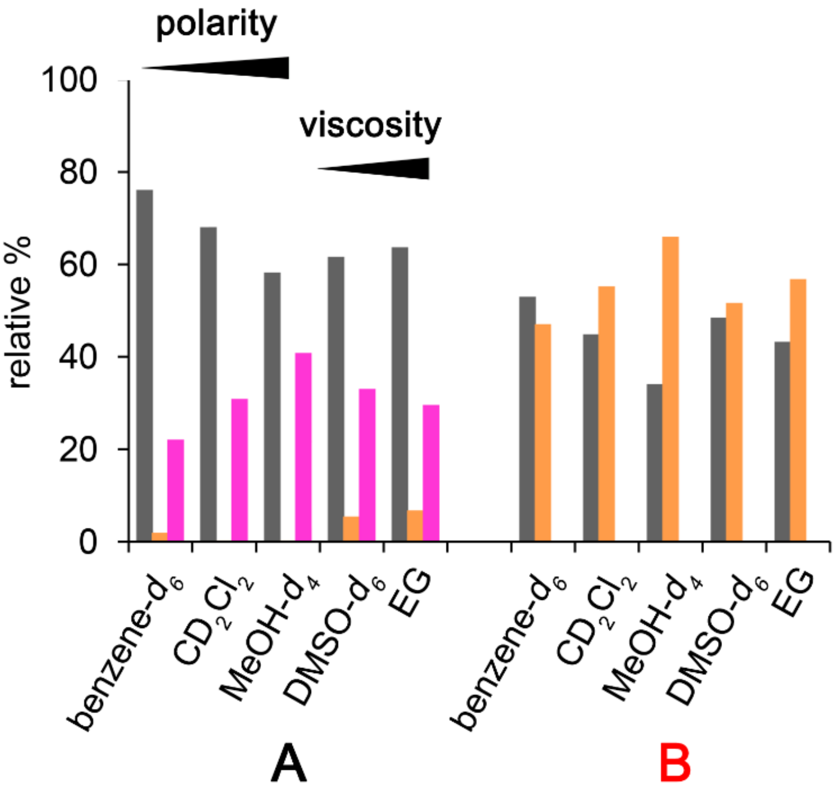

Supplementary Figure 44 | Relative quantum yields for 1 in different solvents. Markov matrix analysis of the photoreactions of **1** in different solvents at 27 °C under 405 nm illumination. The relative efficiency of different processes is given when solvent polarity and viscosity are varied independently from each other. The different photoprocesses are color coded: single-bond rotation (SBR) in purple, double-bond isomerization (DBI) in grey, and hula twist (HT) in orange.

Comparison of the photoconversion of A and B at different temperatures

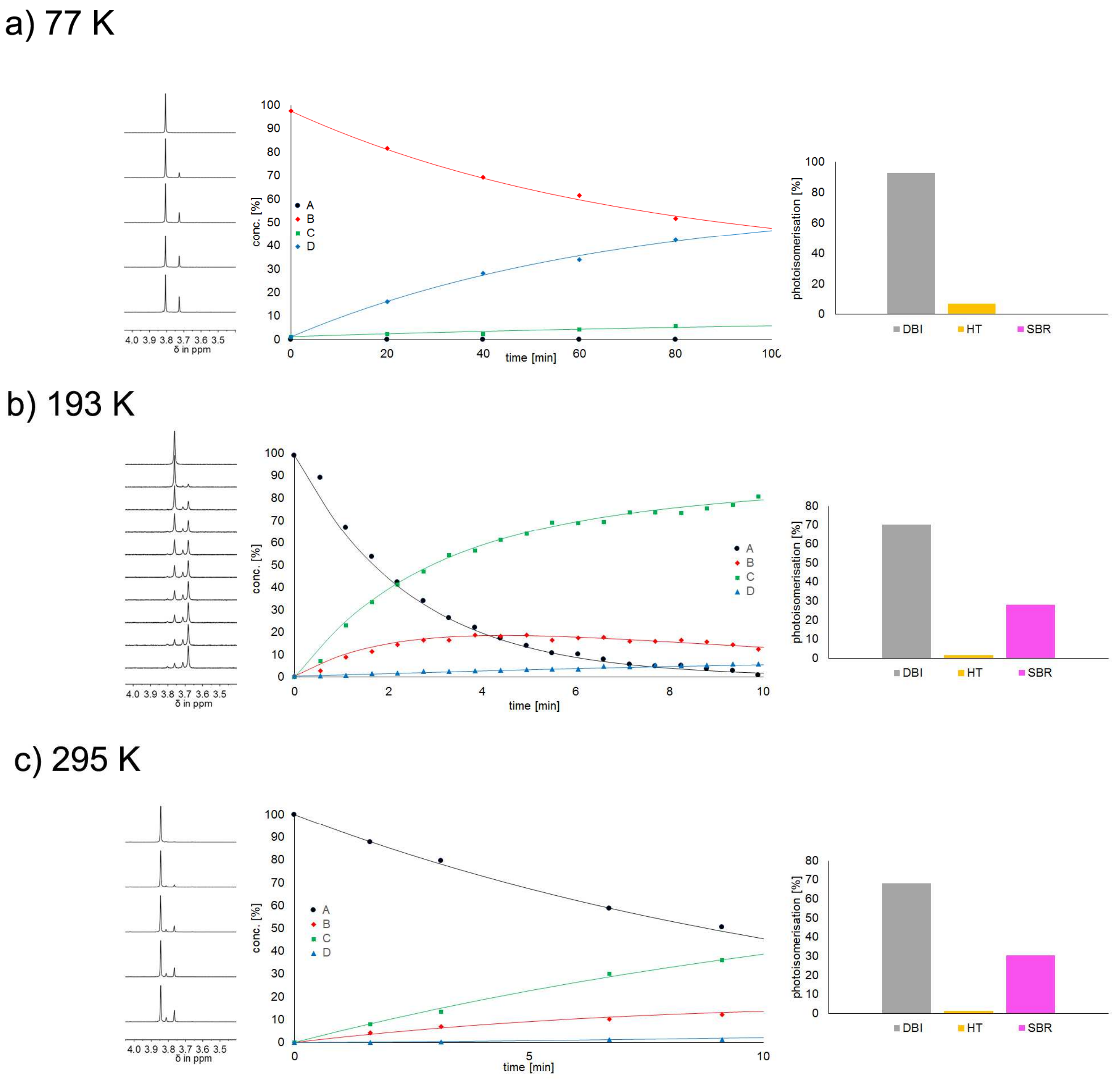

Supplementary Figure 45 | Photoreactions of A in CD_2Cl_2 at different temperatures. ^{1}H NMR spectra, Markov matrix analysis, and comparison of the different photoreactions (double-bond isomerization (DBI), hula twist (HT), single-bond rotation (SBR)) at a) 77 K (ice), b) 193 K (liquid), and c) 295 K (liquid) under 405 nm illumination. The rates of the photoreactions at different temperatures cannot be compared with each other as the illumination conditions are not comparable at different temperatures. Kinetic plots of the experimentally determined changes in isomer composition during irradiation of **A-1** (dots) are fitted by a simplified Markov matrix (lines) using phototransition probabilities of **C-1** = 0 and **D-1** = 0 since these transitions are much slower and therefore negligible in the experiments.

a) 77 K

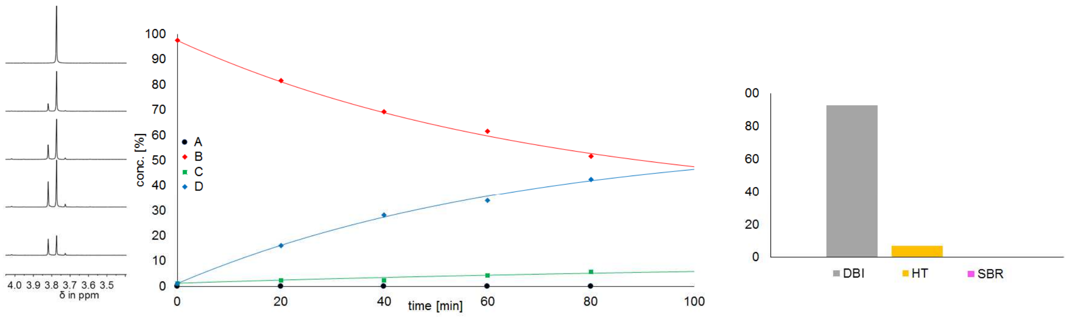

b) 193 K

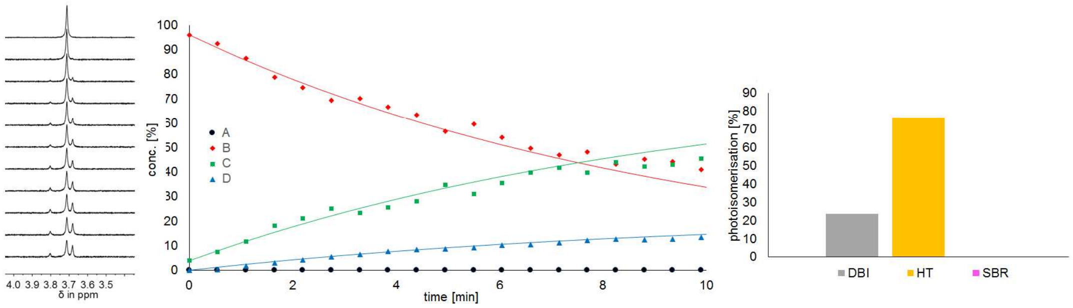

c) 295 K

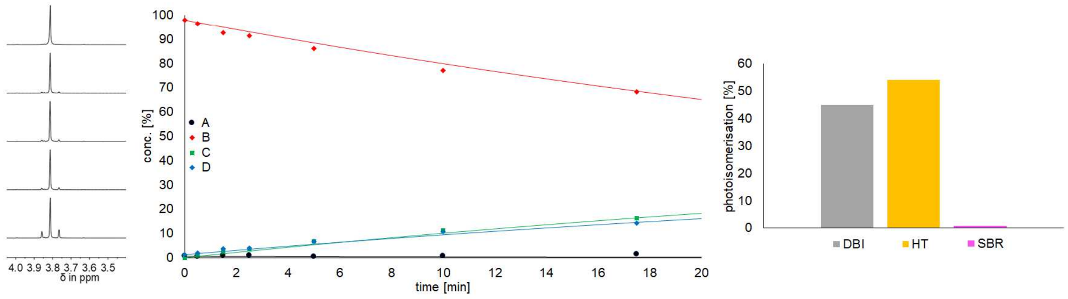

Supplementary Figure 46 | Photoreactions of B in CD_2Cl_2 at different temperatures. ^{1}H NMR spectra, Markov matrix analysis, and comparison of the different photoreactions (double-bond isomerization (DBI), hula twist (HT), single-bond rotation (SBR)) at a) 77 K (ice), b) 193 K (liquid), and c) 295 K (liquid) under 405 nm illumination. The rates of the photoreactions at different temperatures cannot be compared with each other as the illumination conditions are not comparable at different temperatures. Kinetic plots of the experimentally determined changes in isomer composition during irradiation of **B-1** (dots) are fitted by a simplified Markov matrix (lines) using phototransition probabilities of **C-1** = 0 and **D-1** = 0 since these transitions are much slower and therefore negligible in the experiments.

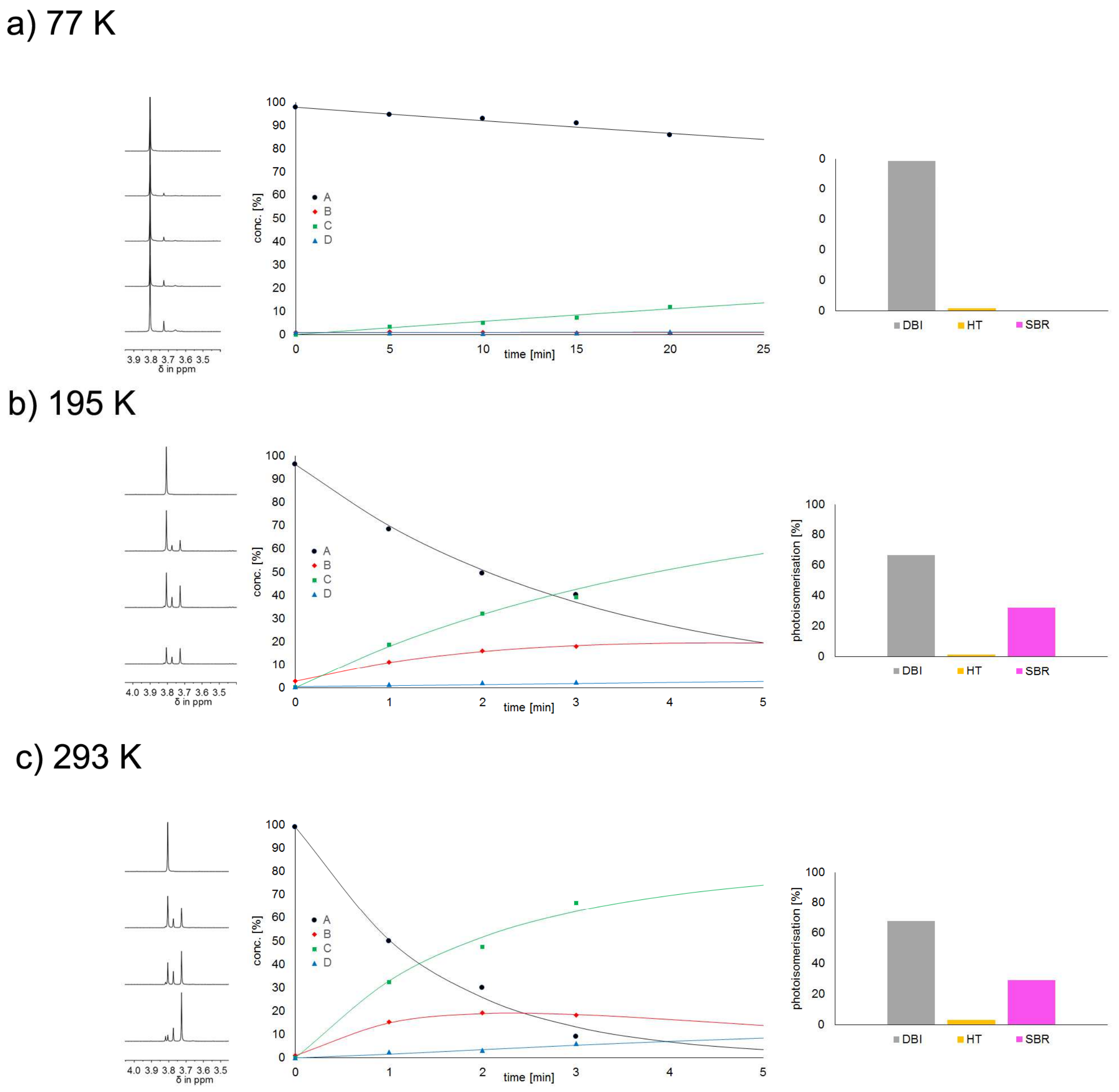

Supplementary Figure 47 | Photoreactions of A in EPA at different temperatures. ^{1}H NMR spectra in CD_2Cl_2, Markov matrix analysis, and comparison of the different photoreactions (double-bond isomerization (DBI), hula twist (HT), single-bond rotation (SBR)) at a) 77 K (ice), b) 193 K (liquid), and c) 295 K (liquid) under 405 nm illumination. The rates of the photoreactions at different temperatures cannot be compared with each other as the illumination conditions are not comparable at different temperatures. Kinetic plots of the experimentally determined changes in isomer composition during irradiation of **A-1** (dots) are fitted by a simplified Markov matrix (lines) using phototransition probabilities of **C-1** = 0 and **D-1** = 0 since these transitions are much slower and therefore negligible in the experiments.

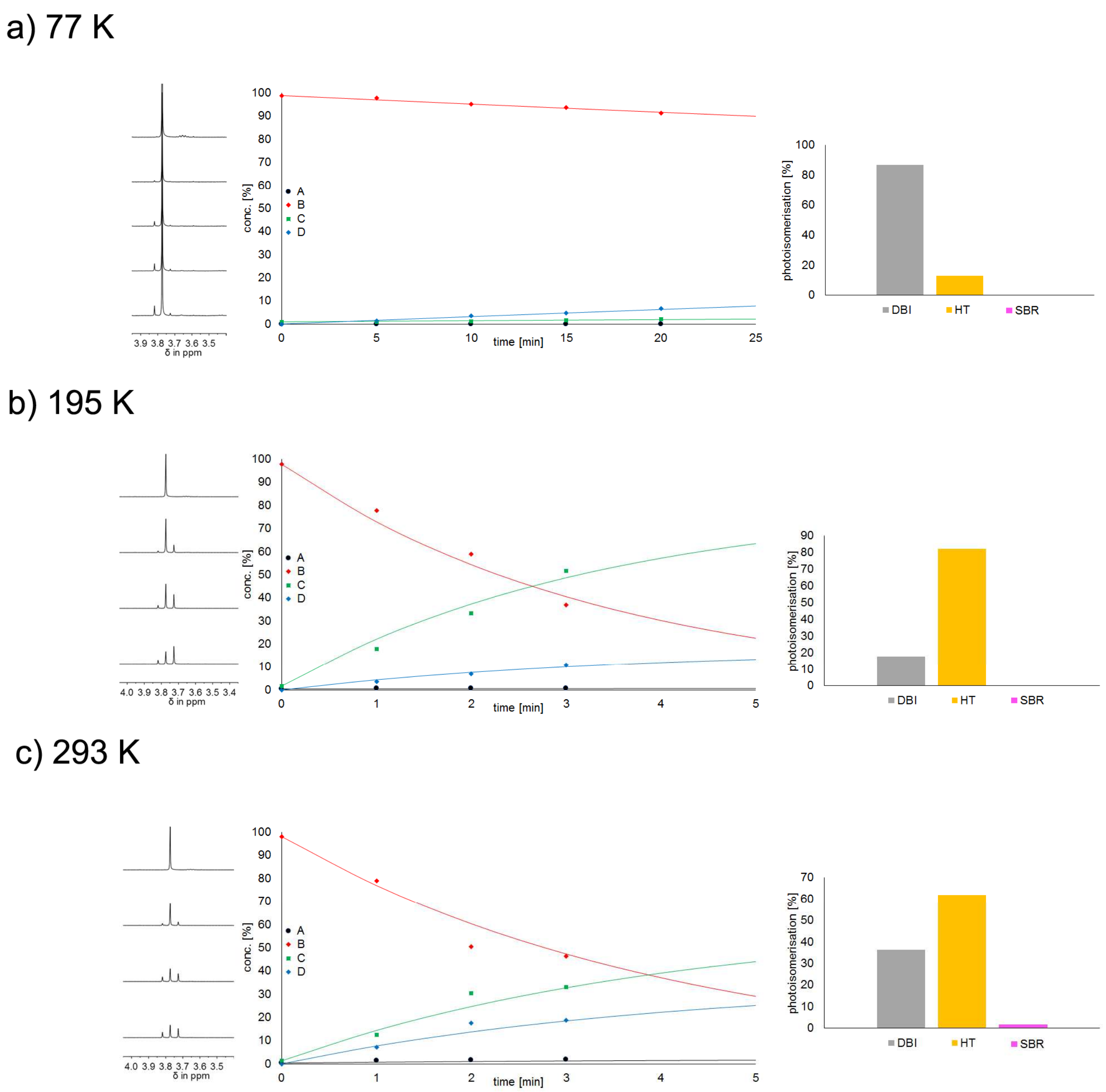

Supplementary Figure 48 | Photoreactions of B in EPA at different temperatures. Photoreactions of **B-1** in EPA at different temperatures. ^{1}H NMR spectra in CD_2Cl_2, Markov matrix analysis, and comparison of the different photoreactions (double-bond isomerization (DBI), hula twist (HT), single-bond rotation (SBR)) at a) 77 K (ice), b) 193 K (liquid), and c) 295 K (liquid) under 405 nm illumination. The rates of the photoreactions at different temperatures cannot be compared with each other as the illumination conditions are not comparable at different temperatures. Kinetic plots of the experimentally determined changes in isomer composition during irradiation of **B-1** (dots) are fitted by a simplified Markov matrix (lines) using phototransition probabilities of **C-1** = 0 and **D-1** = 0 since these transitions are much slower and therefore negligible in the experiments.

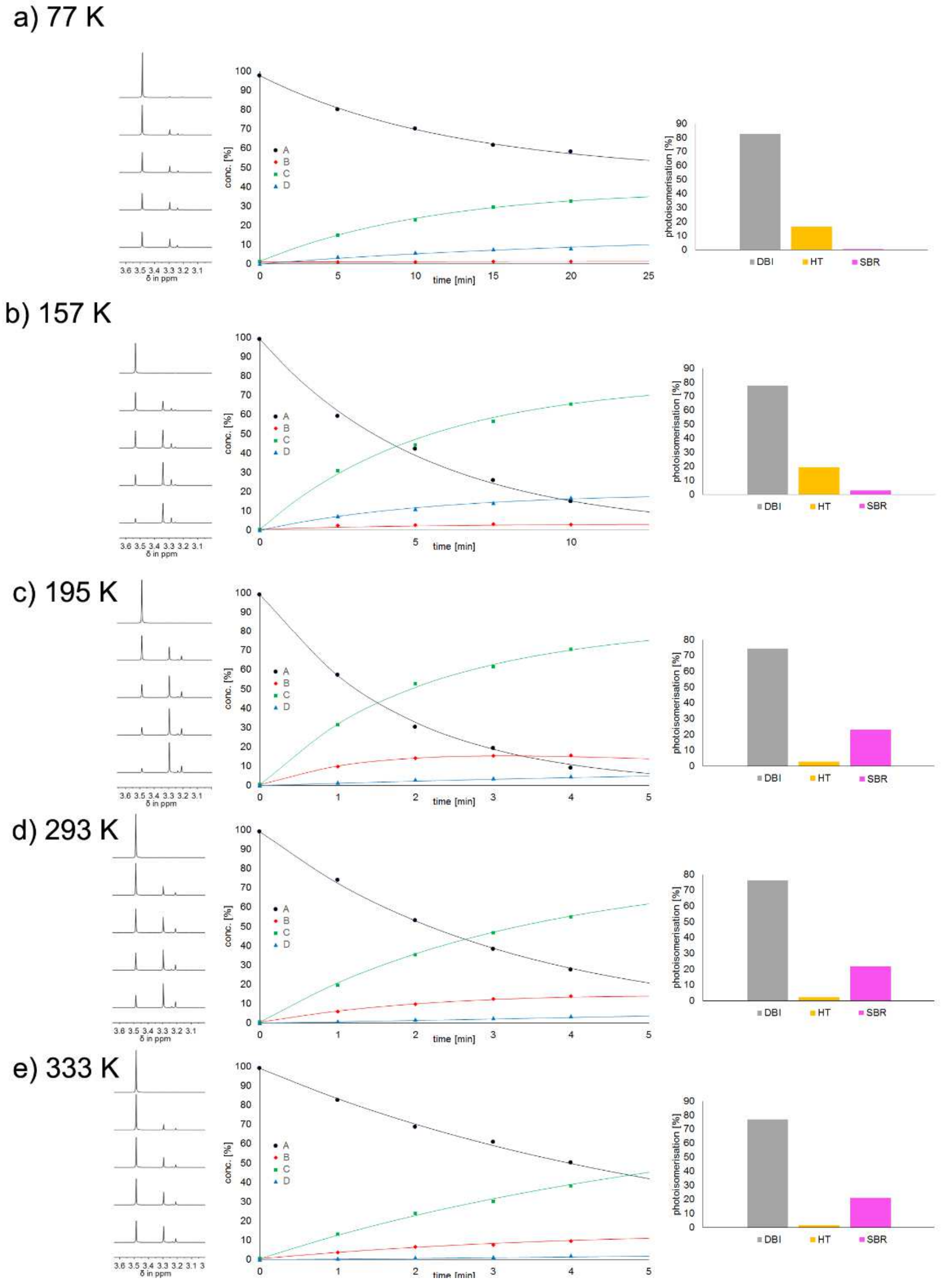

Supplementary Figure 49 | Photoreactions of A in toluene-d_8 at different temperatures. ^{1}H NMR spectra, Markov matrix analysis, and comparison of the different photoreactions (double-bond isomerization (DBI), hula twist (HT), single-bond rotation (SBR)) at a) 77 K (ice), b) 157 K (ice), c) 195 K (liquid), d) 293 K (liquid), and e) 333 K (liquid) under 405 nm illumination. The rates of the photoreactions at different temperatures cannot be compared with each other as the illumination conditions are not comparable at different temperatures. Kinetic plots of the experimentally determined changes in isomer composition during irradiation of **A-1** (dots) are fitted by a simplified Markov matrix (lines) using phototransition probabilities of **C-1 =** 0 and **D-1** = 0 since these transitions are much slower and therefore negligible in the experiments.

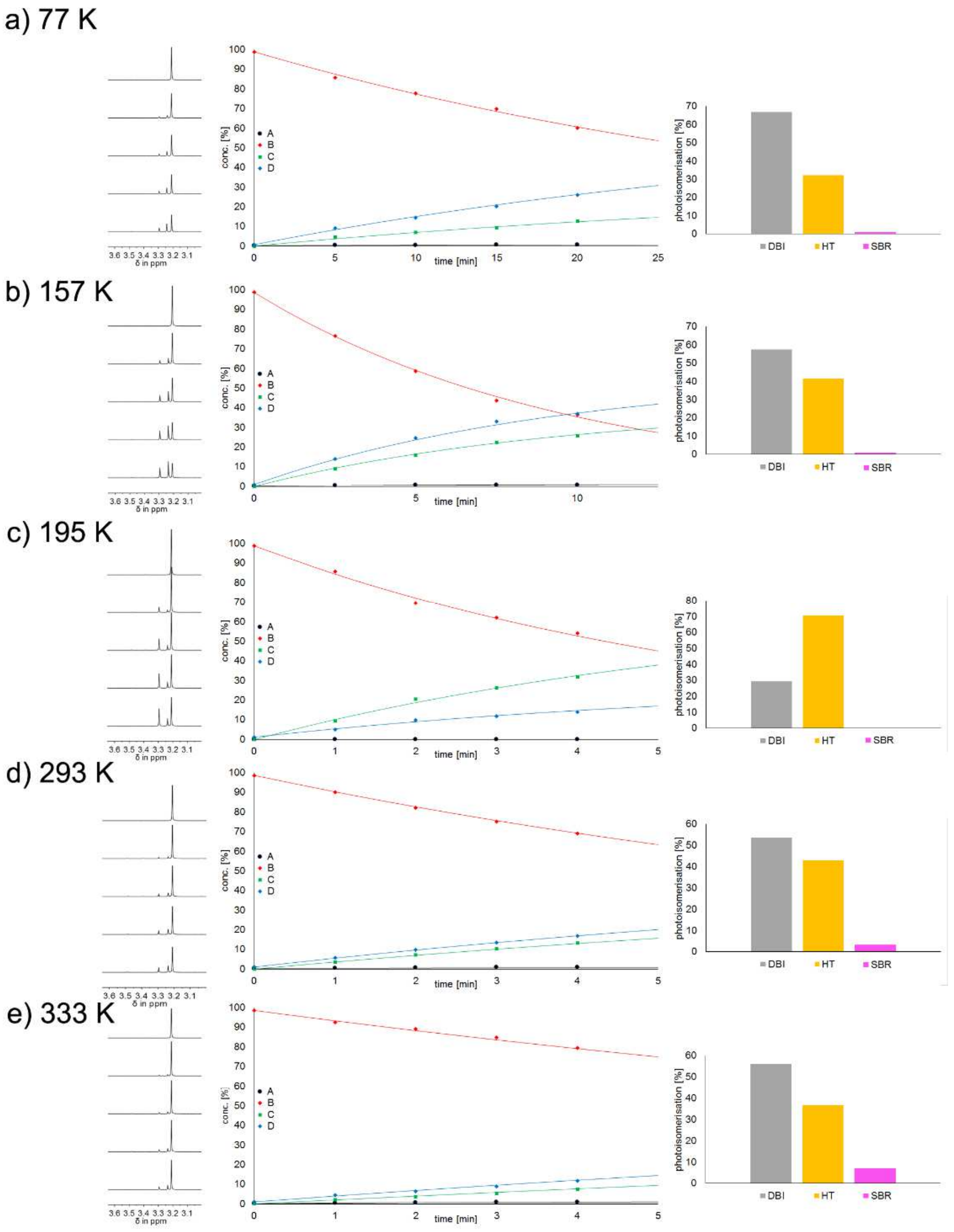

Supplementary Figure 50 | Photoreactions of B in toluene-d_8 at different temperatures. ^{1}H NMR spectra, Markov matrix analysis, and comparison of the different photoreactions (double-bond isomerization (DBI), hula twist (HT), single-bond rotation (SBR)) at a) 77 K (ice), b) 157 K (ice), c) 195 K (liquid), d) 293 K (liquid), and e) 333 K (liquid) under 405 nm illumination. The rates of the photoreactions at different temperatures cannot be compared with each other as the illumination conditions are not comparable at different temperatures. Kinetic plots of the experimentally determined changes in isomer composition during irradiation of **B-1** (dots) are fitted by a simplified Markov matrix (lines) using phototransition probabilities of **C-1 =** 0 and **D-1** = 0 since these transitions are much slower and therefore negligible in the experiments.

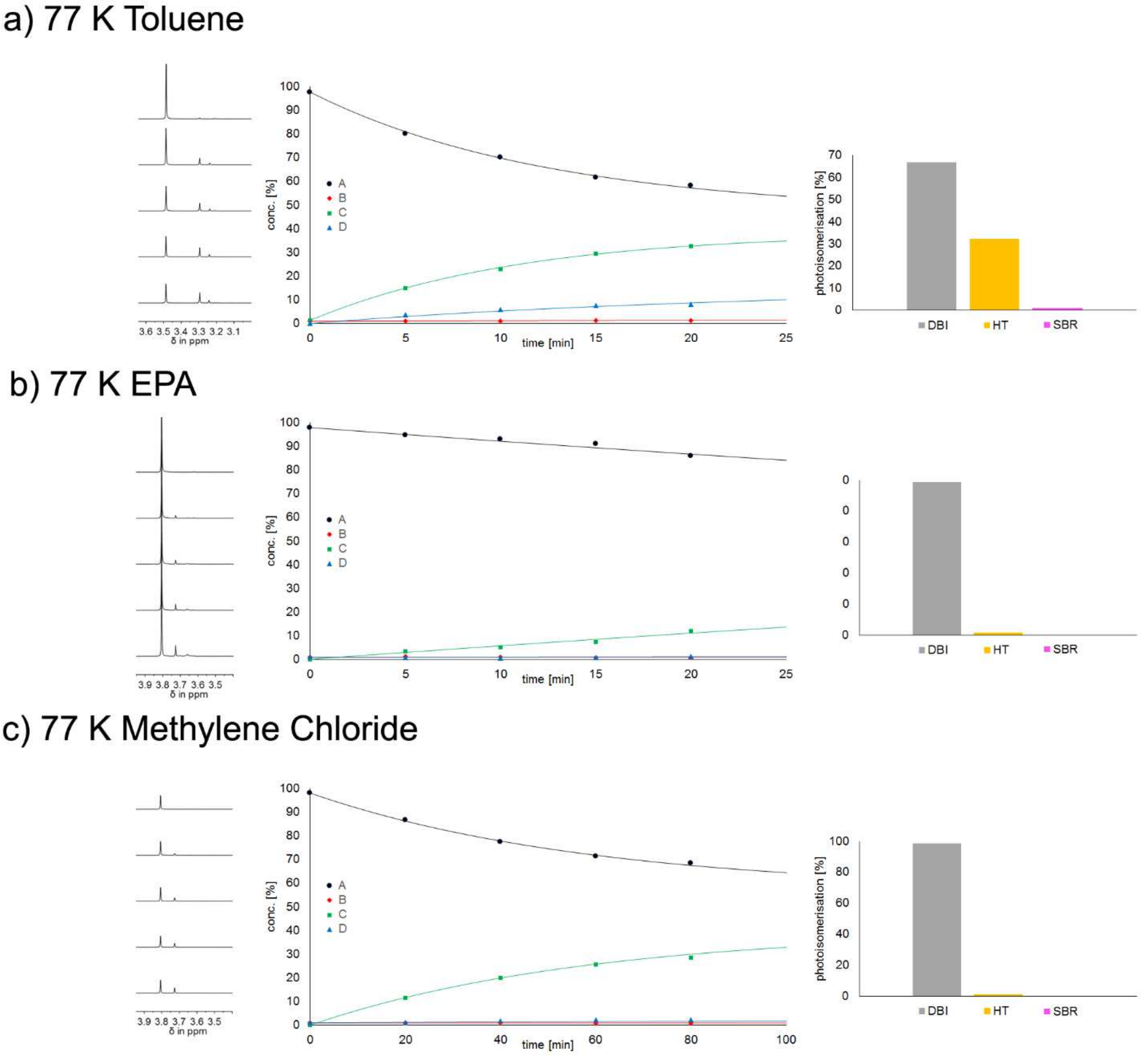

Supplementary Figure 51 | Photoreactions of A in different solvent ices at 77 K. ^{1}H NMR spectra, Markov matrix analysis, and comparison of the different photoreactions (double-bond isomerization (DBI), hula twist (HT), single-bond rotation (SBR)) at in a) toluene-d_8 (ice) at 77 K, b) EPA (glas) at 77 K, and c) CD_2Cl_2 (ice) at 77 K under 405 nm illumination. The EPA-solvent mixture was evaporated and the ^{1}H NMR spectra were recorded in CD_2Cl_2 after illumination. The rates of the photoreactions in different media cannot be compared with each other as the illumination conditions are not comparable. Kinetic plots of the experimentally determined changes in isomer composition during irradiation of **A-1** (dots) are fitted by a simplified Markov matrix (lines) with phototransition probabilities of **C-1** = 0 and **D-1** = 0 since these transitions are much slower and therefore negligible.

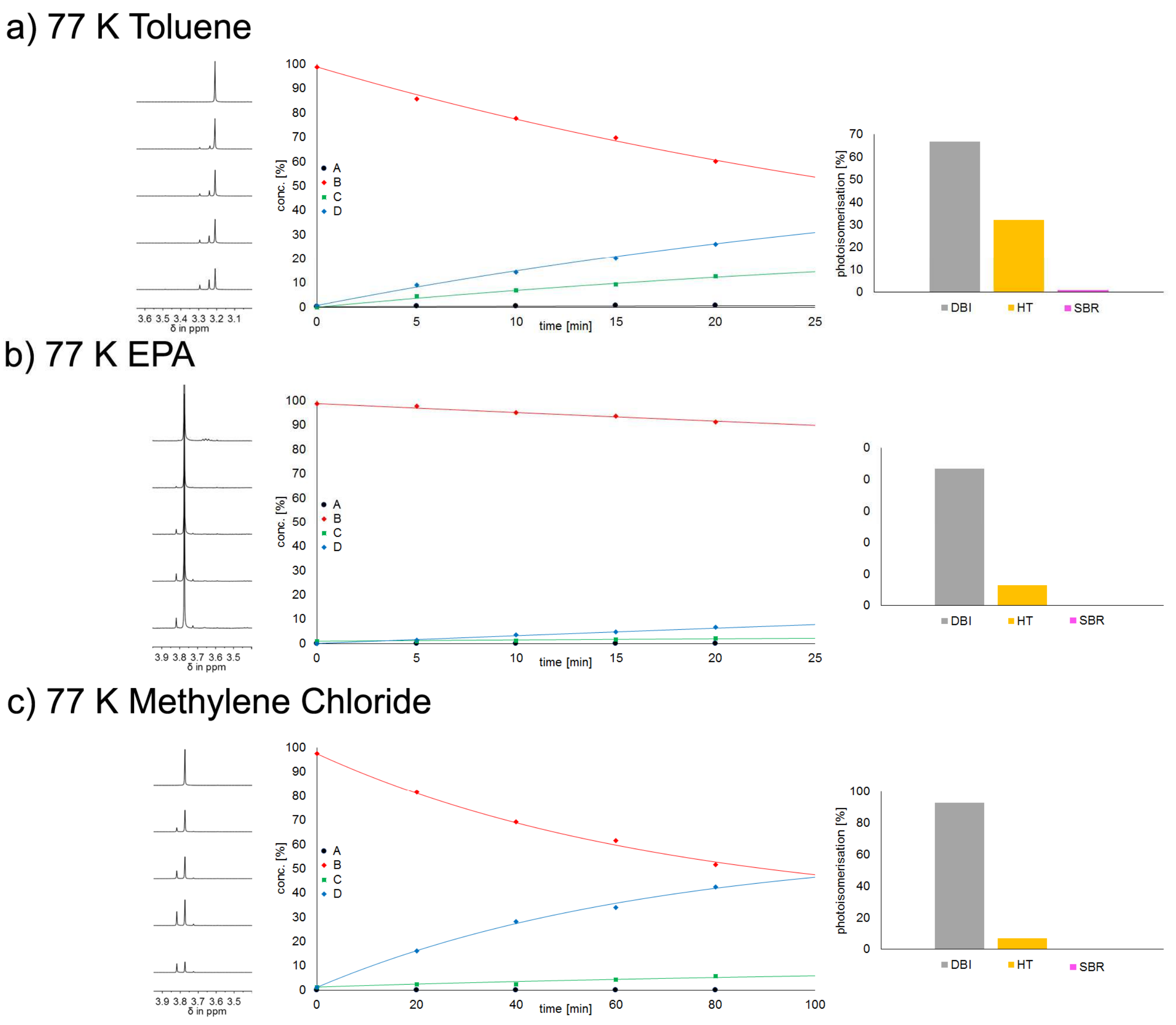

Supplementary Figure 52 | Photoreactions of B in different solvent ices at 77 K. ^{1}H NMR spectra, Markov matrix analysis, and comparison of the different photoreactions (double-bond isomerization (DBI), hula twist (HT), single-bond rotation (SBR)) at in a) toluene-*d*$_8$ (ice) at 77 K, b) EPA (glas) at 77 K, and c) CD_2Cl_2 (ice) at 77 K under 405 nm illumination. The EPA-solvent mixture was evaporated and the ^{1}H NMR spectra were recorded in CD_2Cl_2 after illumination. The rates of the photoreactions in different media cannot be compared with each other as the illumination conditions are not comparable. Kinetic plots of the experimentally determined changes in isomer composition during irradiation of **B-1** (dots) are fitted by a simplified Markov matrix (lines) with phototransition probabilities of **C-1** = 0 and **D-1** = 0 since these transitions are much slower and therefore negligible.

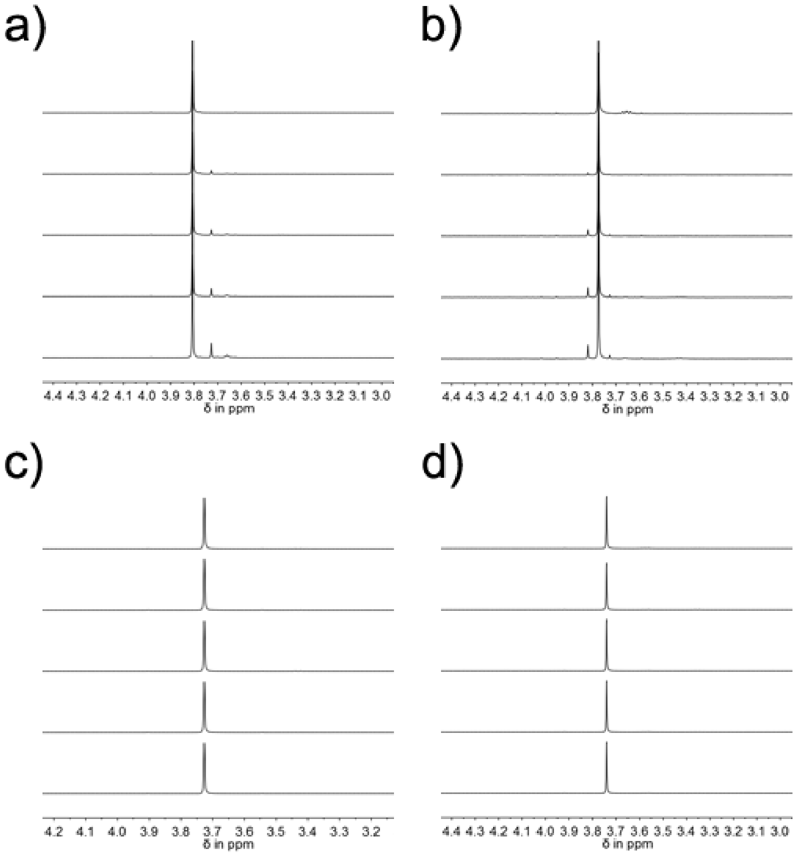

Supplementary Figure 53 | Comparison of the photoreaction kinetics of 1 in EPA matrices at 77 K. ^{1}H NMR spectra recorded after 405 nm irradiation of a) **A-1** (5 min intervals), (b) **B-1** (5 min intervals), c) **C-1** (20 min intervals), and d) **D-1** (20 min intervals). After illumination the EPA-solvent mixture was evaporated and the spectra were recorded in CD_2Cl_2. Similar illumination conditions were ensured by a hand-build setup with fixed sample and LED positions. For **C-1** and **D-1** no photoreaction was observed.

Comparison of the photoconversion of A and B under illumination with different wavelengths

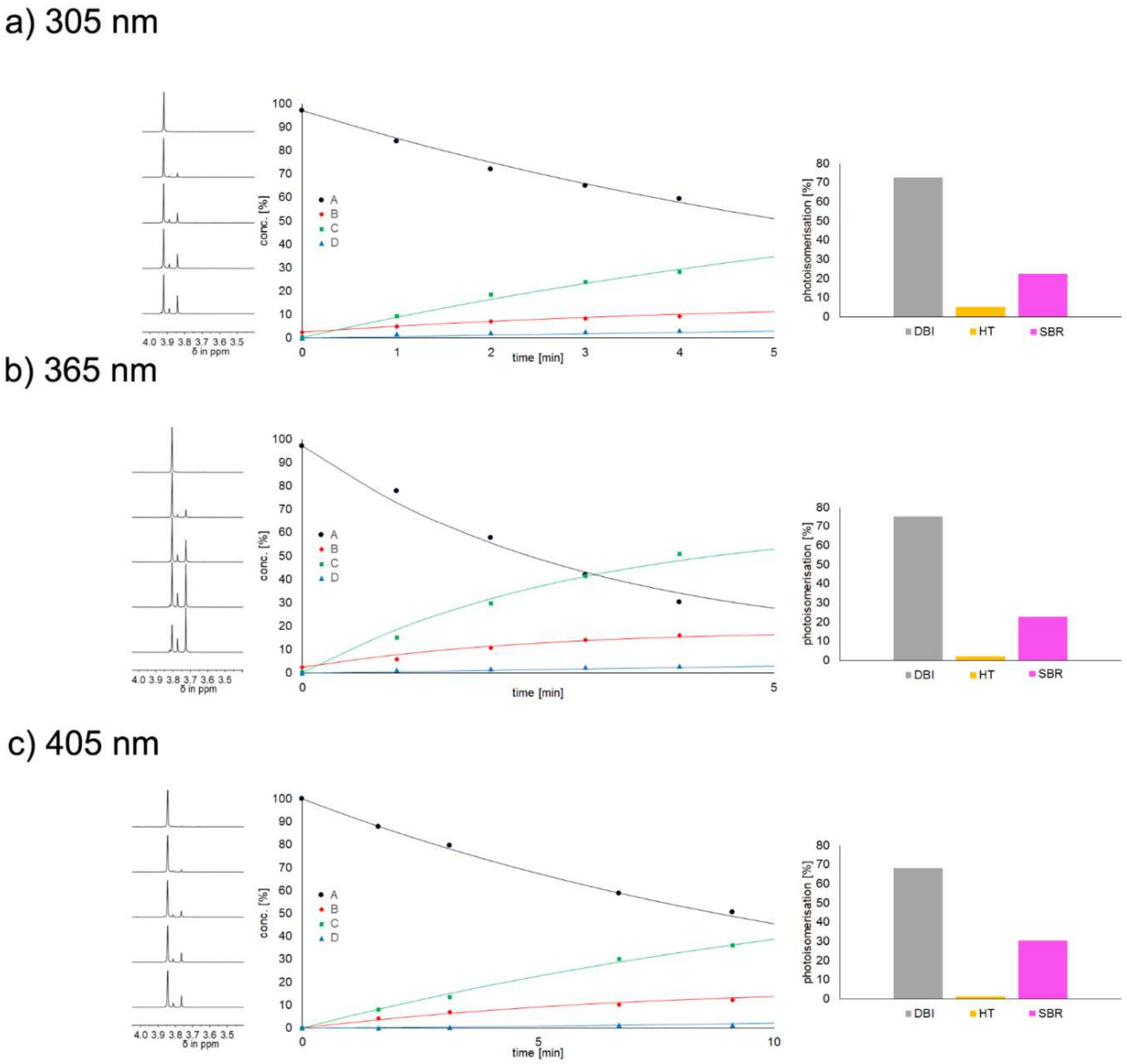

Supplementary Figure 54 | Photoreactions of A under illumination with different wavelength. Photoreactions (double-bond isomerization (DBI), hula twist (HT), single-bond rotation (SBR)) of **A-1** in CD_2Cl_2 at 20 °C using different irradiation wavelengths. ^{1}H NMR spectra, Markov matrix analysis, and comparison of the different photoreactions under a) 305 nm illumination, b) 365 nm illumination, and c) 405 nm illumination. Kinetic plots of the experimentally determined changes in isomer composition during irradiation of **A**-**1** (dots) are fitted by a simplified Markov matrix (lines) with phototransition probabilities of **C-1** = 0 and **D-1** = 0 since these transitions are much slower and therefore negligible.

a) 305 nm

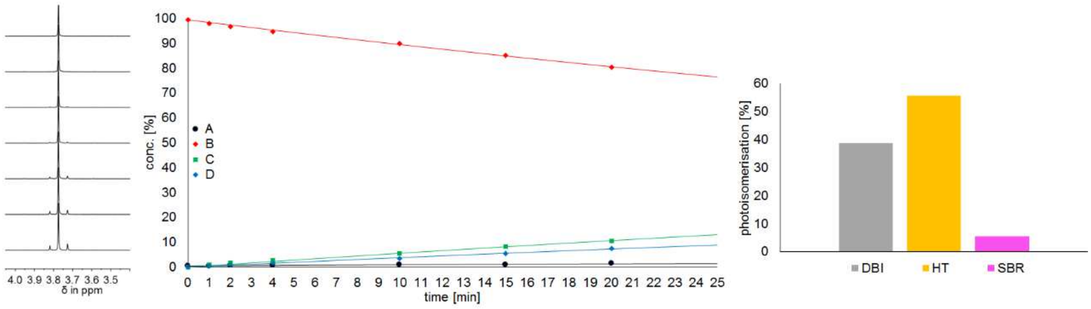

b) 365 nm

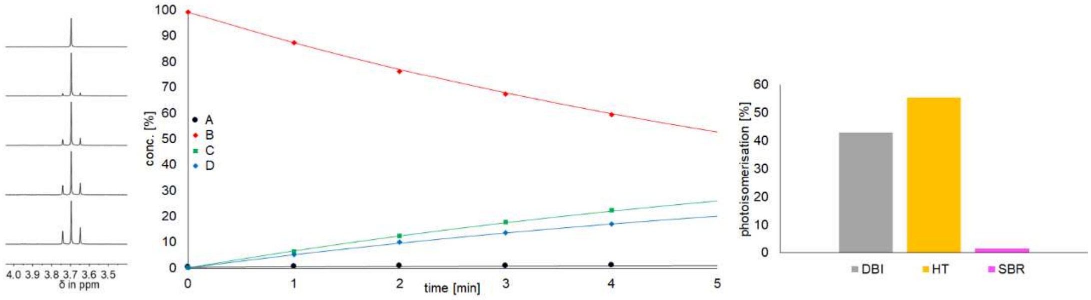

c) 405 nm

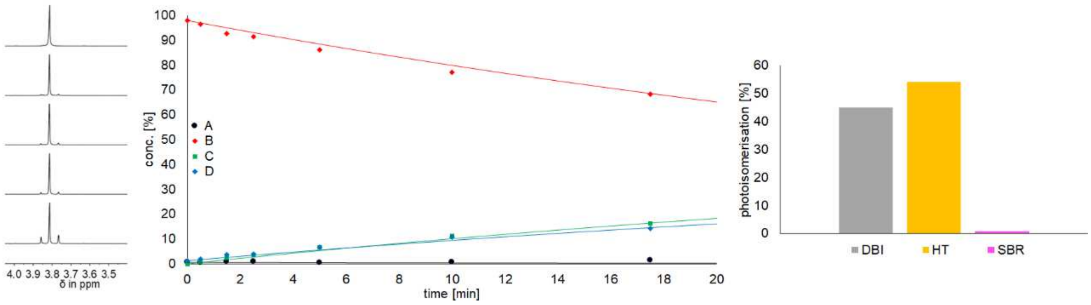

Supplementary Figure 55 | Photoreactions of B under illumination with different wavelength. Photoreactions (double-bond isomerization (DBI), hula twist (HT), single-bond rotation (SBR)) of **B-1** in CD_2Cl_2 at20 °C using different irradiation wavelengths. ^{1}H NMR spectra, Markov matrix analysis, and comparison of the different photoreactions under a) 305 nm illumination, b) 365 nm illumination, and c) 405 nm illumination. Kinetic plots of the experimentally determined changes in isomer composition during irradiation of **B**-**1** (dots) are fitted by a simplified Markov matrix (lines) with phototransition probabilities of **C-1 =** 0 and **D-1** = 0 since these transitions are much slower and therefore negligible.

Photoconversion of A, B, C and D in EPA glass at 90 K

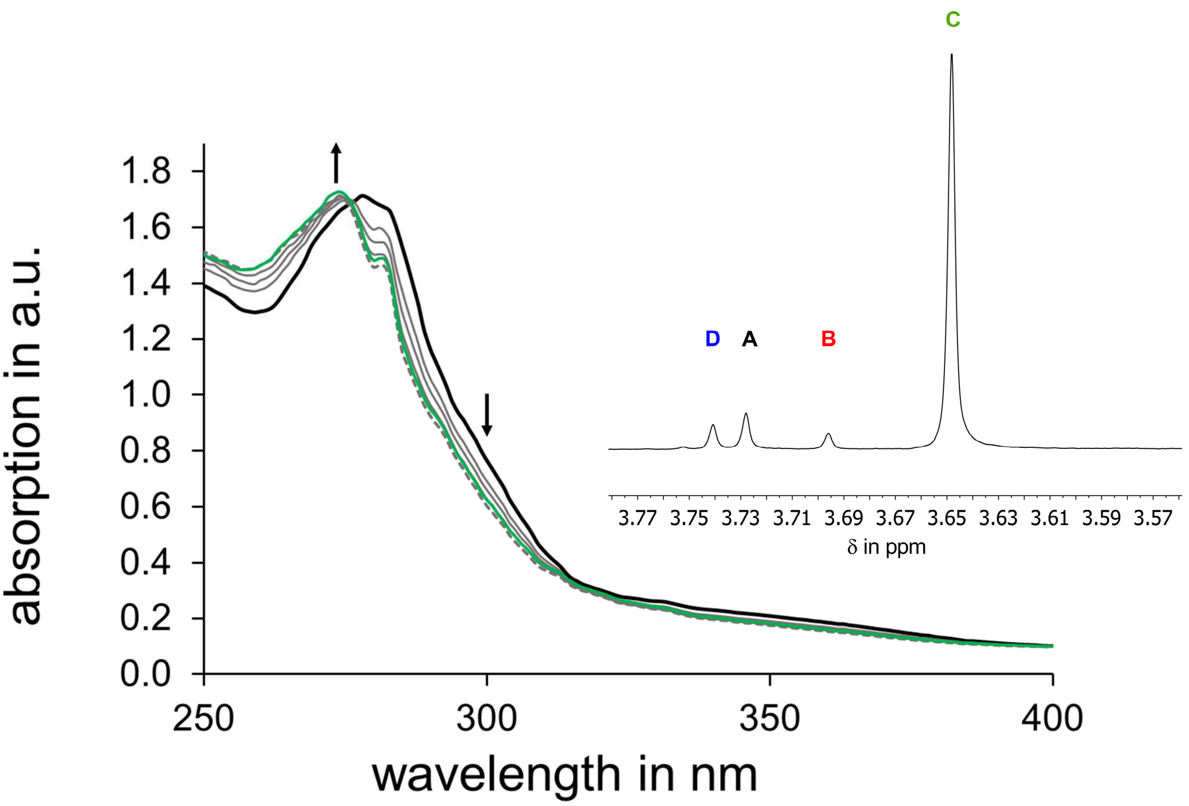

Supplementary Figure 56 | Photoconversion of A in EPA. UV/vis absorption spectra showing the photoconversion of **A** in EPA glass (diethyl ether/*iso*-pentane/ethanol 5:5:2) at 90 K. Starting from pure **A** (black) spectra were measured after 5, 10, 20 (grey lines), and 50 min (grey dashed line) of irradiation with a 405 nm ultra high power LED. After irradiation, the EPA glass was warmed to 195 K and cooled down again to 90 K (green). To determine the product distribution after irradiation the EPA solvent was evaporated, the resulting residue was dissolved in CD_2Cl_2 and a ^1H NMR spectrum (400 MHz, 27 °C) was recorded (inset). The main photoisomerization product can be assigned to **C** as shown in the NMR inset.

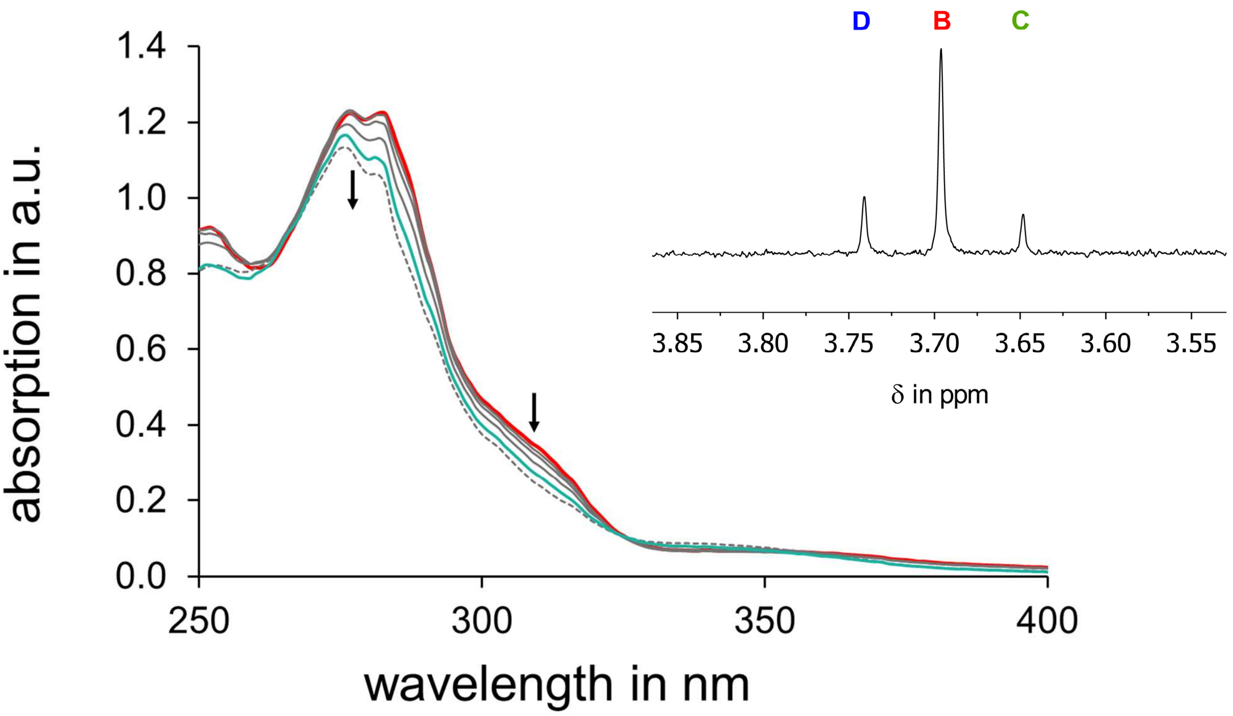

Supplementary Figure 57 | Photoconversion of B in EPA. UV/vis absorption spectra showing the photoconversion of **B** in EPA glass (diethyl ether/*iso*-pentane/ethanol 5:5:2) at 90 K. Starting from pure **B** (red) spectra were measured after 5, 10, 20 (grey lines), and 50 min (grey dashed line) of irradiation with a 405 nm ultra high power LED. After irradiation, the EPA glass was warmed to 195 K and cooled down again to 90 K (cyan). Changes in the UV/vis spectrum after warming and recooling can be assigned to diffusion effects and dilution of the spectum with unreacted **B**. To determine the product distribution after irradiation the EPA solvent was evaporated and the resulting residue was dissolved in CD_2Cl_2 and a ^1H NMR spectrum (400 MHz, 27 °C) was recorded (inset). The two photoisomerization products can be assigned to **C** and **D**, as shown in the NMR inset.

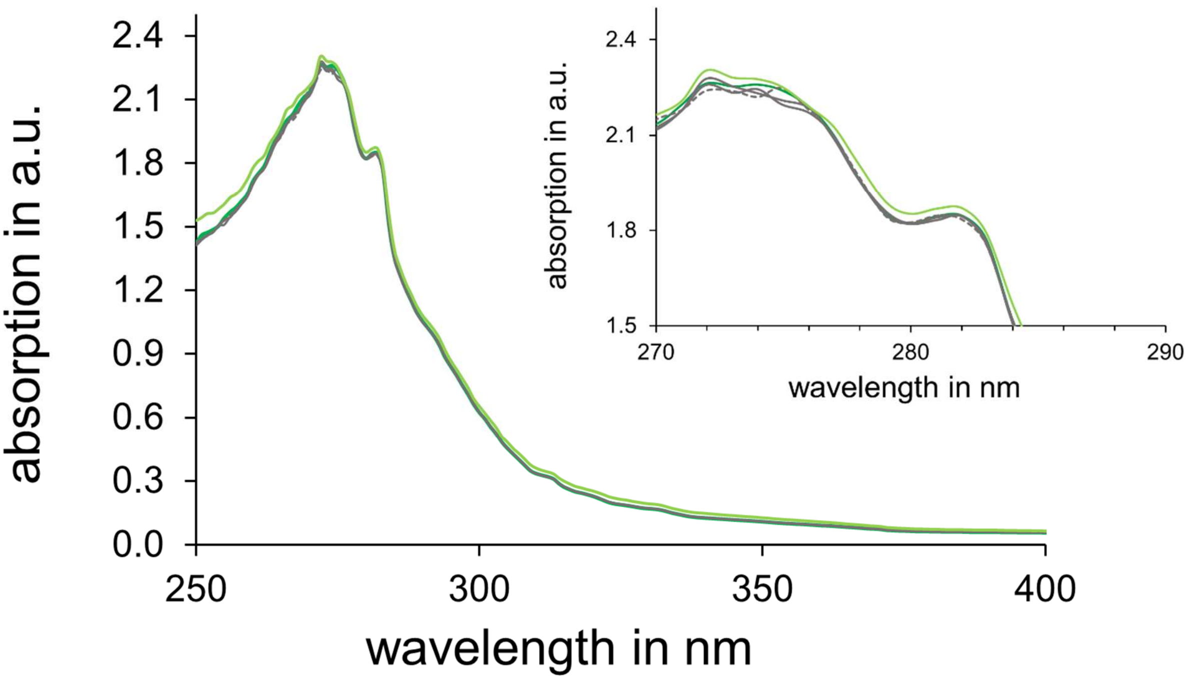

Supplementary Figure 58 | Photoconversion of C in EPA. UV/vis absorption spectra recorded during irradiation of **C** in EPA glass (diethyl ether/*iso*-pentane/ethanol 5:5:2) at 90 K. Starting from pure **C** (dark green) measurements were made after 30, 60, (grey lines), and 120 min (grey dashed line) of irradiation with a 405 nm ultra high power LED. After irradiation, the EPA glass was warmed to 195 K and cooled down again to 90 K (light green). As shown in the enlarged UV/vis absorption spectrum inset, no significant photoisomerization could be observed.

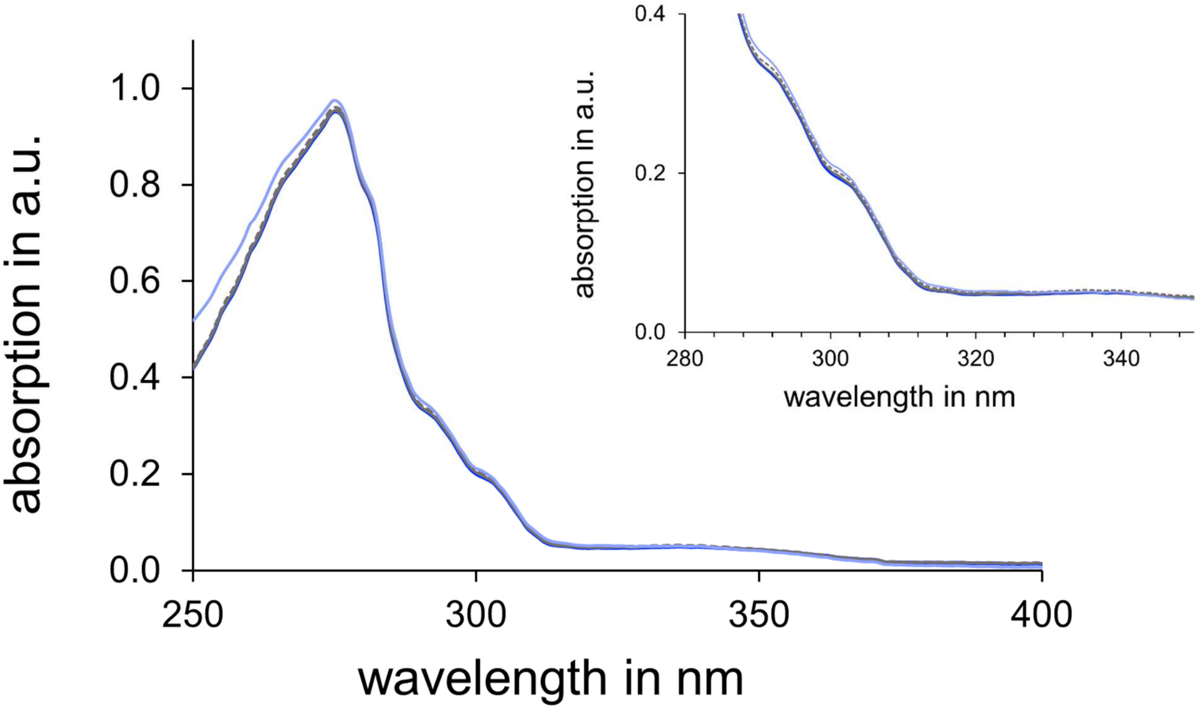

Supplementary Figure 59 | Photoconversion of D in EPA. UV/vis absorption spectra recorded during irradiation of **D** in EPA glass (diethyl ether/*iso*-pentane/ethanol 5:5:2) at 90 K. Starting from pure **D** (dark blue) measurements were made after 30, 60, (grey lines), and 120 min (grey dashed line) of irradiation with a 405 nm ultra high power LED. After irradiation, the EPA glass was warmed to 195 K and cooled down again to 90 K (light blue). As shown in the enlarged UV/vis absorption spectrum inset, no significant photoisomerization could be observed.

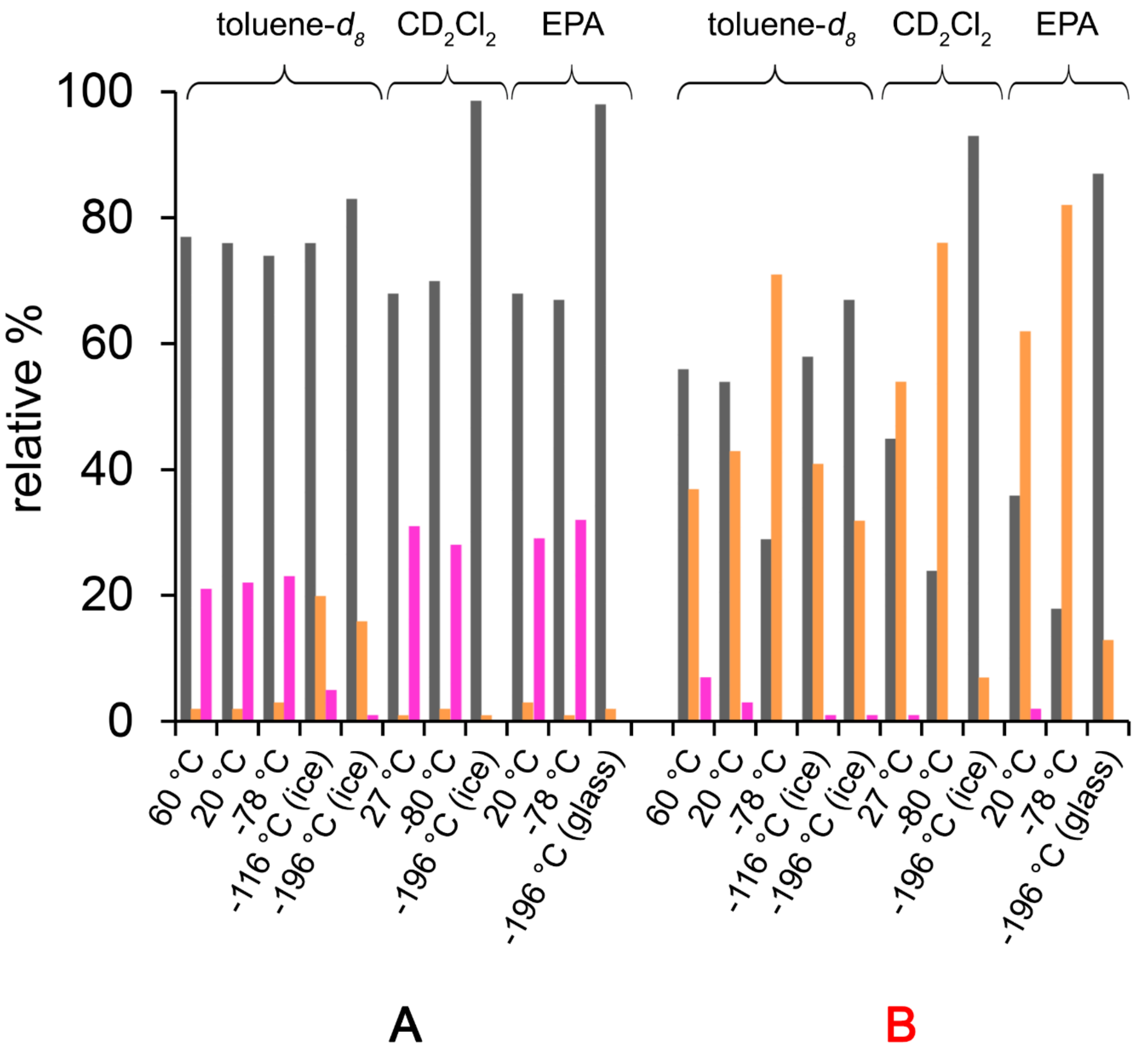

Supplementary Figure 60 | Relative quantum yields of 1 in different solvents and temperatures. Markov matrix analysis of the photoreactions of **1** in different solvents at different temperatures under 405 nm illumination. The relative efficiency of different processes is given. Measurements are conducted in liquid solutions at the indicated temperatures or in frozen solvent glasses or ices as indicated in brackets. The different photoprocesses are color coded: single-bond rotation (SBR) in purple, double-bond isomerization (DBI) in grey, and hula twist (HT) in orange.

NMR-Spectra

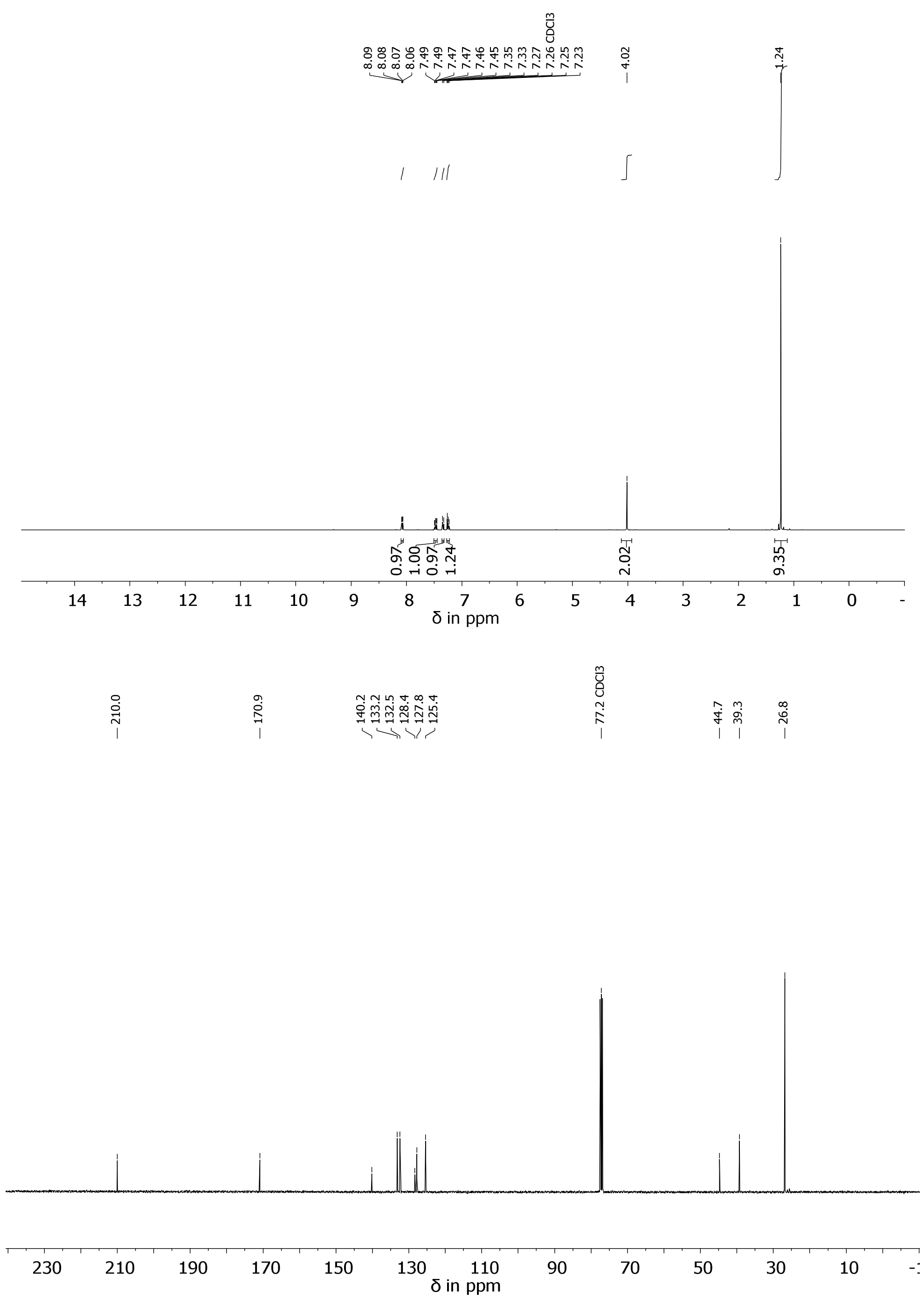

Supplementary Figure 61 | NMR spectra of 2. 400 MHz ^{1}H-NMR Spectrum (top) and 100 MHz ^{13}C-NMR Spectrum (bottom) of 2-((3,3-dimethyl-2-oxobutyl)thio)benzoic acid (**2**) in $CDCl_3$.

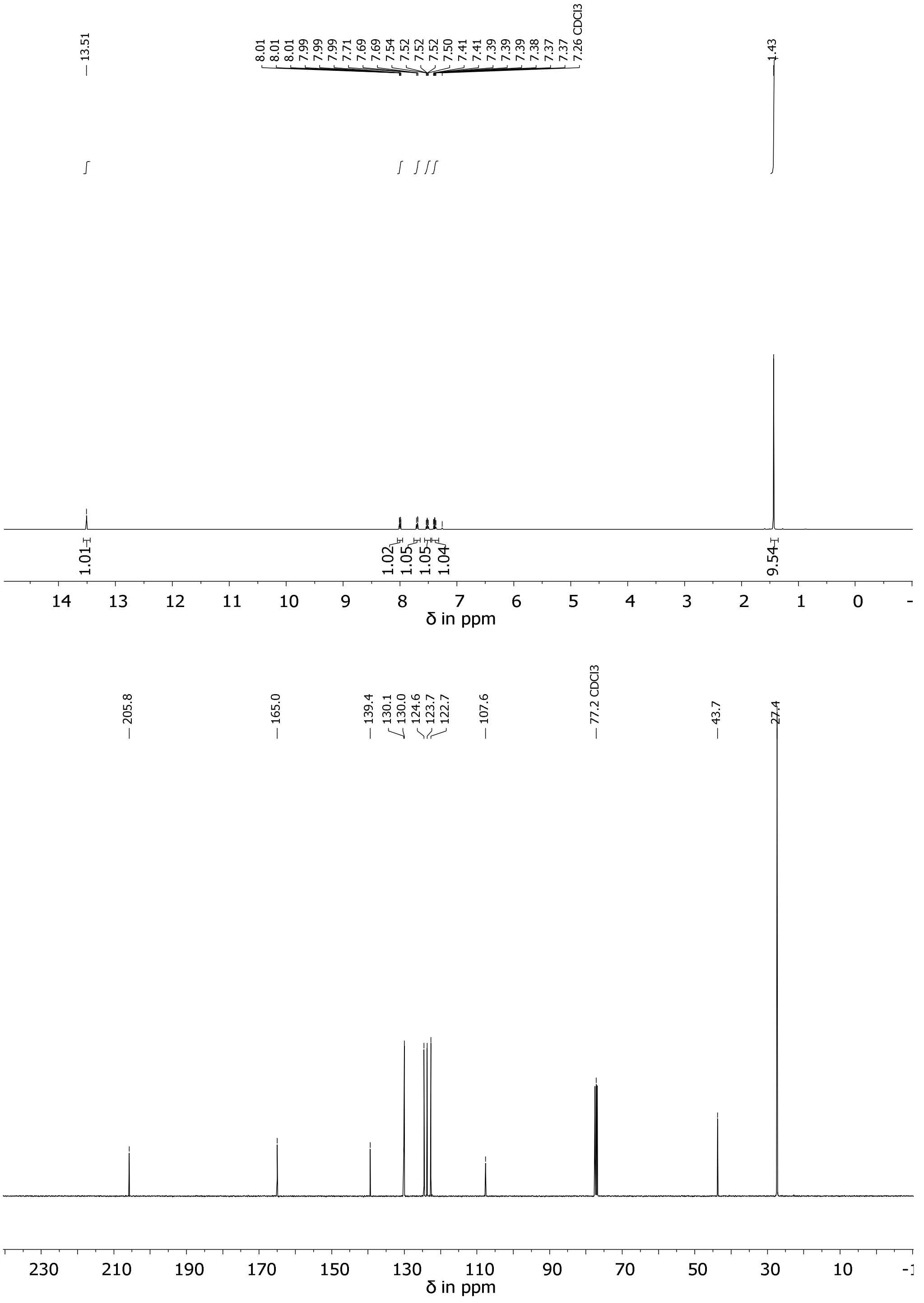

Supplementary Figure 62 | NMR spectra of 3. 400 MHz ^{1}H-NMR Spectrum (top) and 100 MHz ^{13}C-NMR Spectrum (bottom) of 1-(3-hydroxybenzo[b]thiophen-2-yl)-2,2-dimethylpropan-1-one (**3**) in $CDCl_3$.

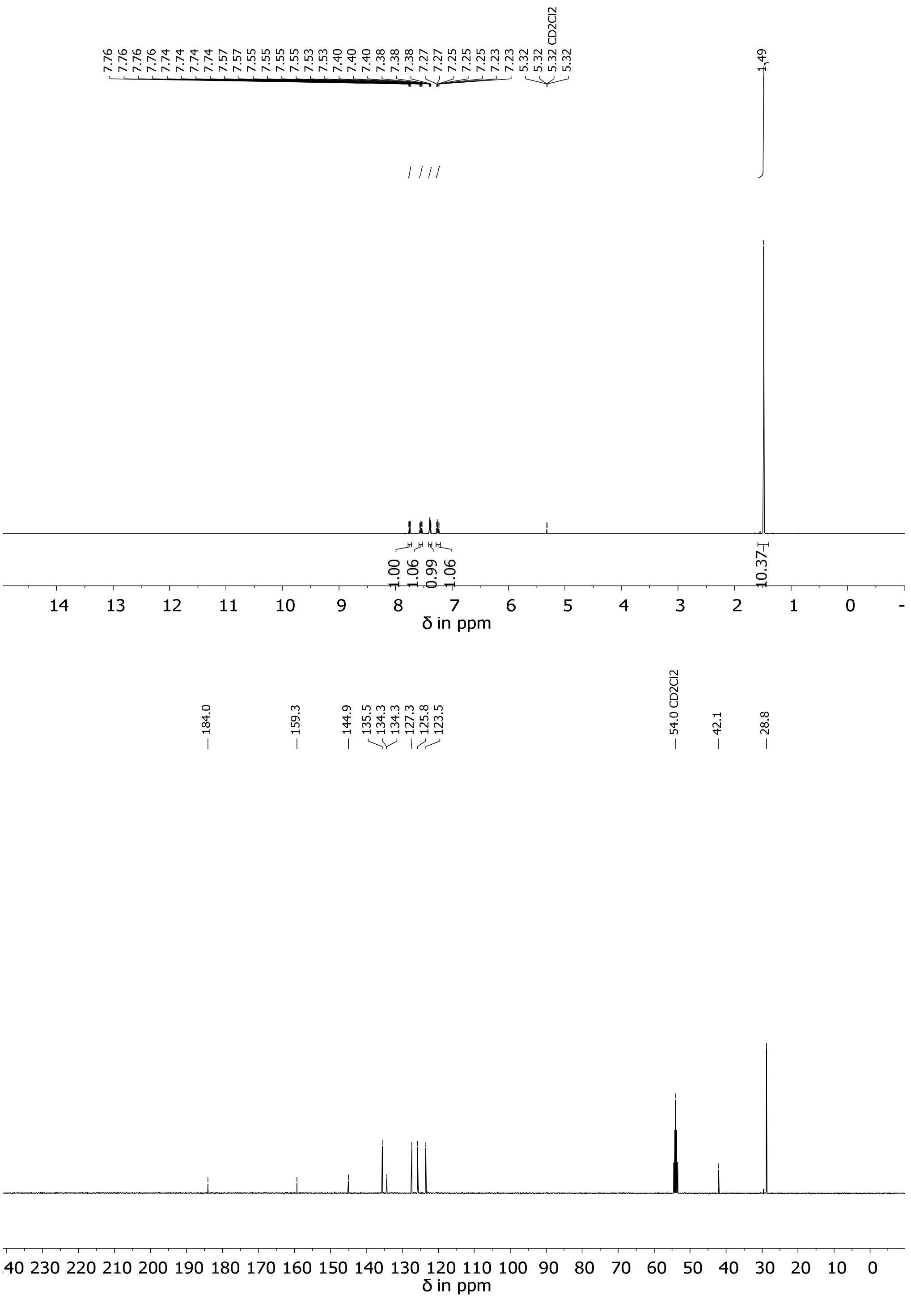

Supplementary Figure 63 | NMR spectra of 4. 400 MHz ^{1}H-NMR Spectrum (top) and 100 MHz ^{13}C-NMR Spectrum (bottom) of (*Z*)-2-(1-chloro-2,2-dimethylpropylidene)benzo[*b*]thiophen-3(2*H*)-one (**4**) in CD_2Cl_2.

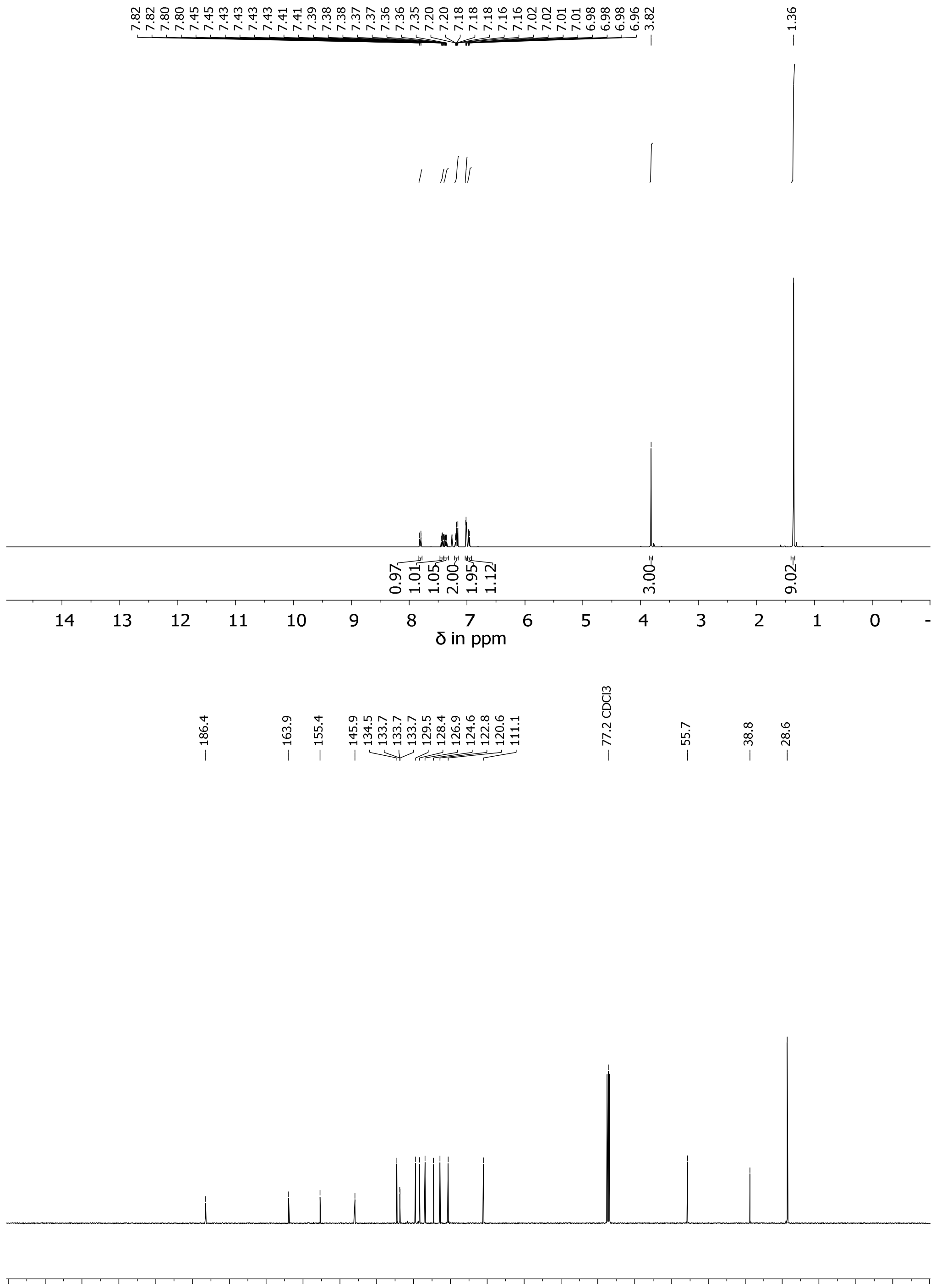

Supplementary Figure 64 | NMR spectra of 5. 400 MHz ^{1}H-NMR Spectrum (top) and 100 MHz ^{13}C-NMR Spectrum (bottom) of (*Z*)-2-(1-(2-Methoxyphenyl)-2,2-dimethylpropylidene)benzo[*b*]thiophen-3(2*H*)-one (**5**) in $CDCl_3$.

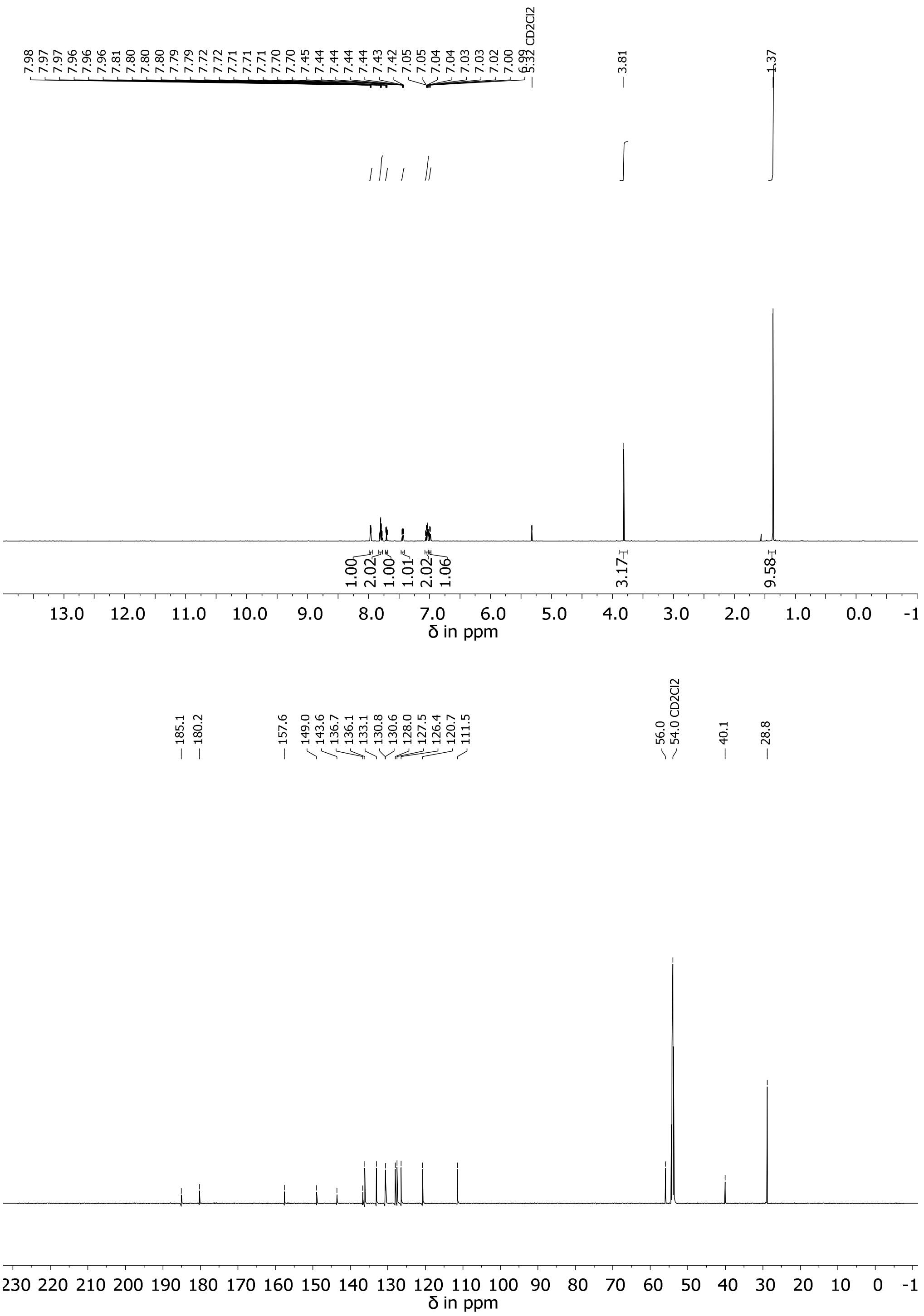

Supplementary Figure 65 | NMR spectra of A. 600 MHz ^{1}H-NMR Spectrum (top) and 150 MHz ^{13}C-NMR Spectrum (bottom) of the **A** isomer of 2-(1-(2-methoxyphenyl)-2,2-dimethylpropylidene)benzo[*b*]thiophen-3(2*H*)-one 1-oxide (**1**) in CD_2Cl_2.

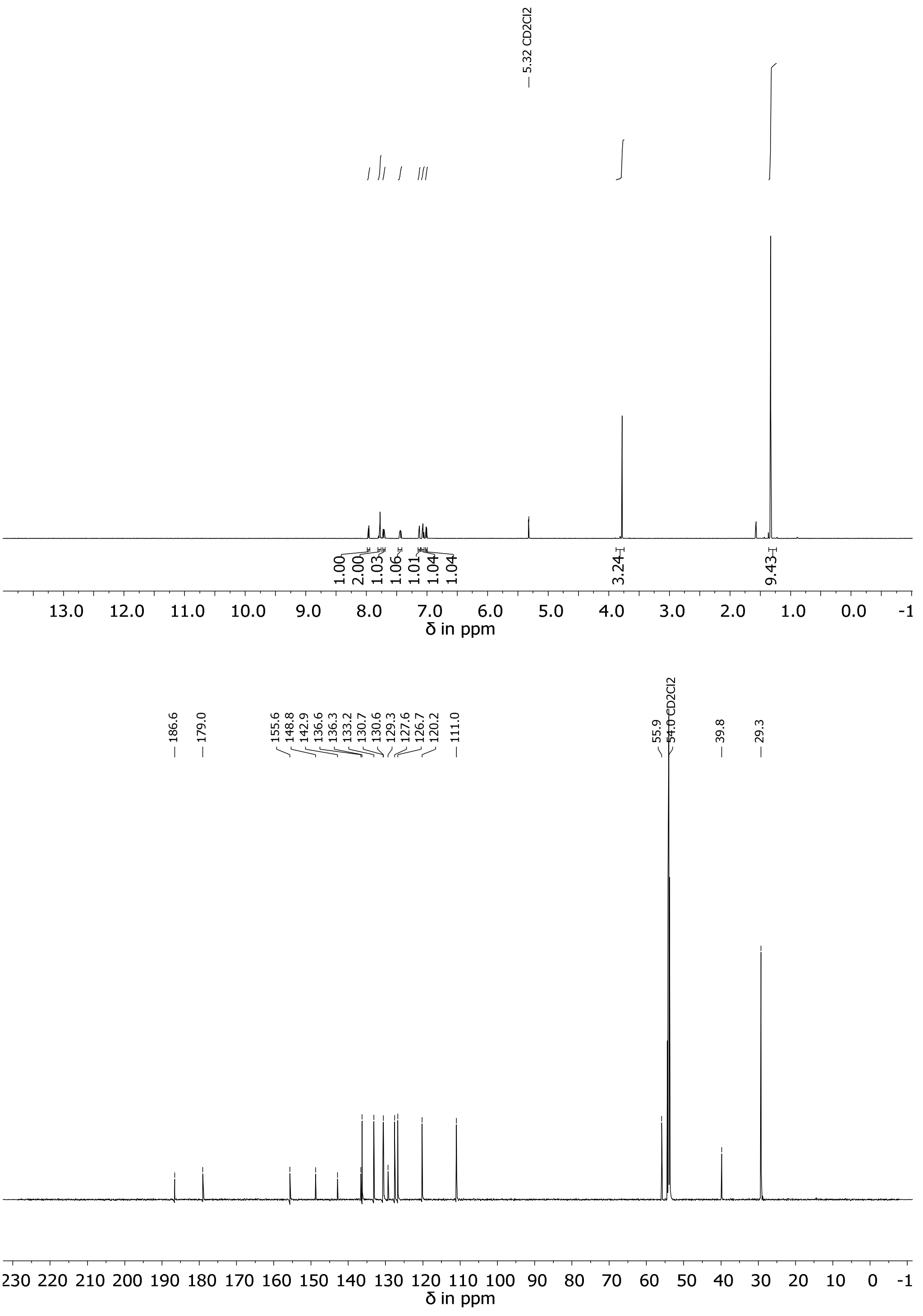

Supplementary Figure 66 | NMR spectra of B. 600 MHz ^{1}H-NMR Spectrum (top) and 150 MHz ^{13}C-NMR Spectrum (bottom) of the **B** isomer of 2-(1-(2-methoxyphenyl)-2,2-dimethylpropylidene)benzo[*b*]thiophen-3(2*H*)-one 1-oxide in CD_2Cl_2.

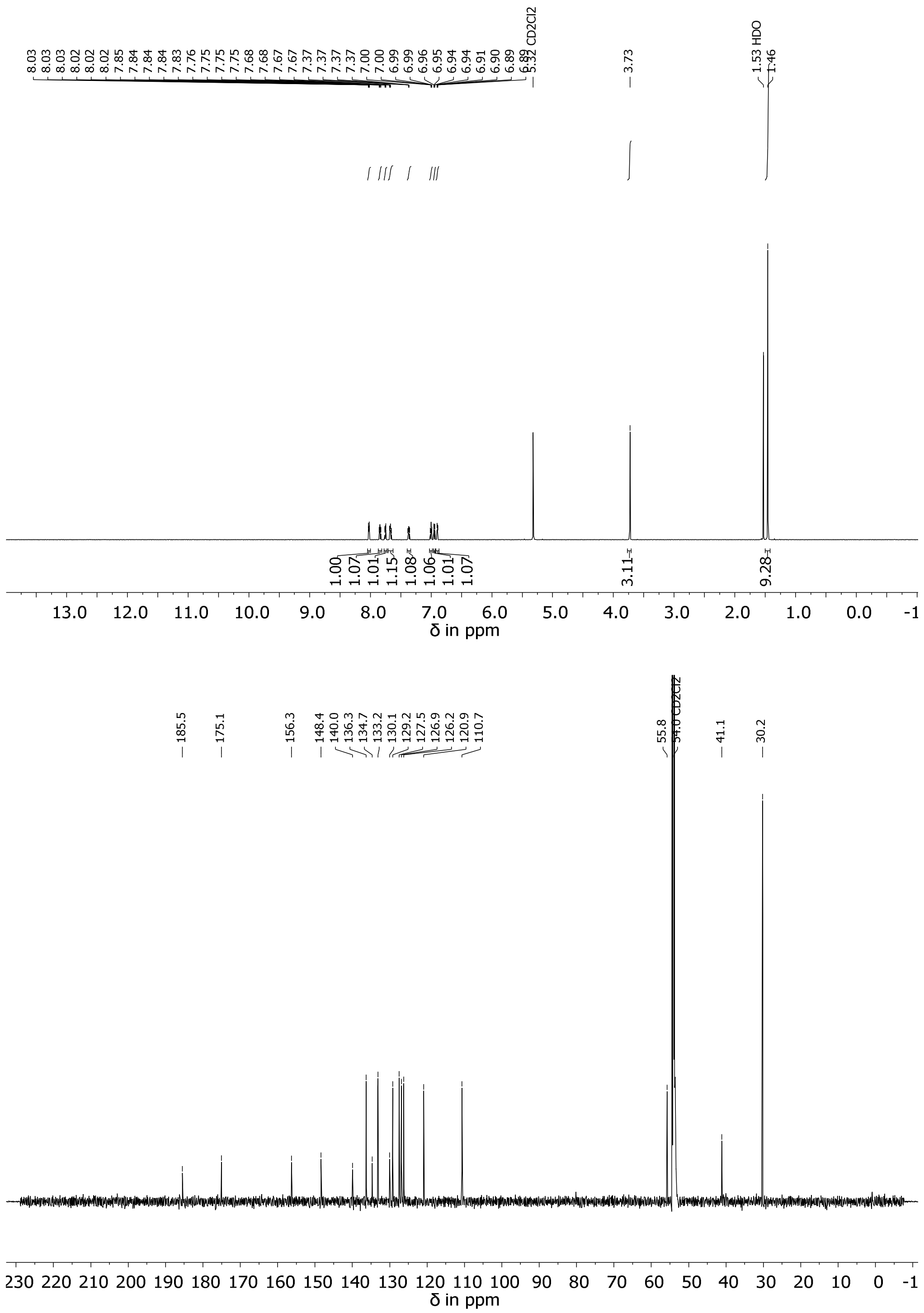

Supplementary Figure 67 | NMR spectra of C. 600 MHz ^{1}H-NMR Spectrum (top) and 150 MHz ^{13}C-NMR Spectrum (bottom) of the **C** isomer of 2-(1-(2-methoxyphenyl)-2,2-dimethylpropylidene)benzo[*b*]thiophen-3(2*H*)-one 1-oxide in CD_2Cl_2.

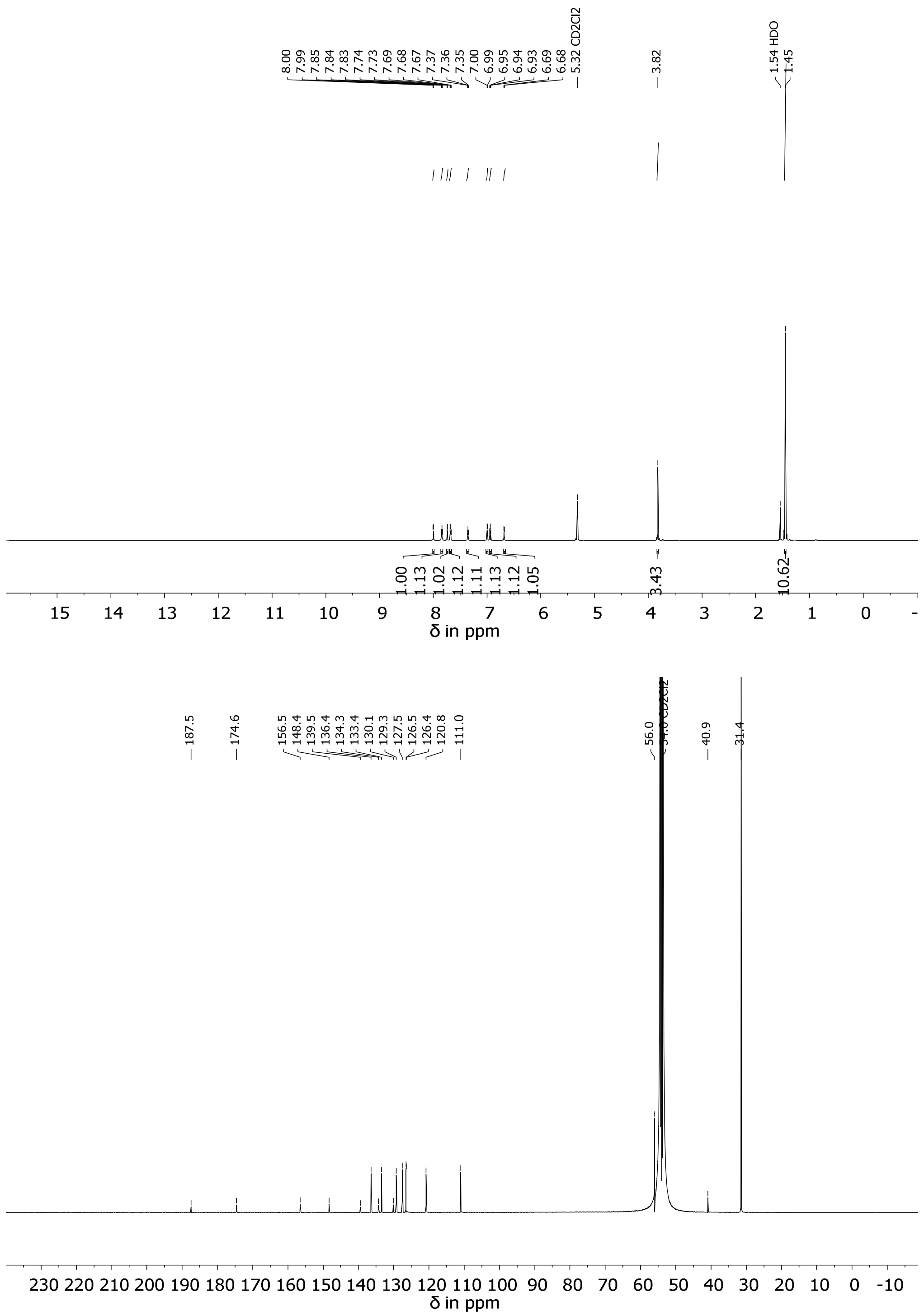

Supplementary Figure 68 | NMR spectra of D. 800 MHz ^{1}H-NMR Spectrum (top) and 200 MHz ^{13}C-NMR Spectrum (bottom) of the **D** isomer of 2-(1-(2-methoxyphenyl)-2,2-dimethylpropylidene)benzo[*b*]thiophen-3(2*H*)-one 1-oxide in CD_2Cl_2.

Supplementary Tables

Supplementary Table 1 | Thermal isomerization of 1. Thermal isomerization behavior of hemithioindigo 1 in $(CDCl_2)_2$ at high temperature.

Isomer	$k_{(\mathbf{isom.\,1/isom.\,2})}$ / s^{-1} (at T / °C)	ΔG^* (therm. isomer equilibration) /kcal mol^{-1}	Equilibra-tion half-life of pure isomer at 27 °C	Thermo-dynamic isomer 1/isomer 2 equilibrium in the dark (at T / °C)	$\Delta\Delta G^0$(isomer 1 / isomer 2) / kcal mol^{-1}	Slope m / s^{-1} (at T / °C)
**A**	$k_{(\mathbf{A/B})}$ = 2.47x10^{-5} (82 °C)	ΔG^*(**A**/**B**) = 28.40	1.7 a	**A**/**B** = 49/51 (82 °C)	$\Delta\Delta G^0$(**A**/**B**) = -0.04	5.07x10^{-5} (82 °C)
**B**	$k_{(\mathbf{B/A})}$ = 2.82x10^{-5} (82 °C)	ΔG^*(**B**/**A**) = 27.93	0.8 a	**B**/**A** = 52/48 (82 °C)	$\Delta\Delta G^0$(**B**/**A**) = 0.04	9.09x10^{-5} (82 °C)
**C**	$k_{(\mathbf{C/D})}$ = 2.74x10^{-5} (100 °C)	ΔG^*(**C**/**D**) = 29.80	18 a	**C**/**D** = 69/31 (100 °C)	$\Delta\Delta G^0$(**C**/**D**) = -0.65	8.98x10^{-5} (100 °C)
**D**	$k_{(\mathbf{D/C})}$ = 5.67x10^{-5} (100 °C)	ΔG^*(**D**/**C**) = 29.26	7.3 a	**D**/**C** = 29/71 (100 °C)	$\Delta\Delta G^0$(**D**/**C**) = 0.65	7.94x10^{-5} (100 °C)

Supplementary Table 2 | Relative quantum yields of 1. Relative percentage of different photoconversions of **1** in different solvents *obtained from Markov matrix analysis according to Supplementary Equation 28 or **from direct quantum yield measurements. SBR = single-bond rotation, DBI = double-bond isomerization, HT = hula twist, EG = ethylene glycol.

Pathway %	ϕ Measurement	Markov Matrix Analysis				
		Solvent				
	Benzene**	Benzene*	CH_2Cl_2*	MeOH*	DMSO*	EG*
**A to B SBR**	22	22	31	42	33	30
**A to C DBI**	76	76	68	58	62	63
**A to D HT**	2	2	0	0	5	7
**B to A SBR**	5	0	0	0	0	0
**B to C HT**	42	47	55	66	52	57
**B to D DBI**	51	53	45	34	48	43

Supplementary Table 3 | Relative quantum yields of A and B at different temperatures. Relative percentage of different photoconversions of **1** in different solvents and temperatures obtained from Markov matrix analysis according to Supplementary Equation 28. SBR = single-bond rotation, DBI = double-bond isomerization, HT = hula twist.

Solvent	Toluene-d_8					CD_2Cl_2			EPA		
T in °C	60	20	- 78	- 116	- 196	27	- 80	- 196	20	- 78	- 196
**A to B SBR**	21	22	23	5	1	31	28	0	29	32	0
**A to C DBI**	77	76	74	76	83	68	70	99	68	67	98
**A to D HT**	2	2	3	20	16	1	2	1	3	1	2
**B to A SBR**	7	3	0	1	1	1	0	0	2	0	0
**B to C HT**	37	43	71	41	32	54	76	7	62	82	13
**B to D DBI**	56	54	29	58	67	45	24	93	36	18	87

Supplementary Table 4 | Relative quantum yields of A and B under illumination with different wavelengths. Relative percentage of different photoconversions of **1** under different illumination conditions in CD_2Cl_2 at 20 °C obtained from Markov matrix analysis according to Supplementary Equation 28. SBR = single-bond rotation, DBI = double-bond isomerization, HT = hula twist.

Wavelength	305 nm	365 nm	405 nm
**A to B SBR**	22	23	31
**A to C DBI**	72	75	68
**A to D HT**	5	2	1
**B to A SBR**	6	1	1
**B to C HT**	56	56	54
**B to D DBI**	39	53	45

Calculated ground state geometries - xyz coordinates

Supplementary Table 5 | Calculated ground state geometries of *E*-(*S*)-(R_a)-1 and *E*-(*S*)-(S_a)-1.

E-(*S*)-(R_a)-**1**				*E*-(*S*)-(S_a)-**1**			
S	-1.9046	1.4644	0.3482	S	1.8785	1.5237	0.0476
O	-0.2507	-2.0933	0.0158	O	0.3187	-2.0622	0.4225
O	-2.1220	2.4021	-0.8335	O	1.9911	2.0752	1.4666
C	-3.0529	0.0798	0.1656	C	3.0568	0.1481	-0.0552
C	-4.4357	0.2157	0.1697	C	4.4305	0.3107	-0.1712
H	-4.9070	1.1871	0.2579	H	4.8764	1.2932	-0.2707
C	-5.2007	-0.9441	0.0548	C	5.2237	-0.8375	-0.1538
H	-6.2819	-0.8714	0.0516	H	6.2990	-0.7445	-0.2493
C	-4.5910	-2.2016	-0.0506	C	4.6479	-2.1060	-0.0140
H	-5.2078	-3.0884	-0.1324	H	5.2842	-2.9828	-0.0041
C	-3.2058	-2.3177	-0.0537	C	3.2693	-2.2487	0.1102
H	-2.7158	-3.2800	-0.1417	H	2.8067	-3.2222	0.2213
C	-2.4358	-1.1571	0.0454	C	2.4731	-1.1040	0.0862
C	-0.9550	-1.1020	0.0214	C	0.9918	-1.0786	0.1881
C	-0.4802	0.3265	0.0480	C	0.4839	0.3177	-0.0547
C	0.8026	0.7324	-0.1135	C	-0.7938	0.6519	-0.3588
C	1.8104	-0.3055	-0.5178	C	-1.7880	-0.4617	-0.5119
C	2.6008	-0.9531	0.4501	C	-2.6465	-0.8054	0.5477
C	3.5632	-1.8829	0.0580	C	-3.5884	-1.8213	0.3830
C	3.7359	-2.1772	-1.2963	C	-3.6788	-2.4945	-0.8366
C	2.9534	-1.5530	-2.2591	C	-2.8374	-2.1612	-1.8910
H	3.0803	-1.7856	-3.3096	H	-2.9057	-2.6835	-2.8377
C	1.9912	-0.6226	-1.8614	C	-1.8948	-1.1466	-1.7198
C	1.3312	2.1871	-0.0425	C	-1.3161	2.0910	-0.6289
H	1.3655	-0.1425	-2.6047	H	-4.2487	-2.0961	1.1941
H	4.1741	-2.3865	0.7945	H	-1.2271	-0.8865	-2.5336
C	1.0678	2.8486	-1.4227	C	-1.1212	2.9740	0.6280
H	1.4454	3.8753	-1.4016	H	-1.5103	3.9756	0.4233
H	1.5951	2.3169	-2.2186	H	-1.6767	2.5541	1.4690
H	0.0023	2.8669	-1.6507	H	-0.0796	3.0660	0.9315
C	2.8557	2.2171	0.2163	C	-2.8235	2.0985	-0.9625
H	3.1827	3.2594	0.2310	H	-3.0514	1.5189	-1.8587
H	3.1070	1.7676	1.1783	H	-3.4231	1.7098	-0.1384
H	3.4168	1.7026	-0.5642	H	-3.1325	3.1313	-1.1424
C	0.6696	3.0212	1.0775	C	-0.5773	2.6996	-1.8476
H	-0.3557	3.3080	0.8501	H	0.4904	2.8171	-1.6718
H	0.6865	2.4900	2.0321	H	-0.7121	2.0767	-2.7359
H	1.2318	3.9505	1.1990	H	-0.9949	3.6868	-2.0624
O	2.3442	-0.6100	1.7449	O	-2.4800	-0.0868	1.6946
C	3.0804	-1.2556	2.7839	C	-3.2847	-0.4087	2.8293
H	2.7004	-0.8415	3.7157	H	-3.1186	-1.4412	3.1502
H	2.9146	-2.3368	2.7716	H	-2.9679	0.2708	3.6178
H	4.1513	-1.0463	2.7028	H	-4.3476	-0.2543	2.6206
H	4.4864	-2.9032	-1.5878	H	-4.4142	-3.2827	-0.9520

Supplementary Table 6 | Calculated ground state geometries of Z-(*S*)-(R_a)-1 and Z-(*S*)-(S_a)-1.

Z-(*S*)-(R_a)-**1**				*Z*-(*S*)-(S_a)-**1**			
S	-0.9028	-1.3331	-0.2487	S	0.8714	-1.3220	-0.4538
O	-1.8798	2.4954	0.0350	O	1.8563	2.4923	-0.3566
O	-0.4838	-2.0189	1.0438	O	0.9245	-1.7464	-1.9165
C	-2.6710	-0.9676	-0.1081	C	2.5876	-0.9654	0.0252
C	-3.6671	-1.9359	-0.0873	C	3.5505	-1.9348	0.2703
H	-3.4291	-2.9900	-0.1655	H	3.3059	-2.9901	0.2501
C	-4.9888	-1.5089	0.0357	C	4.8501	-1.5081	0.5480
H	-5.7868	-2.2418	0.0540	H	5.6209	-2.2424	0.7503
C	-5.2983	-0.1457	0.1319	C	5.1703	-0.1452	0.5676
H	-6.3332	0.1618	0.2226	H	6.1860	0.1620	0.7858
C	-4.2899	0.8107	0.1131	C	4.1960	0.8139	0.3103
H	-4.5081	1.8691	0.1905	H	4.4249	1.8728	0.3187
C	-2.9644	0.3857	0.0003	C	2.8952	0.3900	0.0361
C	-1.7826	1.2803	-0.0213	C	1.7441	1.2839	-0.2432
C	-0.5257	0.4833	-0.1678	C	0.4841	0.4816	-0.3374
C	0.7793	0.8476	-0.2693	C	-0.8260	0.8305	-0.2875
C	1.7590	-0.2481	-0.5757	C	-1.8260	-0.2843	-0.4077
C	2.5465	-0.8194	0.4425	C	-2.3821	-0.8666	0.7484
C	3.4527	-1.8363	0.1348	C	-3.3362	-1.8785	0.6323
C	3.5837	-2.2778	-1.1818	C	-3.7417	-2.3082	-0.6321
C	2.8141	-1.7196	-2.1963	C	-3.1978	-1.7441	-1.7798
H	2.9126	-2.0649	-3.2182	H	-3.5060	-2.0840	-2.7610
C	1.9012	-0.7127	-1.8843	C	-2.2380	-0.7389	-1.6605
C	1.3485	2.2854	-0.1553	C	-1.3943	2.2657	-0.1470
H	1.2853	-0.2793	-2.6638	H	-3.7656	-2.3339	1.5140
H	4.0542	-2.2885	0.9113	H	-1.7953	-0.3042	-2.5482
C	2.8914	2.2975	-0.2359	C	-1.0395	3.0693	-1.4230
H	3.3456	1.7542	0.5932	H	-1.4627	2.5937	-2.3121
H	3.2597	1.8766	-1.1733	H	-1.4710	4.0716	-1.3435
H	3.2294	3.3355	-0.1807	H	0.0367	3.1641	-1.5488
C	0.8245	3.1274	-1.3461	C	-0.8332	2.9577	1.1172
H	1.2547	4.1314	-1.2850	H	0.2428	3.1019	1.0666
H	1.1378	2.6885	-2.2976	H	-1.3006	3.9413	1.2194
H	-0.2588	3.2183	-1.3345	H	-1.0751	2.3723	2.0069
C	0.9518	2.9214	1.1969	C	-2.9335	2.2562	-0.0163
H	-0.1251	3.0309	1.2959	H	-3.4204	1.8076	-0.8838
H	1.3287	2.3145	2.0227	H	-3.2622	1.7250	0.8786
H	1.4043	3.9149	1.2689	H	-3.2788	3.2901	0.0635
O	2.3559	-0.3125	1.6893	O	-1.9202	-0.3755	1.9330
C	2.9998	-0.9461	2.7959	C	-2.4281	-0.9263	3.1499
H	2.6543	-0.4153	3.6804	H	-2.2034	-1.9942	3.2236
H	2.7108	-1.9982	2.8650	H	-1.9191	-0.3926	3.9496
H	4.0883	-0.8631	2.7247	H	-3.5073	-0.7698	3.2359
H	4.2927	-3.0666	-1.4061	H	-4.4850	-3.0934	-0.7094

Supplementary Table 7 | Calculated ground state geometries of the transition states of *E*-1.

Transition state *E*-**1** (OMe over *t*Bu)				Transition state *E*-**1** (OMe over carbonyl)			
S	1.6330	-1.3348	-0.7356	S	1.5129	-1.7614	-0.2274
O	1.1124	1.8125	1.5764	O	0.9530	1.1491	2.1031
O	1.2845	-1.1952	-2.2123	O	1.0871	-2.2107	-1.6126
C	3.1147	-0.3570	-0.4105	C	2.7546	-0.4652	-0.4204
C	4.3783	-0.6586	-0.9020	C	3.9205	-0.5996	-1.1643
H	4.5445	-1.5134	-1.5466	H	4.1125	-1.4861	-1.7566
C	5.4339	0.1724	-0.5258	C	4.8366	0.4496	-1.1173
H	6.4336	-0.0368	-0.8880	H	5.7522	0.3855	-1.6934
C	5.2178	1.2659	0.3220	C	4.5953	1.5788	-0.3227
H	6.0539	1.8941	0.6057	H	5.3288	2.3759	-0.2944
C	3.9424	1.5542	0.7988	C	3.4286	1.6864	0.4274
H	3.7558	2.4001	1.4493	H	3.2346	2.5534	1.0472
C	2.8815	0.7369	0.4083	C	2.4873	0.6590	0.3477
C	1.4555	0.9475	0.7747	C	1.1750	0.5959	1.0385
C	0.6035	-0.0989	0.1857	C	0.3084	-0.4476	0.4201
C	-0.7688	-0.3541	0.2832	C	-1.0397	-0.6898	0.2985
C	-1.7376	0.7393	0.0022	C	-2.0115	0.3304	-0.2043
C	-1.2310	2.0556	-0.1672	C	-1.9191	1.7446	-0.0633
C	-2.0141	3.1709	-0.3846	C	-2.8456	2.5975	-0.6763
C	-3.3943	3.0244	-0.5045	C	-3.8575	2.0911	-1.4804
C	-3.9453	1.7560	-0.4553	C	-3.9483	0.7185	-1.6818
C	-3.1509	0.6185	-0.2470	C	-3.0474	-0.1254	-1.0477
C	-1.2022	-1.7845	0.7647	C	-1.6058	-2.1252	0.6696
H	-1.5485	4.1443	-0.4758	H	-4.7101	0.3002	-2.3281
H	-0.1726	2.2130	-0.1054	H	-2.7656	3.6653	-0.5311
H	-5.0084	1.6394	-0.6027	H	-3.1314	-1.1784	-1.2489
C	-1.7058	-2.7006	-0.3765	C	-0.6903	-2.7854	1.7375
H	-2.4960	-2.2490	-0.9656	H	0.2511	-3.1573	1.3374
H	-0.8791	-2.9529	-1.0450	H	-0.4648	-2.0900	2.5492
H	-2.0822	-3.6337	0.0535	H	-1.2142	-3.6455	2.1601
C	-2.2383	-1.5898	1.9153	C	-1.7786	-3.1447	-0.4854
H	-2.5519	-2.5797	2.2571	H	-1.9934	-4.1252	-0.0505
H	-1.7579	-1.0864	2.7587	H	-2.6072	-2.9089	-1.1530
H	-3.1269	-1.0386	1.6418	H	-0.8733	-3.2216	-1.0856
C	-0.0589	-2.5584	1.4835	C	-2.9612	-1.9259	1.4065
H	0.4622	-1.9290	2.2083	H	-2.8569	-1.2185	2.2336
H	-0.5109	-3.3908	2.0279	H	-3.7633	-1.5735	0.7628
H	0.6724	-2.9852	0.8041	H	-3.2663	-2.8877	1.8259
O	-3.7040	-0.6089	-0.3305	O	-0.9064	2.2128	0.6831
C	-5.0967	-0.7698	-0.6203	C	-0.7482	3.6131	0.9025
H	-5.7173	-0.3295	0.1641	H	-0.5932	4.1490	-0.0383
H	-5.3502	-0.3329	-1.5891	H	-1.6121	4.0290	1.4285
H	-5.2581	-1.8451	-0.6511	H	0.1357	3.7017	1.5298
H	-4.0349	3.8829	-0.6696	H	-4.5593	2.7695	-1.9517

Supplementary Table 8 | Calculated ground state geometries of the transition states of *Z*-1.

Transition state *Z*-**1** (OMe over *t*Bu)				Transition state *Z*-**1** (OMe over sulfoxide)			
S	1.4223	1.4954	0.4255	S	0.9301	-1.4973	0.3991
O	1.3607	-1.9008	-1.5193	O	1.3405	1.4827	-1.9728
O	1.2028	1.5894	1.9277	O	1.1030	-2.5678	-0.6720
C	3.0440	0.7732	0.1066	C	2.6092	-0.7755	0.4312
C	4.2507	1.3747	0.4430	C	3.6918	-1.3228	1.1016
H	4.2836	2.3131	0.9833	H	3.5707	-2.1467	1.7951
C	5.4235	0.7287	0.0541	C	4.9553	-0.7802	0.8481
H	6.3824	1.1668	0.3046	H	5.8200	-1.1772	1.3670
C	5.3768	-0.4721	-0.6668	C	5.1192	0.2589	-0.0736
H	6.3021	-0.9499	-0.9661	H	6.1094	0.6582	-0.2582
C	4.1582	-1.0542	-0.9997	C	4.0207	0.7864	-0.7508
H	4.1047	-1.9825	-1.5555	H	4.1311	1.5938	-1.4652
C	2.9819	-0.4265	-0.5869	C	2.7590	0.2663	-0.4750
C	1.6059	-0.9386	-0.7988	C	1.4606	0.7461	-1.0074
C	0.6015	-0.0674	-0.1600	C	0.3181	0.1395	-0.2605
C	-0.7412	-0.3788	0.0281	C	-0.8628	0.7949	0.0309
C	-1.8214	0.6377	-0.1732	C	-2.2279	0.1844	0.0962
C	-1.4592	1.9867	-0.4209	C	-2.5355	-1.2100	0.0059
C	-2.3519	3.0293	-0.5792	C	-3.8577	-1.6699	-0.0602
C	-3.7178	2.7665	-0.5465	C	-4.9272	-0.7896	-0.0679
C	-4.1442	1.4572	-0.4108	C	-4.6759	0.5759	-0.0199
C	-3.2374	0.3960	-0.2653	H	-5.4861	1.2939	-0.0503
C	-1.0212	-1.8124	0.6182	C	-3.3686	1.0263	0.0630
H	-1.9793	4.0330	-0.7412	C	-0.7463	2.3499	0.3514
H	-5.2027	1.2460	-0.4353	H	-4.0483	-2.7316	-0.1041
H	-0.4198	2.2465	-0.5080	H	-3.2403	2.0896	0.0746
C	-1.9218	-1.5996	1.8767	C	0.6991	2.8339	0.6561
H	-1.3891	-0.9869	2.6090	H	1.2217	2.1601	1.3382
H	-2.8833	-1.1461	1.6779	H	1.3036	2.9846	-0.2325
H	-2.1013	-2.5776	2.3307	H	0.6258	3.8047	1.1510
C	0.2409	-2.4823	1.2306	C	-1.4496	2.6033	1.7279
H	-0.0954	-3.3270	1.8368	H	-1.3796	3.6696	1.9556
H	0.9217	-2.8786	0.4839	H	-2.4926	2.3168	1.7959
H	0.7836	-1.7983	1.8865	H	-0.9135	2.0631	2.5134
C	-1.5944	-2.8217	-0.4044	C	-1.2561	3.2679	-0.7887
H	-2.4456	-2.4379	-0.9552	H	-0.6099	3.1387	-1.6583
H	-0.8084	-3.0885	-1.1113	H	-2.2779	3.0831	-1.1123
H	-1.9075	-3.7278	0.1241	H	-1.1928	4.3115	-0.4665
O	-3.6933	-0.8737	-0.2551	O	-1.5126	-2.0843	-0.0161
C	-5.0913	-1.1555	-0.3697	C	-1.7527	-3.4803	-0.2540
H	-5.6497	-0.7392	0.4724	H	-2.2931	-3.9274	0.5839
H	-5.4951	-0.7761	-1.3116	H	-2.3117	-3.6218	-1.1818
H	-5.1643	-2.2408	-0.3529	H	-0.7614	-3.9109	-0.3509
H	-4.4447	3.5626	-0.6563	H	-5.9402	-1.1716	-0.1171

Crystal structure analysis

Supplementary Table 9 | Crystal structure analysis of A and B.

Compound	**A-1**<br>CCDC 1586011	**B-1**<br>CCDC 1586012
net formula	$C_{20}H_{20}O_3S$	$C_{20}H_{20}O_3S$
M_r/g mol^{-1}	340.42	340.42
crystal size/mm	0.100 × 0.080 × 0.050	0.100 × 0.060 × 0.030
T/K	100.(2)	100.(2)
radiation	MoKα	MoKα
diffractometer	'Bruker D8 Venture TXS'	'Bruker D8 Venture TXS'
crystal system	triclinic	orthorhombic
space group	'P -1'	'P b c a'
a/Å	8.3514(4)	13.0489(3)
b/Å	13.3861(6)	15.1168(4)
c/Å	15.2817(7)	16.9239(4)
α/°	83.607(2)	90
β/°	86.533(2)	90
γ/°	87.048(2)	90
V/Å^3	1692.92(14)	3338.37(14)
Z	4	8
calc. density/g cm^{-3}	1.336	1.355
μ/mm^{-1}	0.206	0.209
absorption correction	Multi-Scan	Multi-Scan
transmission factor range	0.8916–0.9705	0.9044–0.9705
refls. measured	19349	31719
R_{int}	0.0387	0.0534
mean σ(I)/I	0.0448	0.0266
θ range	3.211–26.372	3.170–26.373
observed refls.	5350	2779
x, y (weighting scheme)	0.0312, 1.4998	0.0324, 2.8521
hydrogen refinement	constr	constr
Flack parameter	-	-
refls in refinement	6838	3406
parameters	441	221
restraints	0	0
$R(F_{obs})$	0.0420	0.0347
$R_w(F^2)$	0.1041	0.0920
S	1.039	1.070
shift/error$_{max}$	0.001	0.001
max electron density/e Å^{-3}	0.391	0.345
min electron density/e Å^{-3}	−0.446	−0.374

Supplementary Table 10 | Crystal structure analysis of C and D.

Compound	**C-1**<br>CCDC 1586013	**D-1**<br>CCDC 1586014
net formula	$C_{20}H_{20}O_3S$	$C_{20}H_{20}O_3S$
M_r/g mol^{-1}	340.42	340.42
crystal size/mm	0.080 × 0.070 × 0.030	0.100 × 0.090 × 0.060
T/K	100.(2)	100.(2)
radiation	MoKα	MoKα
diffractometer	'Bruker D8 Venture TXS'	'Bruker D8 Venture TXS'
crystal system	triclinic	orthorhombic
space group	'P -1'	'P 21 21 21'
a/Å	10.0205(3)	10.3115(3)
b/Å	10.8597(3)	12.0645(3)
c/Å	16.2287(5)	13.9574(3)
α/°	81.7640(10)	90
β/°	80.7990(10)	90
γ/°	85.3530(10)	90
V/Å^3	1722.24(9)	1736.34(8)
Z	4	4
calc. density/g cm^{-3}	1.313	1.302
μ/mm^{-1}	0.203	0.201
absorption correction	Multi-Scan	Multi-Scan
transmission factor range	0.9339–0.9705	0.9059–0.9705
refls. measured	32659	15544
R_{int}	0.0536	0.0386
mean σ(I)/I	0.0406	0.0311
θ range	3.134–26.372	3.372–26.367
observed refls.	5647	3365
x, y (weighting scheme)	0.0304, 1.7686	0.0167, 0.6551
hydrogen refinement	constr	constr
Flack parameter	-	0.21(8)
refls in refinement	7025	3527
parameters	441	222
restraints	0	0
$R(F_{obs})$	0.0417	0.0271
$R_w(F^2)$	0.1012	0.0644
S	1.023	1.080
shift/error$_{max}$	0.001	0.001
max electron density/e Å^{-3}	0.891	0.222
min electron density/e Å^{-3}	−0.430	−0.217

Supplementary References

1. A. Gerwien, T. Reinhardt, P. Mayer, H. Dube, Versatile Synthesis of Double-Bond Substituted Hemithioindigo Photoswitches. *Org. Lett.* **1**, 232-235 (2018).

2. U. Megerle, R. Lechner, B. König, E. Riedle, Laboratory apparatus for the accurate, facile and rapid determination of visible light photoreaction quantum yields. *Photochem. Photobiol. Sci.* **9**, 1400-1406 (2010).

3. Gaussian 09, Revision A.02, M. J. Frisch, G. W. Trucks, H. B. Schlegel, G. E. Scuseria, M. A. Robb, J. R. Cheeseman, G. Scalmani, V. Barone, G. A. Petersson, H. Nakatsuji, X. Li, M. Caricato, A. Marenich, J. Bloino, B. G. Janesko, R. Gomperts, B. Mennucci, H. P. Hratchian, J. V. Ortiz, A. F. Izmaylov, J. L. Sonnenberg, D. Williams-Young, F. Ding, F. Lipparini, F. Egidi, J. Goings, B. Peng, A. Petrone, T. Henderson, D. Ranasinghe, V. G. Zakrzewski, J. Gao, N. Rega, G. Zheng, W. Liang, M. Hada, M. Ehara, K. Toyota, R. Fukuda, J. Hasegawa, M. Ishida, T. Nakajima, Y. Honda, O. Kitao, H. Nakai, T. Vreven, K. Throssell, J. A. Montgomery, Jr., J. E. Peralta, F. Ogliaro, M. Bearpark, J. J. Heyd, E. Brothers, K. N. Kudin, V. N. Staroverov, T. Keith, R. Kobayashi, J. Normand, K. Raghavachari, A. Rendell, J. C. Burant, S. S. Iyengar, J. Tomasi, M. Cossi, J. M. Millam, M. Klene, C. Adamo, R. Cammi, J. W. Ochterski, R. L. Martin, K. Morokuma, O. Farkas, J. B. Foresman, and D. J. Fox, Gaussian, Inc., Wallingford CT, 2016.
